# Mitochondrial state determines functionally divergent stem cell population in planaria

**DOI:** 10.1016/j.stemcr.2021.03.022

**Published:** 2021-04-15

**Authors:** Mohamed Mohamed Haroon, Vairavan Lakshmanan, Souradeep R. Sarkar, Kai Lei, Praveen Kumar Vemula, Dasaradhi Palakodeti

**Affiliations:** 1 Integrative Chemical Biology, Institute for Stem Cell Science and Regenerative Medicine, Bengaluru, India; 2 SASTRA University, Thirumalaisamudram, Thanjavur, India; 3 National Centre for Biological Sciences, Bengaluru, India; 4 Zhejiang Provincial Laboratory of Life Sciences and Biomedicine, Key Laboratory of Growth Regulation and Translational Research of Zhejiang Province, School of Life Sciences, Westlake University, Hangzhou, Zhejiang Province, China; 5 Institute of Biology, Westlake Institute for Advanced Study, Hangzhou, Zhejiang Province, China

**Keywords:** planaria, neoblasts, stem cells, pluripotency, mitochondria, differentiation, FACS

## Abstract

Mitochondrial state changes were shown to be critical for stem cell function. However, variation in the mitochondrial content in stem cells and the implication, if any, on differentiation is poorly understood. Here, using cellular and molecular studies, we show that the planarian pluripotent stem cells (PSCs) have low mitochondrial mass compared with their progenitors. Transplantation experiments provided functional validation that neoblasts with low mitochondrial mass are the true PSCs. Further, the mitochondrial mass correlated with OxPhos and inhibiting the transition to OxPhos dependent metabolism in cultured cells resulted in higher PSCs. In summary, we show that low mitochondrial mass is a hallmark of PSCs in planaria and provide a mechanism to isolate live, functionally active, PSCs from different cell cycle stages (G0/G1 and S, G2/M). Our study demonstrates that the change in mitochondrial metabolism, a feature of PSCs is conserved in planaria and highlights its role in organismal regeneration.

## Highlights

• Mitochondrial state differs between stem (X1) and differentiated (Xins) cells
• X1 cells with low MTG are enriched for pluripotent cells compared with high MTG cells
• MTG-based sorting yields functional neoblasts from G1, S/G2/M phase of cell cycle
• Inhibition of mitochondrial activity affects neoblast differentiation *in vitro*

## Teaser

Palakodeti and colleagues have found that planarian pluripotent stem cells have low mitochondrial mass and activity compared with their progenitors. Using nuclear and mitochondrial dyes they provide approaches to isolate functional stem cells, which can potentially be used for other model systems. Subsequently, they provide evidence for the causal role of mitochondrial activity in stem cell function.

## Introduction

Neoblasts are adult pluripotent stem cells (PSCs) that play a central role in planarian regeneration. They are capable of differentiating into all other cell types, thereby facilitating the regeneration of lost tissues. On injury, planarians induce wound healing response leading to stem cell proliferation and differentiation, followed by remodeling of old and new tissue to form a completely regenerated animal (Reddien, 2018). In planarians, neoblasts are the sole proliferating cells and eliminating them by irradiation results in lethality (Newmark and Sánchez Alvarado, 2000; Reddien and Alvarado, 2004). Our understanding of the mechanisms that regulate pluripotency is limited by the capacity to isolate the PSCs from planaria.

The current technique to isolate neoblasts involves fluorescence-activated cell sorting (FACS) based on nuclear density by staining the cells with Hoechst (Hayashi et al., 2006). Hoechst staining identified three major populations: X1, X2, and Xins. X1 cells are the proliferating neoblasts with >2N nuclear content (S and G2/M phases of the cell cycle) and expression of stem cell markers such as *piwi-1, vasa-1, bruli* (Reddien et al., 2005; Wagner et al., 2012). X2 includes post-mitotic progenitor cells as well as stem cells in the G0/G1 phase of the cell cycle (Eisenhoffer et al., 2008; Molinaro and Pearson, 2016; Van Wolfswinkel et al., 2014). Both X1, X2 are eliminated on X-ray irradiation, whereas the Xins population is insensitive to irradiation and constitutes terminally differentiated cells. Since the neoblasts are the only dividing cells in planaria, the X1 population has been extensively used for functional characterization of the neoblast. So far, our understanding of the stem cells in the X2 gate was limited, as it has not been possible to isolate stem cells from this gate. Therefore, the current knowledge about the neoblasts was obtained by studying the X1 population (>2N cells).

Detailed molecular analysis showed that the X1 population consists of pluripotent (clonogenic neoblasts) and primed stem cells (specialized neoblasts) (Fincher et al., 2018; Plass et al., 2018; Van Wolfswinkel et al., 2014; Wagner et al., 2011). Clonogenic neoblasts were shown to rescue planarians upon lethal irradiation. Recently, it was shown that a subset of neoblasts, which express tetraspanin-1 (TSPAN-1) are pluripotent in nature. Single-cell transplantation of TSPAN-1+ cells in lethally irradiated planarians rescued the host with greater efficiency validating its pluripotency (Zeng et al., 2018). The specialized neoblasts are defined by the expression of transcripts pertaining to specific lineages in addition to pan neoblast markers such as *piwi-1*. However, a lack of strong evidence to show that all the PSCs are TSPAN + suggests that there could be a possibility of a subset of PSCs that lack the expression of TSPAN.

Mitochondria, a hub for cellular energetics, have been shown to be critical for stem cell maintenance and differentiation (Folmes et al., 2012; Xu et al., 2013). Evidence shows that mitochondria in PSCs exist in a discontinuous fission state with very low oxidative phosphorylation (OxPhos). Several studies have used differences in mitochondrial activity as a means to demarcate stem cells from their respective progenitors. For example, hematopoietic stem cells (HSCs) with low MitoTrackerTM Green (MTG) staining exhibited enhanced stemness and showed greater reconstitution potential compared with their MTG High counterpart (Romero-Moya et al., 2013). Similarly, tetramethylrhodamine methyl ester (TMRM) a mitochondrial membrane potential sensing dye was used to separate minimally differentiated stem cell-memory T cells from highly differentiated effector memory T cells (Sukumar et al., 2016).

This study aimed to understand the differences in mitochondrial content between the planarian cell populations, its functional consequence on stemness, and employ this as a means to distinguish stem cell states. Based on the mitochondrial dye MTG, we were able to further distinguish cells within the X1 and the X2 cell populations as X1-MTG^High^, X1-MTG^Low^, X2-MTG^High^, and X2-MTG^Low^ cells. Transcriptome analysis and molecular characterization of these cells revealed that the X1 cells with low mitochondrial mass were a more homogeneous pool of PSCs compared with cells with higher mitochondrial mass. The X1-MTG^High^ cells expressed genes essential for lineage commitment exhibiting the signatures of committed neoblasts. A similar analysis of X2 revealed that the cells with low mitochondrial mass were either stem cells in the G0/G1 phase or early progenitors, whereas the cells with high mitochondrial mass were late progenitors. Further, to validate the pluripotency, cells isolated from the asexual strain (donor) were transplanted into lethally irradiated sexual strain (host). Our results indicate that the X1 and a subset of X2 cells with low MTG were able to rescue and thus transform the sexual planaria into asexual planaria at higher efficiencies compared with the MTG^High^ cells. Our study also revealed that the mitochondrial activity correlates with mass and inhibiting the mitochondrial activity by treating the neoblast with FCCP *in vitro* resulted in increased stemness as validated by the transplantation experiments. Together, our results show that the PSCs in the planarians can be categorized by low mitochondrial mass compared with their immediate progenitors. Further, the changes in the mitochondrial mass between pluripotent neoblast and the committed neoblast could be used to isolate PSCs from planarians for functional characterization.

## Results

### Mitochondrial staining reveals a distinct mitochondrial mass in planarian X1, X2, Xins cells

**Figure 1:**
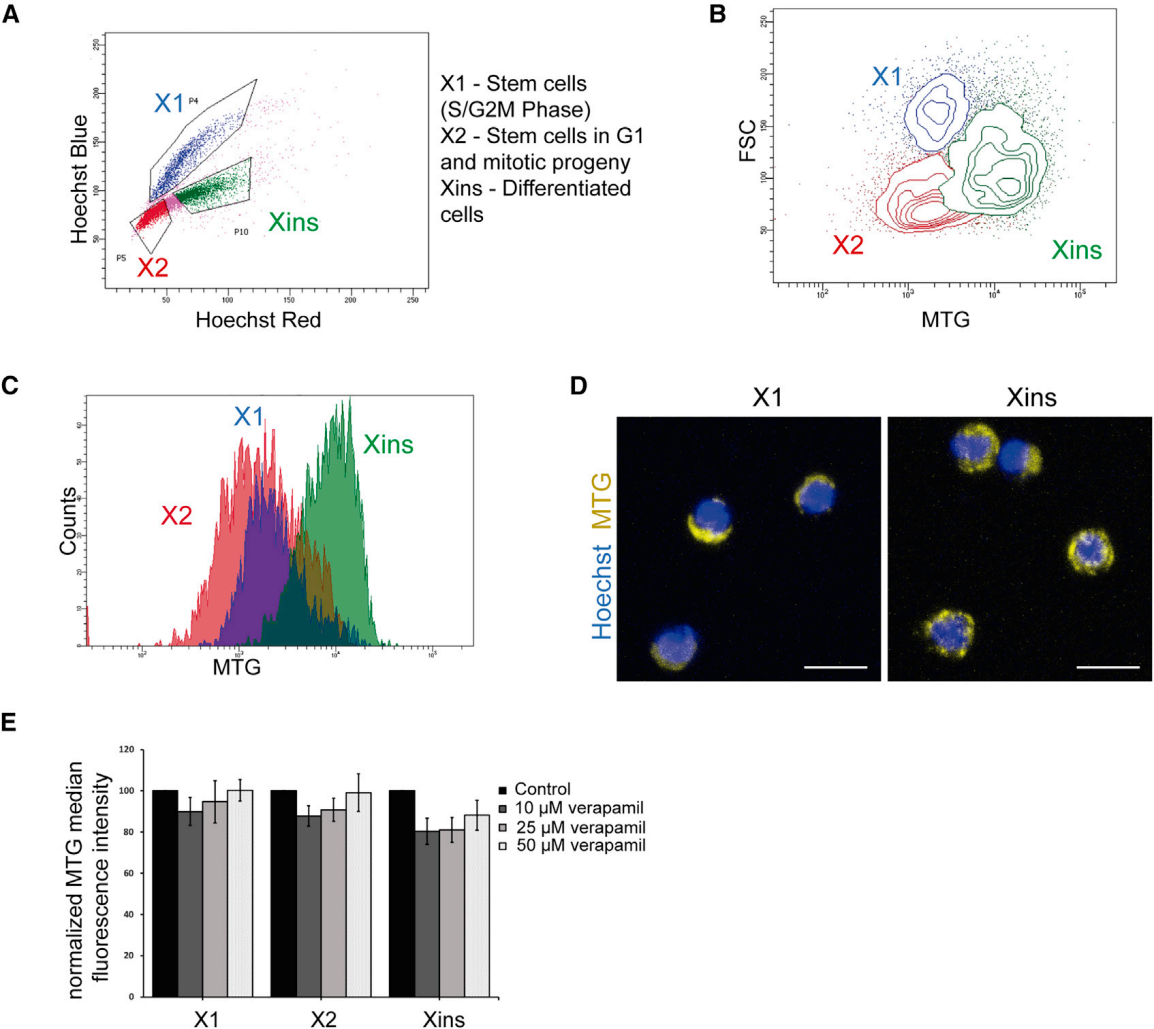
MitoTracker Green FM staining in planarian cells (A) Flow cytometry analysis of planarian cell suspension stained with Hoechst 33,342 revealing X1, X2, and Xins population. (B and C) Representative contour plot (B) and histogram (C) showing MTG intensity of X1, X2, and Xins cells. (D) Representative confocal images showing MTG staining in sorted, live X1, and Xins cells. Scale bar, 10 μm. (E) Flow cytometry analysis of MTG median fluorescent intensity of X1, X2, and Xins cells in the presence of indicated amounts of Verapamil. Mean ± SEM, over three independent replicates.

Single-cell transcriptome studies in planarians revealed neoblasts as a heterogeneous pool of clonogenic and specialized stem cells. Here, we investigated how the mitochondrial mass changes between different planarian cell populations. This was tested by staining the neoblast population with MTG, a fluorescent dye that is used as a measure for the mitochondrial mass (Doherty and Perl, 2017; Romero-Moya et al., 2013). The cell suspension of planaria was stained with Hoechst 33,342 (nuclear dye) and MTG. The cells stained with Hoechst showed three distinct populations, X1, X2, and Xins, based on their nuclear content (Figure 1A). X1 cells, which are the proliferating stem cells, have a lower MTG signal than the Xins population (differentiated cells) (Figures 1B and 1C). The X2 population, majorly neoblast progenitor, had a broad distribution of the MTG signal (Figures 1B and 1C). MTG fluorescence in most X1 cells was more perinuclear and exhibited polarized distribution in the cytoplasm (Figures 1D and S1). Conversely, MTG fluorescence in Xins cells, in general, was evenly distributed in the cytoplasm (Figures 1D and S1). To test whether the low MTG intensity in X1 and X2 cells was a consequence of dye efflux, the cells were treated with Verapamil, an efflux pump inhibitor, and stained with MTG. There was no significant increase in the median fluorescence intensity in the X1 population on treatment with Verapamil (Figure 1E), suggesting that the lower MTG fluorescence in X1 cells is a true indicator of decreased mitochondrial mass. Together, these results suggest that the difference in the mitochondrial mass between X1 and Xins cells could be an indicator of the stem versus differentiated state.

It has been shown that mammalian embryonic stem cells show distinct mitochondrial morphology and their activity compared with their progenitors (Xu et al., 2013). However, the change in the mitochondrial content and its regulation of pluripotency was poorly understood. We examined whether the mitochondrial mass could distinguish the clonogenic and specialized neoblast pools. To this end, molecular and functional studies were performed in X1 cells with high and low mitochondrial mass as measured by MTG.

### Low MTG enriches for PIWI-1^High^ cells within the X1 population

**Figure 2:**
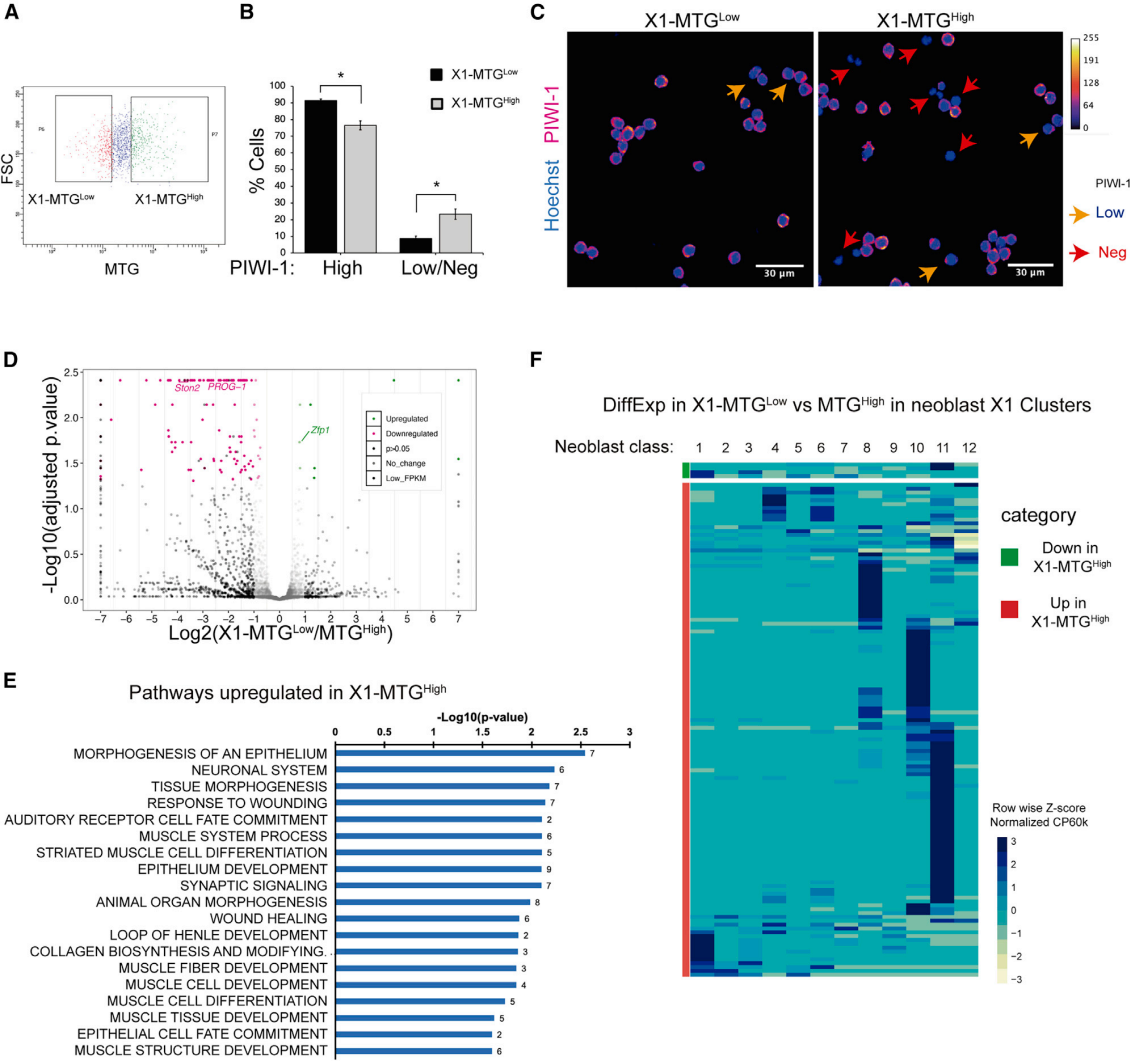
MTG staining reveals heterogeneity in X1 cells (A) Dot plot showing the FACS gating for X1-MTG^Low^ and MTG^High^ cells. (B) PIWI-1 immunostaining in FACS sorted X1-MTG^Low^ and MTG^High^ cells quantified for PIWI-1^High^ through flow cytometry. Mean ± SEM, over three independent replicates. ^∗^Represents p < 0.05. See also Figure S1. (C) Representative confocal images of PIWI-1 staining in indicated sorted population. PIWI-1 staining is shown using Fire LUT (ImageJ). Arrowheads highlight PIWI-1 low (yellow) and negative (red) cells. Scale bar, 30 μm. (D) Volcano plot displaying differential gene expression between X1-MTG^Low^ and MTG^High^ cells. (E) Bar graph representing selected GO terms that are downregulated in X1-MTG^Low^ cells compared with MTG^High^. Refer to Table S1 for the full list of GO terms. (F) Heatmap indicating the expression of the differentially expressed genes in X1-MTG^Low^ vs MTG^High^ cells in the published single-cell database (Zeng et al., Cell 2018).

In addition to the change in the mitochondrial mass between X1 and Xins, we also identified cells with high and low MTG signals within the X1 population (Figure 2A). Here, we wanted to investigate the pluripotent state of the MTG^Low^ and MTG^High^ cells in the X1 population. *Piwi-1* is a nuage-related gene that has been extensively used to mark stem cells in planaria. To examine the status of *piwi-1* in MTG^Low^ and MTG^High^ cells, we first generated and characterized an antibody to PIWI-1 (Guo et al., 2006). Previous studies have shown that clonogenic neoblast and a subset of the neoblast population have high levels of PIWI-1 protein and its corresponding RNA, whereas the progenitors have low levels of PIWI-1 protein (Zeng et al., 2018). The immuno-staining with PIWI-1 antibody in planarian cells identified High, Low, and negative PIWI-1 cells (Figures S2A and S2B). Further, we have also shown that PIWI-1^High^ cells are the proliferating cells, which are in the S, G2/M phase, whereas most of the PIWI-1^Low/Negative^ cells are in the G1 phase as was reported in earlier studies (Zeng et al., 2018) (Figure S2C). This was also validated by immunostaining of X1, X2, and Xins cells for PIWI-1 protein, where X1 cells showed high PIWI-1 compared with X2 and Xins (Figures S2D and S2E). Together these results show that the PIWI-1 antibody recognizes the neoblast population.

Next, we examined the expression of PIWI-1 in MTG^High^ and MTG^Low^ cells within the X1 population (Figure 2A). We observed that X1-MTG^Low^ cells had a higher representation of PIWI-1^High^ cells compared with X1-MTG^High^ cells (Figures 2B and 2C). Conversely, cells from X1-MTG^High^ cells were enriched in PIWI-1^Low/Negative^ cells. We speculate that enrichment of PIWI-1^Low/Negative^ cells in X1-MTG^High^ could be the committed NB clusters and the X1-MTG^Low^ cells could either be the noncommitted neoblast or the clonogenic population.

### Transcriptome sequencing of high and low MTG reveal neoblast heterogeneity within X1 cells

In order to understand the functional states of High and Low MTG cells, RNA sequencing (RNA-seq) was performed on X1-MTG^Low^ and X1-MTG^High^ populations (Figure 2D). Transcriptome sequencing followed by Cuffdiff (Trapnell et al., 2013) analysis for differential expression identified 172 differentially regulated genes from X1-MTG^Low^ and MTG^High^ cells (Figure 2D). Gene ontology (GO) analysis of upregulated (p < 0.05) genes (163) in X1-MTG^High^ cells showed that most of these genes are essential for differentiation (muscle, epithelium, neural system), wound response, and tissue morphogenesis (Figure 2E and Table S1). For instance, our analysis identified two key markers of epidermal and neural progenitors (*prog-1* and *ston-2)* upregulated by >2-fold (p < 0.05) in X1-MTG^High^ cells. Next, the differentially expressed genes were compared with the published single-cell transcriptome data from X1 (Zeng et al., 2018). Most of the genes enriched in X1-MTG^High^ have high expression in NB classes other than NB2, which is the clonogenic neoblast population, suggesting that the X1-MTG^High^ cells are mostly committed neoblasts (Figure 2F). Interestingly, we did not observe any genes significantly upregulated in the X1-MTG^Low^ population that belong to the NB2 class (clonogenic neoblast) such as *tspan-1*, *tgs-1,* and *pks-1*. Together, these results indicate that within X1, the MTG^High^ cells represent the committed neoblast and the MTG^Low^ cells could potentially contain pluripotent neoblast cells.

Encouraged by these results, we then explored the cell states of the X2 population based on MTG signal. Cells with low and high MTG signals within the X2 population were subjected to PIWI-1 immunostaining and transcriptome analysis.

### Low and high MTG X2 cells show heterogeneous pool of PIWI-1^+^ and PIWI-1^−^ populations, respectively

**Figure 3:**
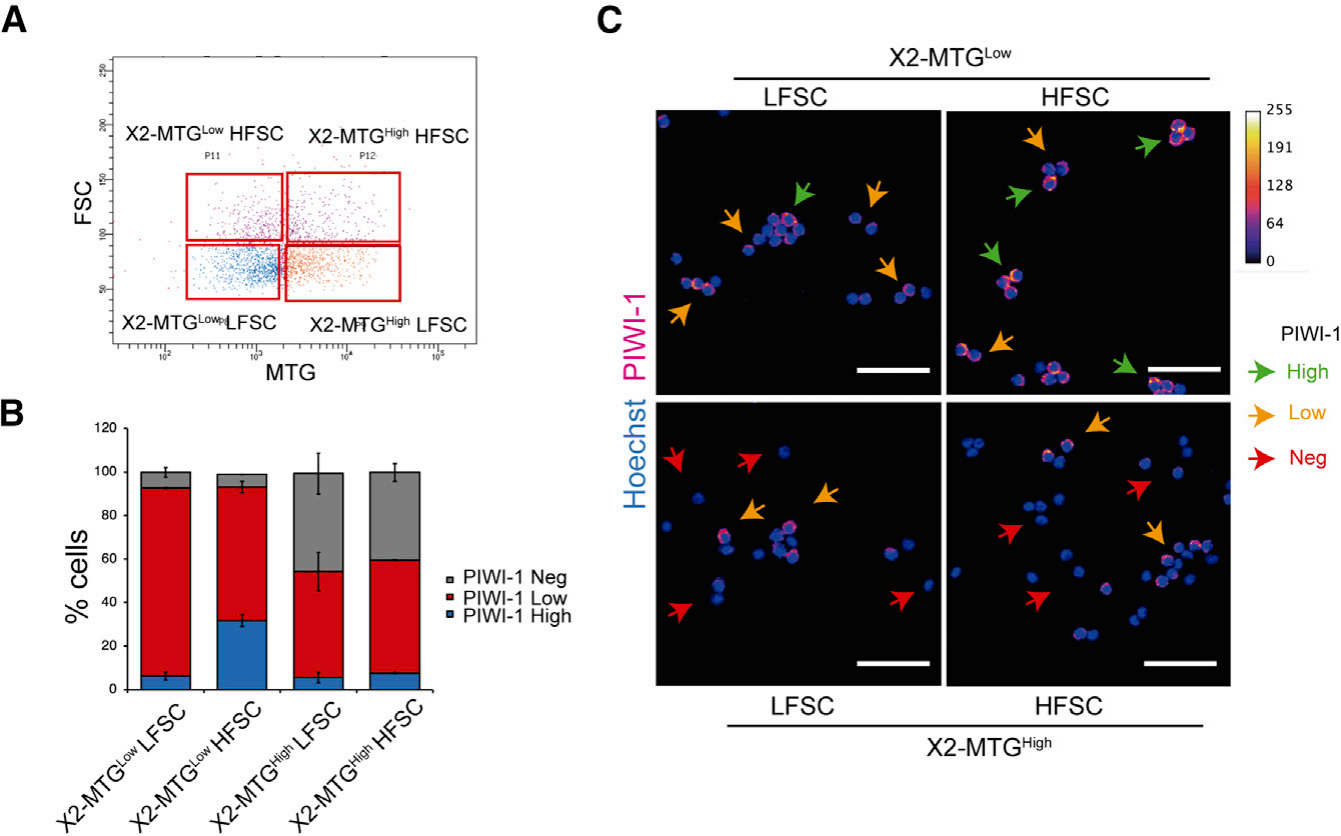
PIWI-1 immunostaining in X2-MTG subpopulations (A) Dot plot representing the gating strategy for various MTG populations. HFSC - High Forward Scatter, LFSC – Low Forward Scatter. (B) Quantitative flow cytometry analysis of PIWI-1 staining in indicated FACS sorted X2 MTG population. Mean ± SEM, over three independent replicates. (C) Representative confocal images of PIWI-1 staining in indicated X2 MTG populations. PIWI-1 staining is represented in Fire LUT (ImageJ) and the pixel value and the corresponding color code are given in the graph (top right). Arrowheads indicate PIWI-1 high (green), low (yellow), and negative (red) cells. Scale bar, 30 μm. See also Figure S1.

Previous studies showed that the X2 population consists of neoblasts and their progenitors (Eisenhoffer et al., 2008; Van Wolfswinkel et al., 2014). Our results based on MTG staining showed that the X2 population can be broadly categorized as MTG^Low^ and MTG^High^ cells. To investigate the stem state of the MTG^Low^ and MTG^High^ cells in the X2 population, we stained them with PIWI-1 antibody, which marks the neoblast populations. The X2-MTG^Low^ cells have a mixed population of PIWI-1^High^ and ^−Low^. Flow cytometry analysis based on the size showed PIWI-1^High^ cells are large in size compared with the PIWI-1 low cells (Figure S2A) (Zeng et al., 2018). Hence, to separate PIWI-1^High^ cells in the G1 phase, we further sorted the X2-MTG^Low^ and X2-MTG^High^ cells based on their size (FSC), which resulted in four populations (X2-MTG^Low^ High and Low FSC cells; X2-MTG^High^ High and Low FSC cells) (Figure 3A). The antibody staining indicated that within the X2-MTG^Low^, ∼35% of HFSC (High FSC) cells showed higher expression of PIWI-1, while LFSC (Low FSC) cells had lower PIWI-1 expression (∼85% cells) (Figures 3B and 3C). Regardless of the size, the majority of the X2-MTG^High^ cells either showed low or negative expression for PIWI-1 (Figures 3B and 3C). Together, these data suggest that X2-MTG^Low^-(HFSC) cells, which were PIWI-1^High^, are likely to be neoblast population in the G1 phase. Whereas the X2-MTG^Low^-(LFSC) cells, which are PIWI-1^Low^ could be the early progenitors. Conversely, X2-MTG^High^ (High and Low FSC) cells that are PIWI-1 negative could potentially be late progenitors.

### Transcriptome sequencing shows enrichment of neoblast and their early progenitors in the X2-MTG^Low^ population

**Figure 4:**
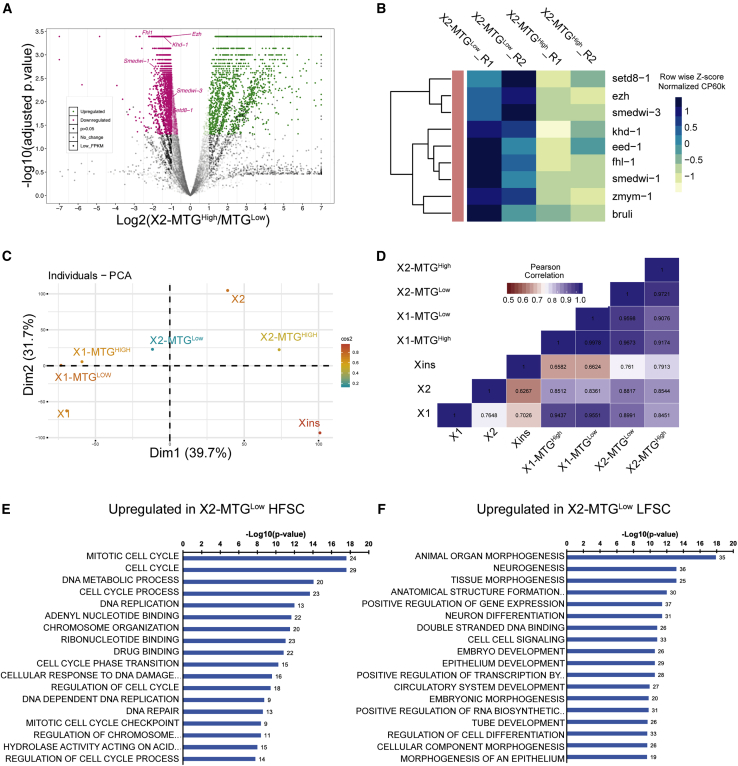
Transcriptome analysis of X2-MTG^Low^ and MTG^High^ cells (A) Volcano plot showing the differentially expressed transcripts. Major stem cell genes >2-fold upregulated in MTG Low are marked in the plot. (B) Heatmap showing expression of key stem cell-related genes in X2-MTG^Low^ and MTG^High^. R1 and R2 represent biological replicates. (C) Principal component analysis (PCA) showing indicated MTG population with respect to X1, X2, and Xins (D) Pearson's correlation heatmap of the X2 MTG population compared with X1, X2, and Xins. See also Figures S2 and S3 and Table S2. (E) GO terms of statistically significant, >2-fold upregulated transcripts from X2-MTG^Low^-HFSC cells compared with X2-MTG^Low^-LFSC cells. (F) GO terms of upregulated transcripts from the X2-MTG^Low^-LFSC cells compared with corresponding HFSC cells. See also Table S3.

To gain further insights into the nature of the cells in the MTG^Low^ and MTG^High^ populations of X2, we performed RNA-seq on the four populations. RNA-seq analysis of X2-MTG^Low^ and MTG^High^ cells (High and Low FSC combined) revealed that the MTG^Low^ population is enriched with transcripts of stemness, nuage-related neoblast markers (*smedwi-1, smedwi-3, bruli*), cell cycle genes (*rrm2, mcm*), and early markers for differentiation compared with MTG^High^ (Figures 4A, 4B, and S3A and Table S2). Further, we also compared the RNA profile of the MTG subpopulations to the total X1, X2, and Xins populations isolated by conventional methods. Based on principal component analysis, correlation plot, and linkage mapping (Figures 4C, D, and S3B), the transcript signatures of X2-MTG^Low^ showed a strong correlation (*R*^2^ = 0.8991) with the X1 population compared with X2-MTG^High^, which was closer to Xins. Further, we also observed that most of the transcripts related to early epidermal progenitors (*prog-1* and *agat-1*) were enriched in X2-MTG^Low^, while the transcripts of late epidermal progenitors (*zpuf-6*) and the terminally differentiated epidermal cells were enriched in X2-MTG^High^ (Figures S3C). Moreover, pseudotime expression analysis using the available planarian single-cell RNA-seq database showed that the expression of transcripts enriched in X2-MTG^Low^ peaked in the neoblast class and decreased as the cells differentiated. In contrast, transcripts enriched in the X2-MTG^High^ showed negligible expression in neoblast populations and increased as the cells differentiate (Figures S4A–S4C). These analyses clearly show that the cells in X2-MTG^Low^ are either the neoblast cells in the G1 phase or their immediate/early progenitors. Conversely, the cells in X2-MTG^High^ were mostly in the late phase of differentiation. Thus, our results suggest that the changes in mitochondrial content reflect the cell transition states within X2.

Next, we analyzed the transcriptome data from both High and Low FSC events from X2 MTG^Low^. GO analysis showed that the differentially expressed genes that are upregulated (p < 0.05) in X2-MTG^Low^-(HFSC) cells are involved in cell cycle and DNA synthesis indicating these might be the stem cells in G0/G1 phase poised to potentially enter the cell cycle (Figure 4E). On the contrary, GO analysis for the transcripts enriched in X2-MTG^Low^-(LFSC) cells encode proteins that were involved in differentiation suggesting that these cells are the immediate/early progenitors (Figure 4F and Table S3). To rule out the possibility of S-phase cells contaminating the X2-MTG^Low^-(HFSC) gate, cell cycle analysis was performed in sorted cells. The results clearly indicate that both LFSC and HFSC cells from the X2-MTG^Low^ gate are in G0/G1 state and in contrast, the X1-MTG^Low^ cells are in S and G2 phase (Figure S5A). Further, the principal component analysis and correlation plot revealed that the transcripts from both the High and Low FSC cells of X2-MTG^Low^ show high correlation with the X1 population (*R*^2^ = 0.9 HFSC cells and *R*^2^ = 0.89 LFSC cells) suggesting that the X2-MTG^Low^-(HFSC) gate contains cells that are most likely the neoblast population in G1 phase (Figures S5B and S5C). Together, these results show that MTG along with Hoechst will be a good marker to identify and isolate neoblast cells in the G0/G1 phase.

### Pluripotency is associated with stem cells having low mitochondrial mass

**Figure 5:**
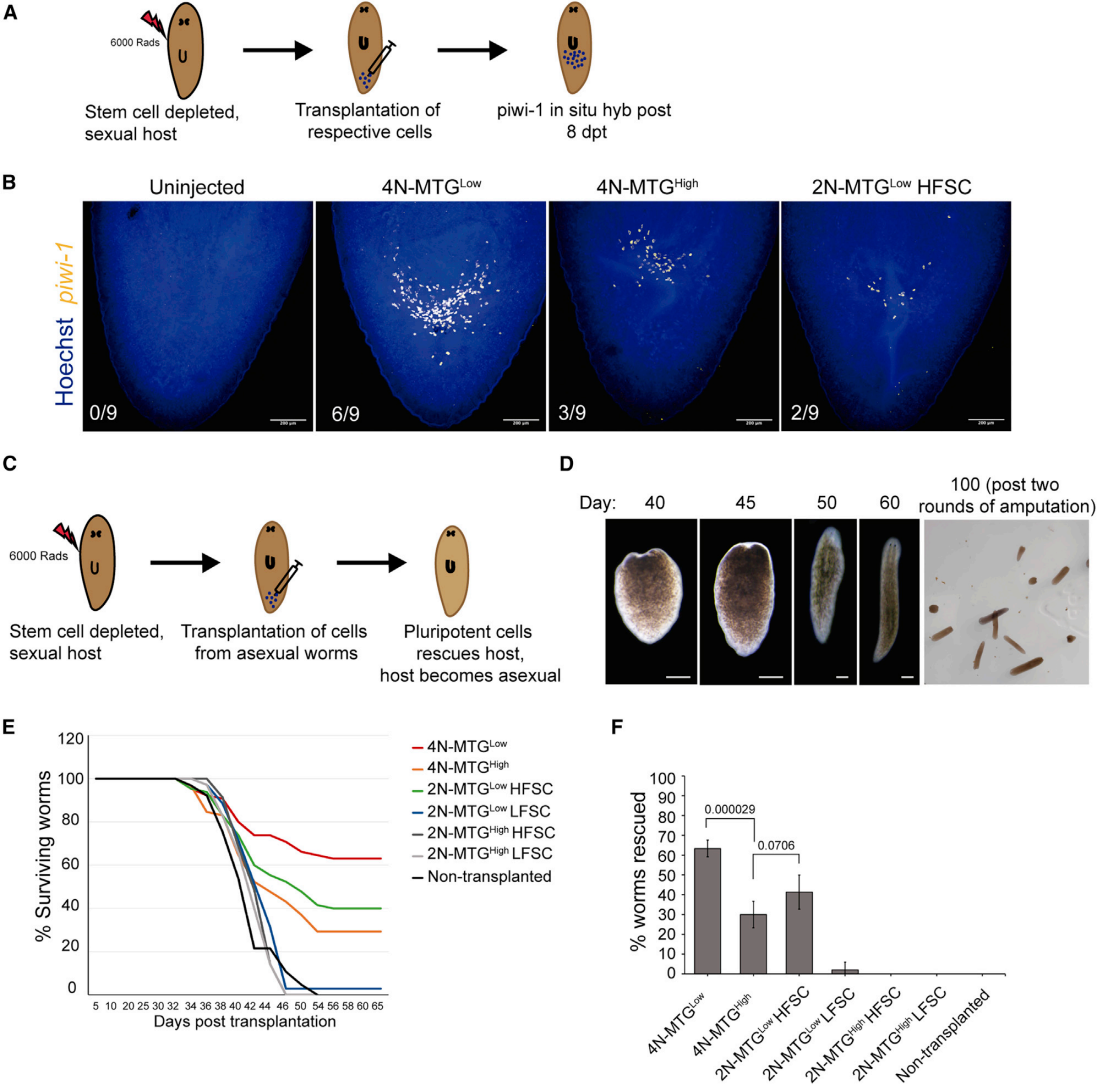
Transplantation experiment to validate the pluripotency of MTG populations (A) Schematic of colony expansion assay performed. (B) Representative *piwi-1* staining in lethally irradiated sexual worms 8 dpt of ~1000 cells from asexual, SiR-DNA stained MTG populations (see methods). Numbers indicate worms showing *piwi-1* colonies. Scale bar, 200 μm. n = 9 worms. (C) Schematic showing the long-term survival and transformation of sexuals to asexuals by clonogenic neoblast transplantation. (D) Representative images of worms surviving after receiving clonogenic neoblasts. Initially, the worms show head regression, and approximately 40 to 45 dpt, worms start to develop blastema and completely regenerate the head. After two rounds of amputation, the worms started propagating via fission. Scale bar, 500 μm. (E) Plots showing the percentage of surviving worms transplanted with ~1500 cells of the respective MTG population. n = 65 worms for 4N-MTG^Low^ and ^−High^, 2N-MTG^Low^(HFSC), uninjected control. And n = 35 for the rest of the population performed in five independent replicates. (F) Bar graph representing the average percentage of worms rescued. Error bar represents standard deviation, over five independent experiments. n = 65 worms for 4N-MTG^Low^ and ^−High^, 2N-MTG^Low^ (HFSC), and uninjected control. n = 35 for the rest of the population. Mean ± SEM, numbers on the graph represent the respective p value. See also Figure S6.

The PIWI-1 expression and the transcriptome analysis revealed that the X1 and X2-MTG^Low^-(HFSC) cells could potentially be clonogenic neoblasts compared with their MTG^High^ counterparts. This was validated by a transplantation experiment that involved the injection of the MTG populations into lethally irradiated animals. The population of cells that rescue the irradiated animals would be considered PSCs (Lei et al., 2019; Wagner et al., 2011). Recent studies have shown that injection of neoblasts from asexual into irradiated sexual planaria transformed sexual strain to asexuals (Lei et al., 2019; Wagner et al., 2011). For our transplantation experiments, we used SiR-DNA (Cytoskeleton inc.), a nuclear dye, recently used as an alternative to isolating 4N cells from planarians instead of Hoechst dye, which was shown to be toxic (Lei et al., 2019; Wagner et al., 2011; Wang et al., 2018b). First, the X1(FS) and X2(FS) gates were set using the Hoechst dye to isolate the 4N cells and 2N cells from asexual planarians. The same gating parameters were used to separate the SiR-DNA 4N cells and SiR-DNA 2N cells to reduce the cross-contamination between X1, X2, and Xins cells. The SiR-DNA cells were stained with MTG to demarcate the MTG^Low^ and MTG^High^ cells within the X1 and X2 population. To understand the PSC activity of the MTG populations, we performed *piwi-1* colony expansion assay and animal survival studies wherein the six different populations of the sorted cells from the asexual strain were transplanted into an irradiated sexual strain. Animals that were not transplanted were used as the negative control. The animals after 8 days posttransplantation (dpt) were fixed and assayed for the presence of *piwi-1*^+^ colonies (Figure 5A). We found that 4N-MTG^Low^ cells (X1-MTG^Low^ equivalent) had the maximum number of *piwi-1*^*+*^ colonies compared with the 4N-MTG^High^ (Figure 5B). Similarly, among the 2N cells (X2 equivalent), only the 2N-MTG^Low^ (HFSC) cells showed a greater number of *piwi-1*^*+*^ colony expansion (Figure 5B). None of the other three populations (X2-MTG^Low^-LFSC, X2-MTG^High^ High, and low FSC cells) showed any *piwi-1*^*+*^ colonies. We also performed survival studies in the transplanted and nontransplanted animals post lethal dose of irradiation. Typically, the nontransplanted sexual animals, post-irradiation, show head regression, ventral curling, and eventually lysed after 30 dpt. However, the sexual animals rescued with asexual neoblast eventually show fission, which is characteristic of asexual animals (Figures 5C and 5D). This result indicates that the transplanted cells have divided and replaced all the cells in the sexual (Lei et al., 2019; Wagner et al., 2011). Within the 4N cells (X1 equivalent), the MTG^Low^ population showed twice the rescue efficiency (63.33%) compared with 4N-MTG^High^ (30%) (Figures 5E and 5F). These results conclusively show that the neoblasts with low MTG are more pluripotent than the neoblasts with high MTG. Interestingly, the 2N-MTG^Low^-(HFSC) cells, showed 41.33% rescue efficiency compared with 2N-MTG^Low^ (LFSC) cells (2%, 1 of 35 animals were rescued). As expected, both the 2N-MTG^High^ (small and large cells) failed to rescue the animals (Figures 5E and 5F). The new gating strategy based on mitochondrial content described in this study experimentally validates the presence of clonogenic neoblasts within an X2 equivalent gate. Moreover, despite the presence of only ∼35% PIWI-1^High^ cells, the X2-MTG^Low^-(HFSC) cells exhibited higher rescue efficiency compared with X1 MTG^High^ cells indicating a greater clonogenic potential (Figure S6). Together, our transplantation experiments show that the decreased mitochondrial mass is a hallmark of PSCs in planarians. In contrast, the increased mitochondrial mass is an indicator of stem cell differentiation.

### Increased mitochondrial potential is essential for neoblast differentiation

**Figure 6:**
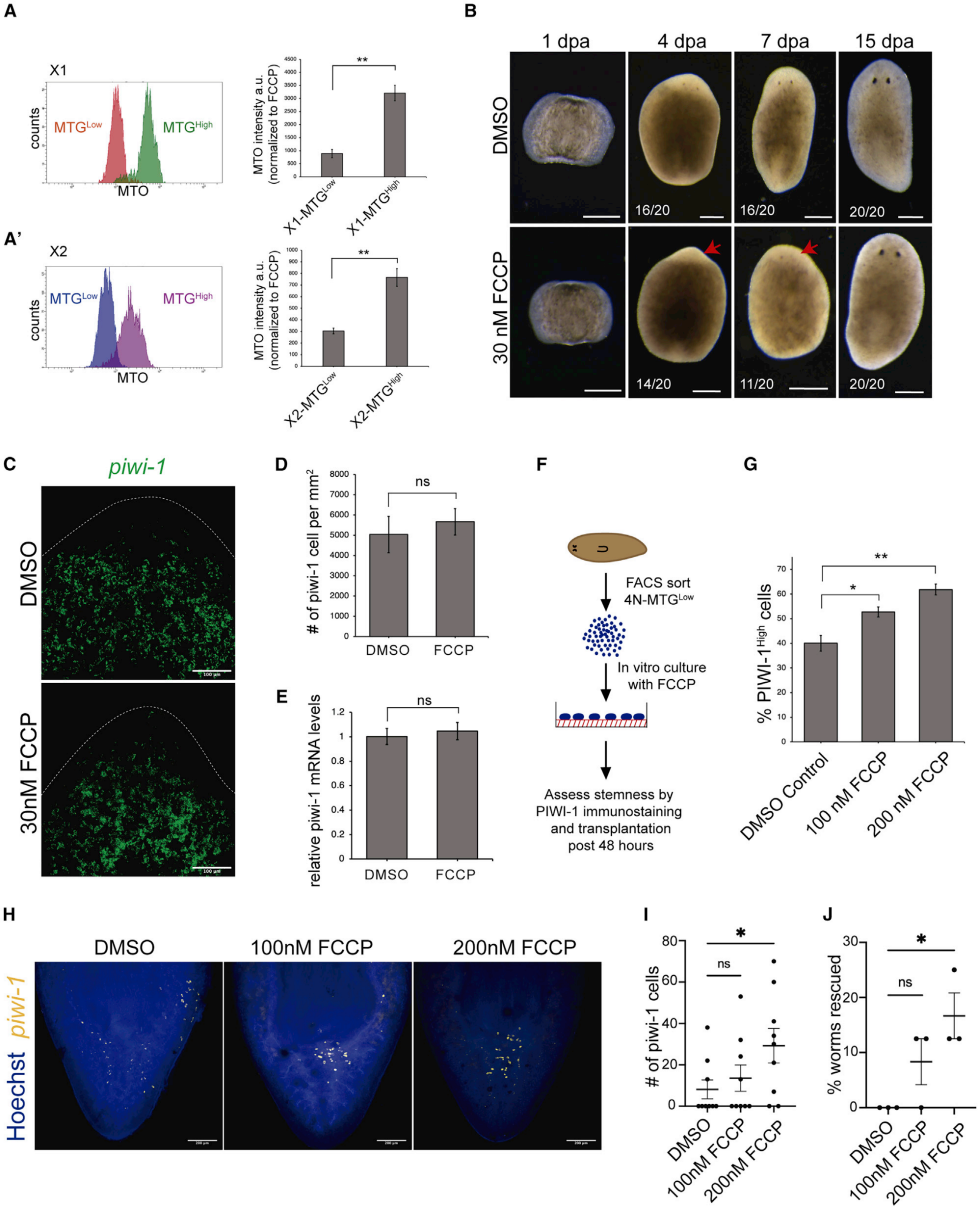
Role of mitochondria in stem cell differentiation (A and A′) Planarian cells stained with MTG and MTO were analyzed through flow cytometry. X1 (A) and X2 (A′) were gated for MTG^Low^ and ^−High^ and analyzed for MTO intensity. The bar graph indicates the median MTO intensity normalized to FCCP. Mean ± SEM, over three independent replicates. a.u., arbitrary units. (B) Representative images of undergoing regeneration in the presence of 30 nM FCCP or DMSO (vehicle control). Scale bar, 200 μm. (C) Representative confocal images of *piwi-*1 RNA FISH at 3 dpa in trunk fragments regenerating in 30 nM FCCP or DMSO (control). Scale bar, 100 μm. (D) Quantitation of *piwi-1*^+^ cells from the 3dpa anterior blastema region from (C). Mean ± SD, n = 7 animals. (E) Quantitation of *piwi-*1 mRNA levels through qPCR from 3 dpa regenerating animals. Mean ± SD, n = 30 worms over three independent replicates. (F) Schematic representing the *in vitro* assay for perturbing mitochondrial activity using FCCP. (G) PIWI-1^High^ quantitation through flow cytometry in 4N-MTG^Low^ cells treated with the indicated amount of FCCP for 48 h in culture. Mean ± SEM, over three independent replicates. (H and I) Representative *piwi-*1 RNA FISH (H) and quantification of *piwi-1*^*+*^ cells (I) per transplant in lethally irradiated sexual worms 8 dpt of ~2500 cells from 4N-MTG^Low^ cells cultured *in vitro* for 48 h with or without FCCP. Scale bar, 200 μm. Mean ± SEM, n = 9 worms. (J) Percentage of worms rescued following injection of ~2500 4N-MTG^Low^ cells cultured *in vitro* for 48 h with or without FCCP. Mean ± SEM, n = 24 worms performed over three independent replicates. ^∗^, ^∗∗^, n.s. represents p < 0.05, p < 0.01, and nonsignificant, respectively.

To understand the mitochondrial activity of MTG^Low^ and ^−High^ cells, planarian cells were simultaneously stained with MTG and MitoTracker Orange (MTO), a dye that indicates mitochondrial membrane potential (Figure S7A). It was observed that MTG^Low^ cells were also low in MTO and conversely MTG^High^ cells had high MTO intensity (Figures 6A and A′). A similar trend was also observed upon normalization of the MTO intensity to the cells treated with FCCP, an uncoupler of oxidative phosphorylation (Figures 6A and A′). This clearly indicates that the mitochondrial content correlates with mitochondrial activity. Next, we investigated the implication of changes in mitochondrial activity on stem cell differentiation. We treated the regenerating worms with FCCP. We chose low doses of FCCP (30 nM) to treat the regenerating animals, as the concentrations above 50 nM were toxic to the animals and lyse after 12 to 16 h of treatment. The worms treated with FCCP showed a 24 to 48-h delay in regeneration as revealed by the appearance of the eyespots (Figure 6B). RNA FISH and qPCR analysis for *piwi-1* showed no significant change in transcript levels between control and FCCP-treated worms (Figures 6C, 6D, and 6E). This indicates that the delay in regeneration could be attributed to FCCP hampering the differentiation process rather the affecting the viability of the stem cells. However, it is difficult to make definitive conclusions because the low doses of FCCP might be suboptimal and the reduction of mitochondrial activity *in vivo* is global and not restricted to stem cells. To specifically perturb the mitochondrial activity in stem cells, we cultured the neoblasts and treated them with FCCP (Figure 6F). Toward this, we used SiR-DNA (Cytoskeleton inc.) and sorted 4N cells which were also MTG^Low^ (X1-MTG^Low^ equivalent) and cultured as described by (Lei et al., 2019). The current cell culture system for neoblast is not completely optimized and it is observed that with time the cells differentiate and beyond 48 h lose their clonogenic potential (Lei et al., 2019). We observed that post 48 h of culture, there was a higher number of PIWI-1^High^ cells in FCCP-treated cells compared with the DMSO control (Figure 6G). Such an increase in PIWI-1^High^ cells was not a consequence of accelerated cell death as the viability of both High and Low MTG populations does not change upon treatment with 200 nM FCCP *in vitro* (Figures S7B and S7C). Transplantation assays were performed to functionally validate the stemness of the FCCP-treated neoblast. The results indicate that cells treated with 200 nM FCCP showed higher colony expansion than the control cells (Figures 6H and 6I). We then performed the rescue experiments, which revealed that 200 nM FCCP-treated cells showed ∼16.67% rescue efficiency (Figure 6J), while the DMSO control cells failed to rescue any animals. Together, these results show that the cells that were treated with FCCP *in vitro* resisted differentiation, and were maintained in the pluripotent state compared with the untreated control cells. Collectively, these results demonstrate that the differences in the mitochondrial content in the stem versus differentiated populations correlate with the mitochondrial activity and also suggest that an increased mitochondrial activity could be a requisite for neoblast differentiation.

## Discussion

Planarians are highly regenerative animals that harbor PSCs, which differentiate to lineage primed progenitors thereby controlling the extent of regeneration and tissue turnover during homeostasis (Reddien, 2018). The presence of PSCs in the adult animal makes planaria an excellent model system to study stem cell dynamics. Earlier studies using electron microscopy had shown irregular mitochondrial morphology in the neoblast and most of the mitochondria were located toward the periphery of the nuclear membrane (Hay and Coward, 1975; Morita et al., 1969; Pedersen, 1959). Also, previous studies have shown an increase in the ROS (reactive oxygen species) activity during the planarian regeneration and it was critical for the differentiation of stem cells (Pirotte et al., 2015). This study was corroborated by the work in *Xenopus* and zebrafish, which showed increased ROS activity during their tail regeneration (Gauron et al., 2013; Love et al., 2013). Inhibiting the ROS activity using the NADH oxidase inhibitors abrogated the stem cell differentiation and thus blocked regeneration implicating mitochondria as the major source of ROS generation. The primary aim of this study was to delineate the changes in mitochondrial content in the pluripotent and the committed stem cell states in planarians, which can subsequently be used as a marker to isolate PSCs.

Neoblast in planarians is characterized by the presence of a large nucleus and scanty cytoplasm. Using Hoechst, a nuclear dye, planarian cells are sorted into three classes, X1, X2, and Xins. Here, we investigated the extent of distribution of mitochondrial mass among these three populations using MTG, a mitochondrial dye. MTG accumulates in the mitochondrial matrix and covalently binds to the proteins by reacting with the free thiol groups of the cysteine residues. Its localization to the mitochondria happens regardless of the mitochondrial membrane potential and it has been used to measure the mitochondrial mass. Using MTG, we observed a low mitochondrial mass in the X1 cells compared with the Xins cells. Interestingly, MTG staining revealed a polarized distribution of mitochondria in X1 cells. It is worth noting that such subcellular localization of mitochondria has been previously implicated in cell migration and localized metabolite concentration (Alshaabi et al., 2020), which needs to be further studied in the context of the neoblast function. MTG staining further demarcated X1 and X2 populations into MTG^Low^ and MTG^High^ subpopulations. We observed that PSCs in planaria are defined by low mitochondrial content, whereas an increase in mitochondrial content is associated with differentiation. For instance, our results indicate that the stem cells in the X1 population, which had high mitochondrial content, exhibited properties of lineage commitment such as reduced PIWI-1^High^ cells and expression of transcripts associated with lineage specification. This suggests that a quantifiable change in mitochondrial mass exists within clonogenic and specialized neoblasts.

We also looked at the mitochondrial content in the X2 population, which is a mix of neoblasts and their mitotic progenies. Owing to the heterogeneity of the X2 population and low prevalence, stem cells in this gate were poorly studied. It was proposed that the clonogenic neoblasts exist in the X2 gate, nonetheless it was never experimentally validated (Molinaro and Pearson, 2018). We categorized X2 cells based on MTG intensity and characterized the subpopulations. RNA-seq analysis showed that the transcript profile of MTG^Low^ resembled stem cells and early progenitors and was reminiscent of X1 cells, while MTG^High^ was more akin to Xins. This indicated that within X2, mitochondrial content increases as the cell progresses toward differentiation. To enrich the neoblasts within the G0/G1 phase, MTG^Low^ cells in X2 were divided into two populations based on their size. Such classification of the X2 cells based on the size and MTG resulted in an enrichment of PIWI-1^High^ cells in the High FSC gate. Transcriptome sequencing revealed that X2-MTG^Low^-(LFSC) are potentially early progenitors and the X2-MTG^Low^-(HFSC) express transcripts critical for the cell cycle progression. Together, using the size parameter and mitochondrial mass measurements, we were able to isolate neoblast and their immediate progenitors for functional characterization.

Furthermore, we also used MTO, an indicator of mitochondrial potential, to study the correlation between mitochondrial content and potential. Dual staining using MTG and MTO showed that the cells with low MTG were also low for MTO and vice versa, suggesting that the mitochondrial content and potential show a high degree of correlation. It is also important to note that high mitochondrial activity leads to an increased ROS production. Recent studies have shown that MTG could be influenced by ROS (Doherty and Perl, 2017), indicating that the MTG^High^ cells could show increased ROS. Notably, increased ROS is also associated with stem cell differentiation in vertebrates (Tothova et al., 2007) and invertebrates (Owusu-Ansah and Banerjee, 2009). However, a comprehensive study is warranted to understand the effect of high mitochondrial activity and subsequent increase in the ROS levels on neoblast maintenance and differentiation.

*Schmidtea mediterranea* exists as two strains: sexual strain, which are hermaphrodites, and asexual strain, which have underdeveloped germline tissue and undergoes reproduction by fission. Previous studies have shown that the transplantation of asexual neoblast to irradiated sexual strain transformed the sexual planaria to asexual evident from them undergoing fission (Wagner et al., 2011). Such transplantation assays in planarians provide a platform to functionally validate the pluripotency of neoblast subpopulations. Our results clearly indicate that stem cells with low mitochondrial content are more pluripotent than the cells with high mitochondrial content. This implies that low mitochondrial content is one of the characteristics of PSCs. Interestingly, our transplantation experiments also revealed the higher clonogenic capacity of the G0/G1 neoblasts. With the current state-of-the-art technologies, TSPAN-1+ cells fairs as the most potent clonogenic population. We propose that the classification of cells based on mitochondrial content would allow us to sort clonogenic neoblasts with higher purity and subsequent characterization might allow us to identify novel and rare clonogenic neoblasts, should they exist. Furthermore, the sorting methodology presented here using both nuclear and mitochondrial markers could be, in principle extended to other flatworms such as *Macrostomum lignano, Schistosoma mansoni,* and *Hoefstenia miamia* where, like planaria, only proliferating stem cells have been extensively studied (Gehrke et al., 2019; Grudniewska et al., 2016; Wang et al., 2018a).

Differentiation in mammalian pluripotent cells such as ESCs is shown to involve changes in the gene expression profile, remodeling of organelles, and their metabolic states (Dixon et al., 2015; Folmes et al., 2012; Gabut et al., 2020; Sampath et al., 2008). Further, it has been shown that during differentiation, ESCs exhibit increased mitochondrial fusion and a concurrent switch in their metabolic state from glycolytic to oxidative phosphorylation. Such metabolic change has been shown to be critical for the cell state transition (Chung et al., 2007; Tormos et al., 2011). For instance, treatment of ESCs and HSCs with electron transport chain uncouplers like FCCP resulted in defective differentiation and maintained their stemness as validated by their ability to form teratoma and functional blood reconstitution respectively (Mandal et al., 2011; Vannini et al., 2016; Varum et al., 2009).

Our study demonstrates a substantial increase in the mitochondrial mass of planarian cells during differentiation and a perturbation of mitochondrial activity led to increased sustenance of stem cells *in vitro*. Such neoblasts that are blocked for OxPhos *in vitro*, exhibited higher clonogenic capacity compared with the control cells. This indicates that for proper differentiation of stem cells, a concomitant increase in mitochondrial activity is a necessity. Further, these results suggest that the mitochondrial state has a central role in stem cell maintenance and differentiation in planarians and this biological process is evolutionarily conserved. Our findings also highlight the potential role of mitochondrial bioenergetics in regulating organismal regeneration. In addition, the increased mitochondrial content during differentiation might be a result of the higher demand for metabolites in cells priming for the differentiation. For instance, early-stage embryonic stem cell differentiation is marked by epigenetic changes and increased translation state, which requires increased levels of amino acids and cofactors such as alpha-ketoglutarate and acetyl CoA (Boland et al., 2014; Folmes et al., 2012). Also, the intermediates of the TCA cycle serve as the precursors of amino acid biosynthesis and also provide cofactors essential for epigenetic modifiers such as acetylases and demethylases. However, an in-depth study is required for a complete understanding of the exact processes that lead to increased mitochondrial mass during neoblast differentiation. In summary, the results presented here establish without ambiguity that the PSCs have lesser mitochondrial mass compared with their progenitors and this difference in the mitochondrial mass serves as an efficient marker to isolate pluripotent neoblast from their progenitors.

## Experimental procedures

### Planarian husbandry

Both sexual and asexual *Schmidtea mediterranea* strains were grown in 1x Montjuïc salts at 20°C. The worms were fed with beef liver and were starved for a minimum of 7 days before any experiments. Sexual strains receiving 6,000 rads of γ-rays were used as transplantation hosts. The transplantation hosts were maintained in gentamicin (50 μg/mL) starting 7 days prior to irradiation.

### Fluorescence-activated cell sorting

Cell suspension for FACS sorting was prepared as described before (Lei et al., 2019). The worms were diced in calcium and magnesium-free buffer with 1% BSA (CMFB) and mechanically sheared using a micropipette. The resulting single-cell suspension was stained with Hoechst 33,342 (40 μg/mL) and MTG (100 nM). X1, X2, and Xins populations were demarcated using Hoechst blue and red fluorescence. For transplantation experiments, cells stained with SiR-DNA and MTG were used.

### Fluorescence *in situ* hybridization and immunostaining

*In situ* hybridization for assessing the *piwi-1* colonies in transplanted worms were performed as described earlier (King and Newmark, 2013). For immunostaining, an Anti-PIWI-1 antibody was raised in rabbit using the antigen NEPEGPTETDQSLS as described earlier (Guo et al., 2006). FACS sorted cells were plated in optical bottom 384 well plates (∼10,000 cells per well) and stained with PIWI-1 antibody.

### Cell transplantation

Bulk cell transplantation in irradiated animals was carried out as described earlier with minor modifications (Davies et al., 2017; Wang et al., 2018b). For colony expansion and long-term survival experiments, 2-day post-irradiated animals were used. The injection was carried out using an Eppendorf femtojet 4x with a pressure of 0.8 to 1.0 psi.

### Statistical analysis

All statistical analyses except for transcriptome were performed using unpaired, two-tailed Student's t test and p < 0.05 was considered significant. Differentially expressed genes from the transcriptome analysis (two independent biological replicates) were identified using the Cuffdiff module and genes with adjusted p value <0.05 were considered as significant. For GO analysis, >2-fold upregulated, statistically significant genes were considered.

### Accession numbers

The accession number for the sequencing data reported in this paper is NCBI- Sequence Read Archive (SRA): SRP272800.

## Author contributions

MMH, PKV, and DP conceived and designed the study. MMH, SRS performed the experiments. VL performed the RNA-seq analysis. KL, PKV, and DP supervised the entire study. MMH and DP wrote the manuscript with inputs from all the authors. PKV and DP acquired funding.

## Supplemental information

Document S1. Supplemental experimental procedures and Figures S1–S7

Table S1. X1_MTG low vs high

Table S2. X2_MTG low vs high

Table S3. X2_MTG_low HFSC vs LFSC

Document S2. Article plus Supplemental information
