# Supplementary material for: Mitochondrial state determines functionally divergent stem cell population in planaria
Source: Stem Cell Reports. 2021 Apr 15;16(5):1302–16. doi: 10.1016/j.stemcr.2021.03.022 (PMC8185449; doi:10.1016/j.stemcr.2021.03.022)
Supplement: Document S1. Supplemental experimental procedures and Figures S1–S7 [file mmc1.pdf]

Stem Cell Reports, Volume *16*

Supplemental Information

Mitochondrial state determines functionally divergent stem cell population in planaria

Mohamed Mohamed Haroon, Vairavan Lakshmanan, Souradeep R. Sarkar, Kai Lei, Praveen Kumar Vemula, and Dasaradhi Palakodeti

Figure S1:

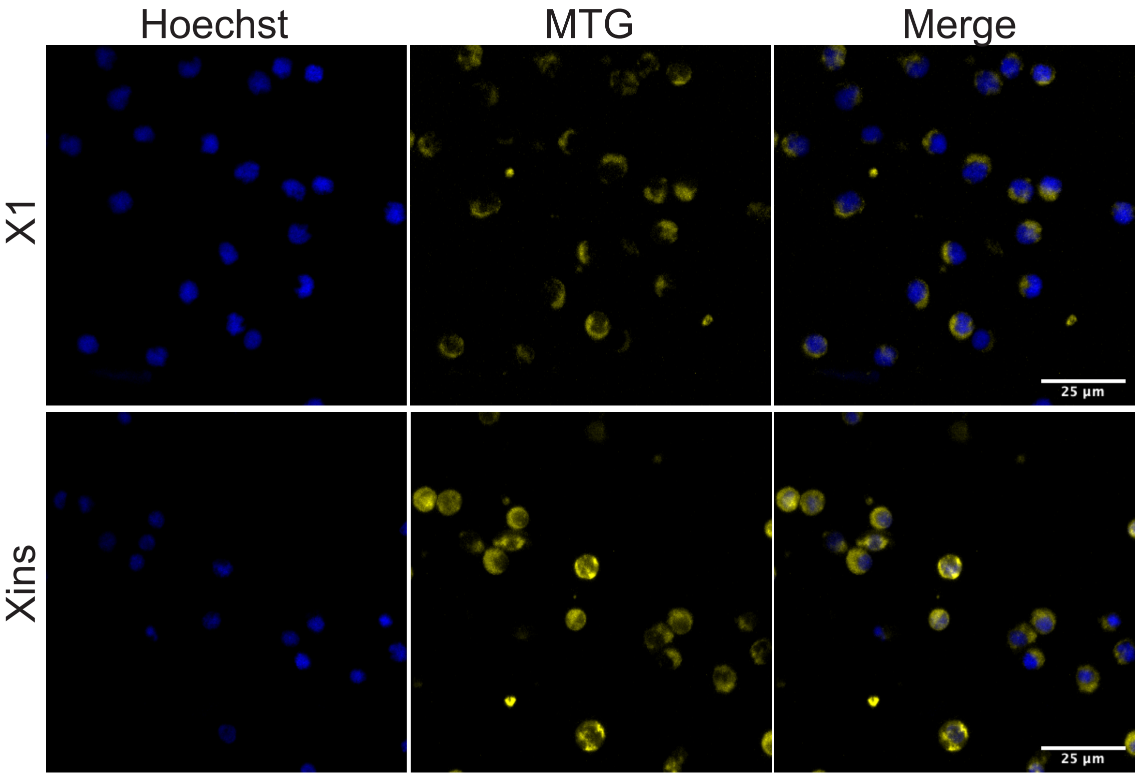

Figure S2:

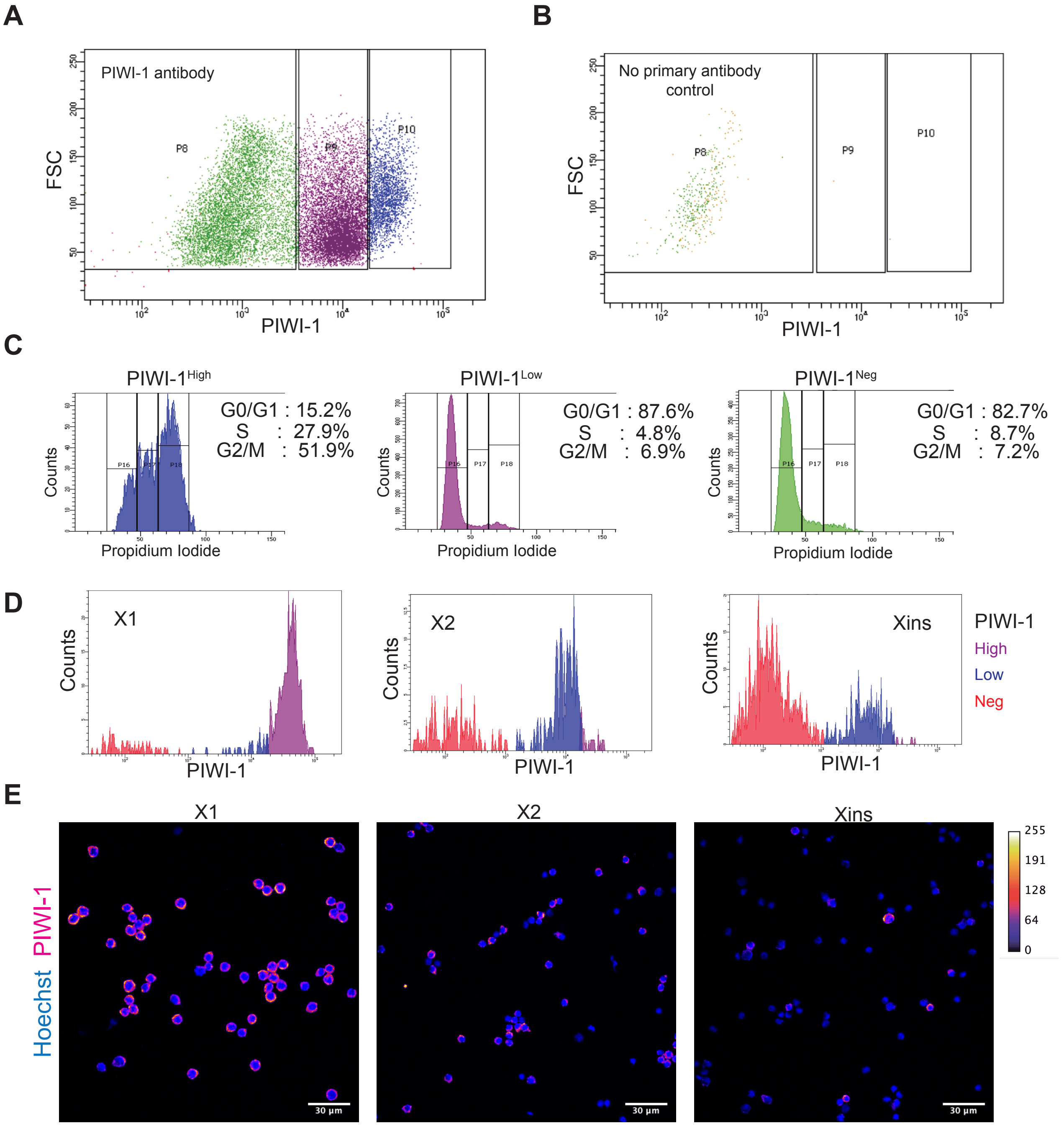
A
PIWI-1 antibody
FSC
P8
P9
P10
PIWI-1
B
No primary antibody control
FSC
P8
P9
P10
PIWI-1
C
PIWI-1High
G0/G1 : 15.2%
S : 27.9%
G2/M : 51.9%
Counts
Propidium Iodide
PIWI-1Low
G0/G1 : 87.6%
S : 4.8%
G2/M : 6.9%
Counts
Propidium Iodide
PIWI-1Neg
G0/G1 : 82.7%
S : 8.7%
G2/M : 7.2%
Counts
Propidium Iodide
P16
P17
P18
D
X1
Counts
PIWI-1
X2
Counts
PIWI-1
Xins
Counts
PIWI-1
PIWI-1
High
Low
Neg
E
X1
X2
Xins
Hoechst PIWI-1
30 µm
255
191
128
64
0

Figure S3:

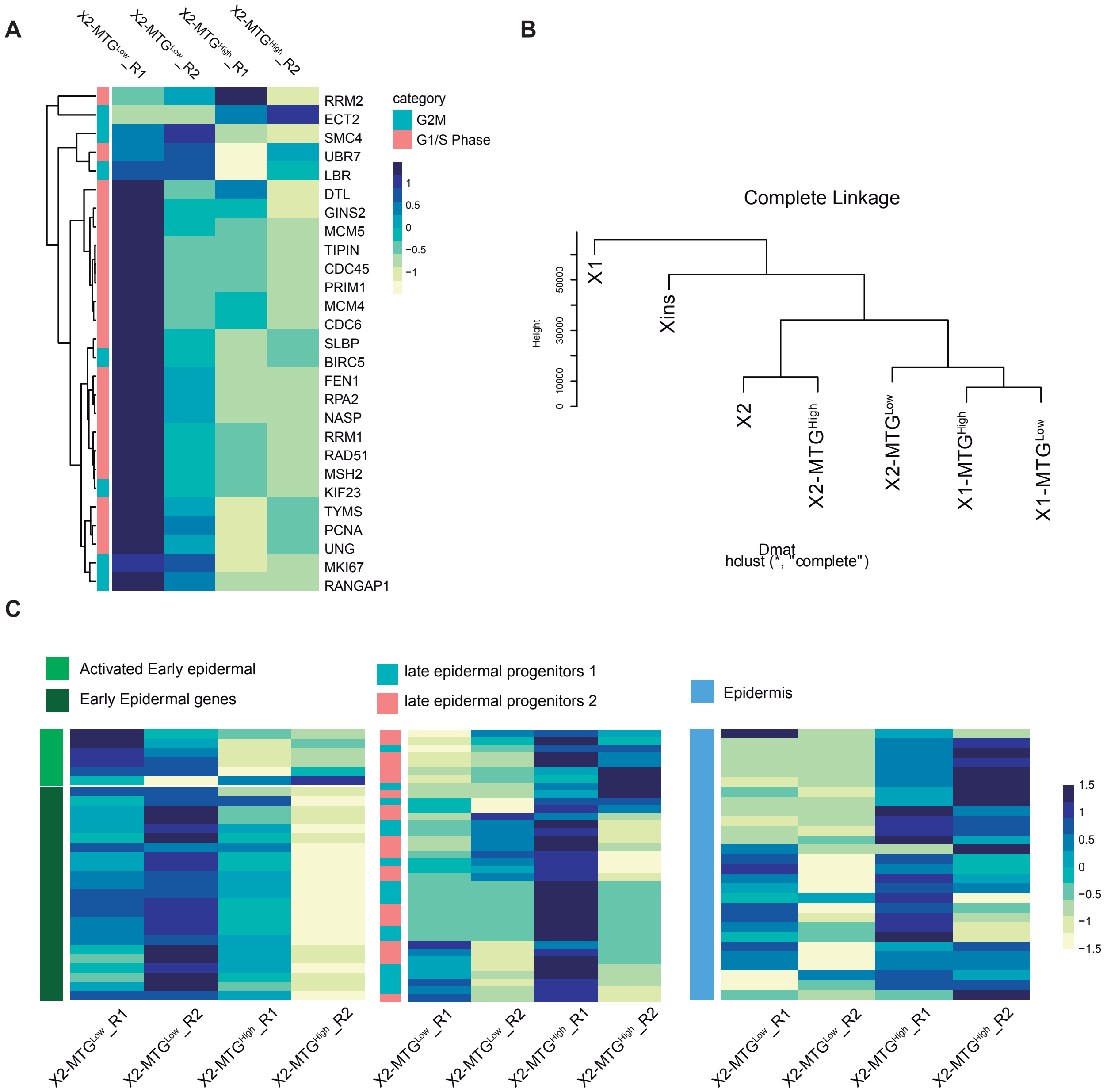
A
B
C
X2-MTGLow_R1
X2-MTGLow_R2
X2-MTGHigh_R1
X2-MTGHigh_R2
RRM2
ECT2
SMC4
UBR7
LBR
DTL
GINS2
MCM5
TIPIN
CDC45
PRIM1
MCM4
CDC6
SLBP
BIRC5
FEN1
RPA2
NASP
RRM1
RAD51
MSH2
KIF23
TYMS
PCNA
UNG
MKI67
RANGAP1
category
G2M
G1/S Phase
1
0.5
0
−0.5
−1
Complete Linkage
Height
0
10000
30000
50000
X1
Xins
X2
X2-MTGHigh
X2-MTGLow
X1-MTGHigh
X1-MTGLow
Dmat
hclust (*, "complete")
Activated Early epidermal
Early Epidermal genes
late epidermal progenitors 1
late epidermal progenitors 2
Epidermis
1.5
1
0.5
0
−0.5
−1
−1.5

Figure S4:

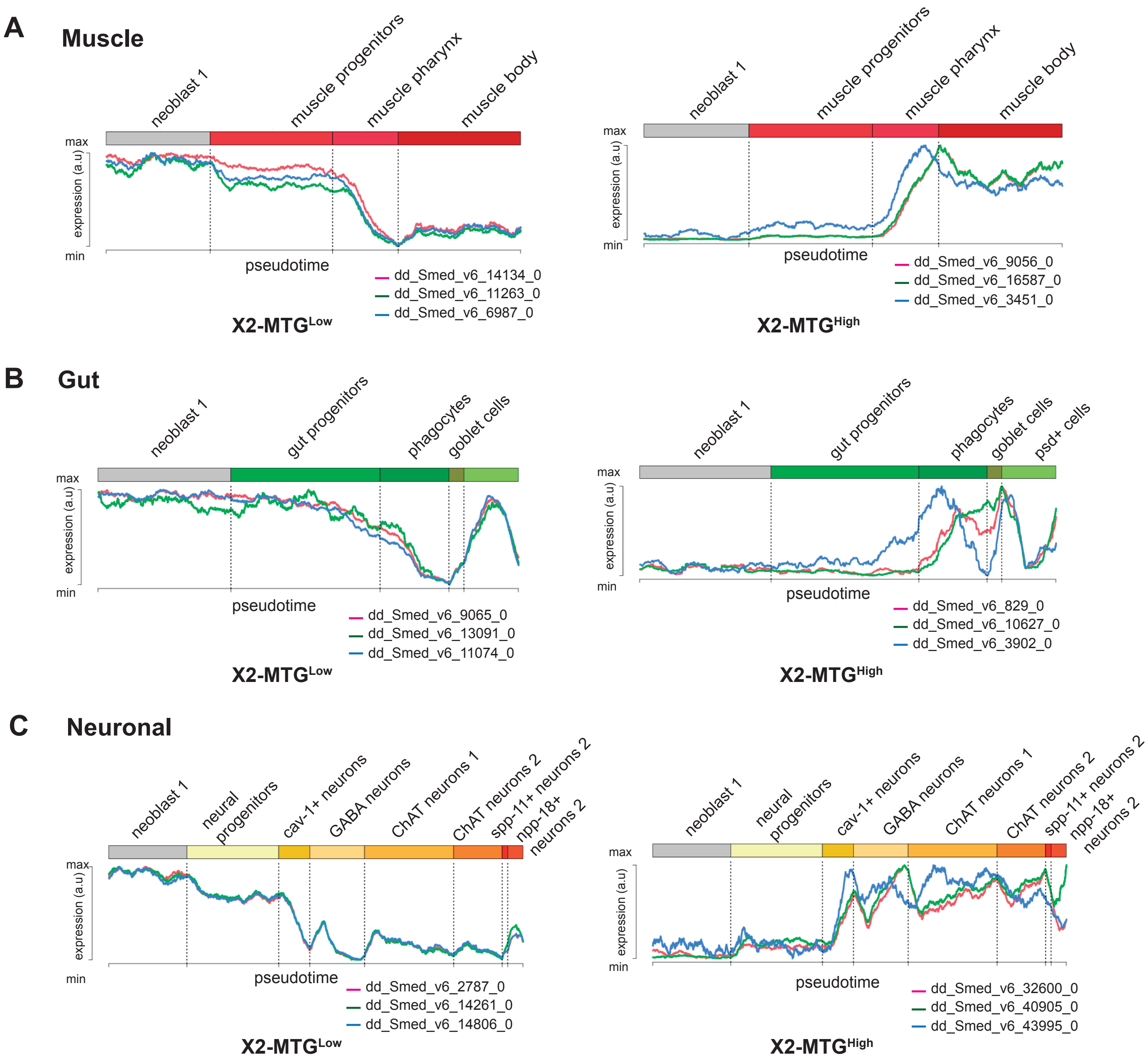
A
Muscle
neoblast 1
muscle progenitors
muscle pharynx
muscle body
max
min
expression (a.u)
pseudotime
dd_Smed_v6_14134_0
dd_Smed_v6_11263_0
dd_Smed_v6_6987_0
X2-MTG^Low
dd_Smed_v6_9056_0
dd_Smed_v6_16587_0
dd_Smed_v6_3451_0
X2-MTG^High
B
Gut
neoblast 1
gut progenitors
phagocytes
goblet cells
psd+ cells
dd_Smed_v6_9065_0
dd_Smed_v6_13091_0
dd_Smed_v6_11074_0
X2-MTG^Low
dd_Smed_v6_829_0
dd_Smed_v6_10627_0
dd_Smed_v6_3902_0
X2-MTG^High
C
Neuronal
neoblast 1
neural progenitors
cav-1+ neurons
GABA neurons
ChAT neurons 1
ChAT neurons 2
spp-11+ neurons 2
npp-18+ neurons 2
dd_Smed_v6_2787_0
dd_Smed_v6_14261_0
dd_Smed_v6_14806_0
X2-MTG^Low
dd_Smed_v6_32600_0
dd_Smed_v6_40905_0
dd_Smed_v6_43995_0
X2-MTG^High

Figure S5:

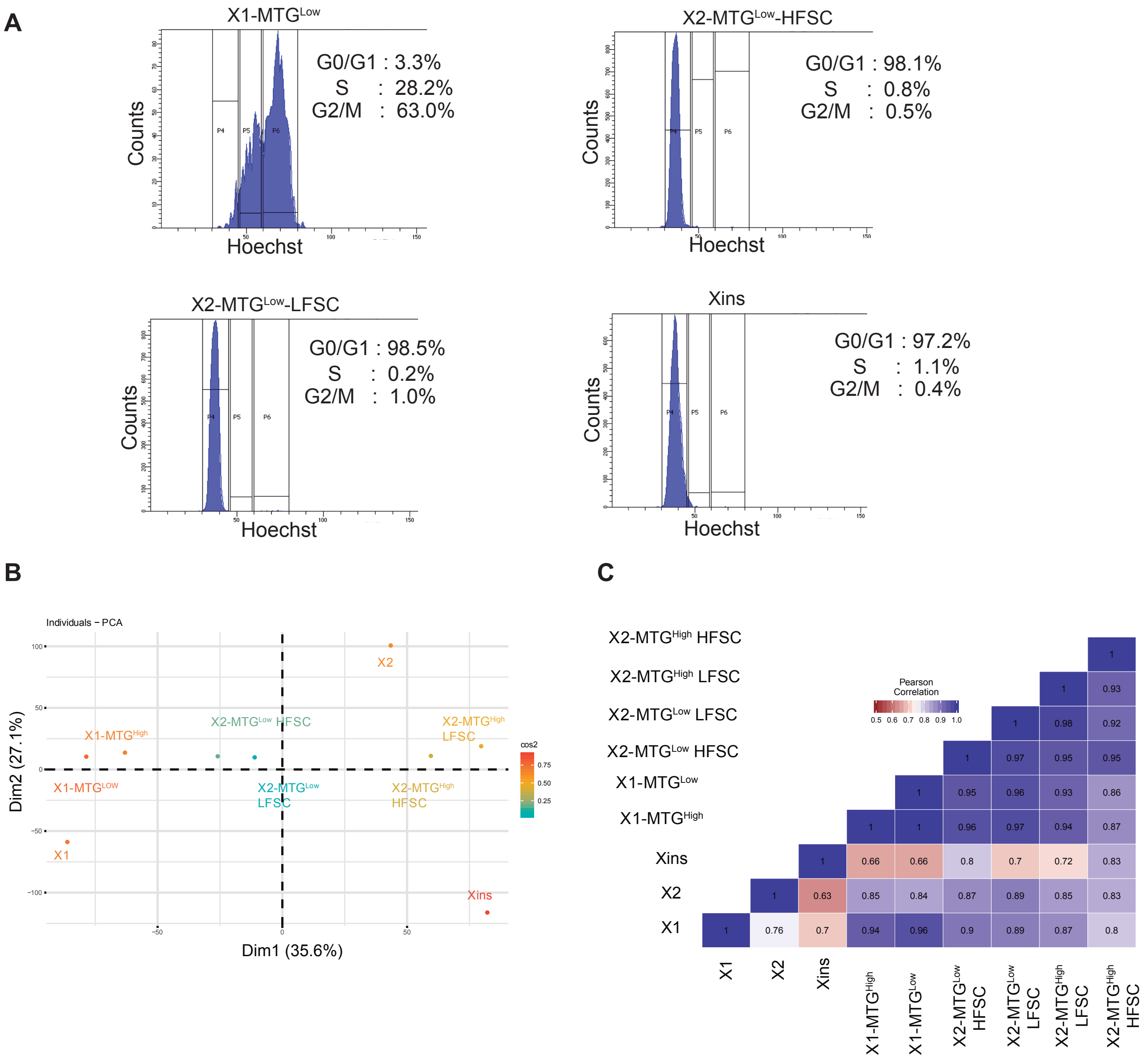
A
X1-MTG^Low
G0/G1 : 3.3%
S : 28.2%
G2/M : 63.0%
Counts
Hoechst
X2-MTG^Low-HFSC
G0/G1 : 98.1%
S : 0.8%
G2/M : 0.5%
X2-MTG^Low-LFSC
G0/G1 : 98.5%
S : 0.2%
G2/M : 1.0%
Xins
G0/G1 : 97.2%
S : 1.1%
G2/M : 0.4%
B
Individuals – PCA
Dim2 (27.1%)
Dim1 (35.6%)
X2
X1-MTG^High
X2-MTG^Low HFSC
X2-MTG^High LFSC
X1-MTG^Low
X2-MTG^Low LFSC
X2-MTG^High HFSC
X1
Xins
cos2
C
Pearson Correlation
X2-MTG^High HFSC
X2-MTG^High LFSC
X2-MTG^Low LFSC
X2-MTG^Low HFSC
X1-MTG^Low
X1-MTG^High
Xins
X2
X1

Figure S6:

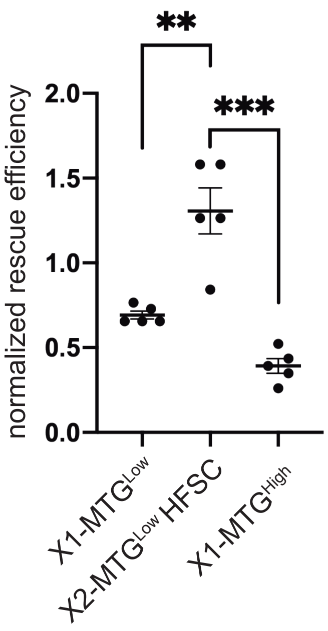

Figure S7:

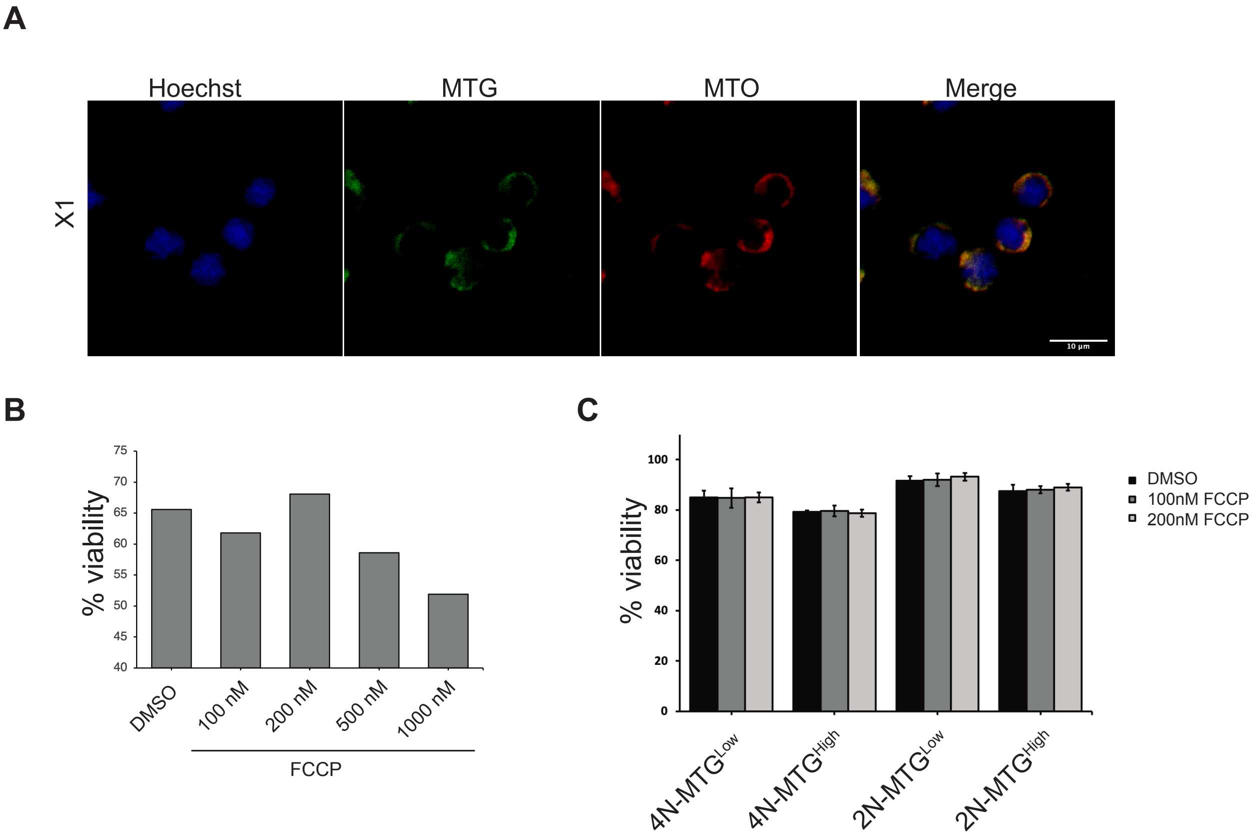

Supplemental figure legends:

Supplementary Figure S1 (related to Figure 1). Representative low magnification images of MTG fluorescence in sorted live X1 and Xins cells. Scale bar 25 μm.

Supplementary Figure S2. PIWI-1 Antibody staining validation (related to Figure 2,3)

(A) Dot plot showing PIWI-1 staining in planarian cells. PIWI-1 High (blue), low (pink), and negative (green).

(B) Dot plot of negative control cells- only secondary antibody was added without any primary antibody.

(C) Cell cycle stages of corresponding PIWI-1 population analyzed by flow cytometry. Percentage of cells in G0/G1, S, and G2/M for the indicated PIWI-1 population is given.

(D and E) PIWI-1 staining in sorted X1, X2, and Xins cells analyzed by flow cytometry **(D)** or confocal microscopy **(E)** scale bar: 30 μm. PIWI-1 signal is shown as Fire LUT (ImageJ)

Supplementary Figure S3. Transcriptome analysis of X2-MTG^{Low} and MTG^{High} population (related to Figure 4)

(A) Heat map showing the expression of key cell cycle genes in X2-MTG^{Low} and MTG^{High} cells.

(B) Linkage mapping of X2-MTG populations with X1-MTG and X1, X2, and Xins populations.

(C) Heat map showing expression of key genes implicated in early epidermal progenitors, late progenitors and epidermis in X2-MTG^{Low} and MTG^{High} cells. R1 and R2 represent biological replicates.

Supplementary Figure S4. Pseudotime analysis of transcripts X2-MTG^{Low} and MTG^{High} population (related to Figure 4)

(A-C) Representative transcripts enriched in either X2-MTG^{Low} (left) or X2-MTG^{High} (right) were used to analyze the pseudotime expression from neoblasts to terminally differentiated cells using an available single-cell database. Three representative transcripts related to the muscle **(A)**, gut **(B),** and neuronal **(C)** lineages are shown

Supplementary Figure S4. Cell cycle and transcriptome analysis of X2-MTG^{Low} High and Low FSC populations (related to Figure 4)

(A) Representative flow cytometry histograms indicating cell cycle stages of X1, Xins, and X2-MTG subpopulations which were sorted, fixed, and stained with Hoechst.

(B) Principal component analysis comparing all the MTG subpopulations with X1, X2, and Xins

(C) Pearson's correlation heatmap of the MTG populations compared to X1, X2, and Xins

Supplementary Figure S6. Normalized rescue of MTG subpopulations (related to Figure 5)

(A) Normalized rescue efficiency calculated by dividing the rescue efficiency by the percentage of PIWI-1^{High} cells obtained from Figures 2B and 3B.

Supplementary Figure S7 (related to figure 6)

(A) Representative confocal micrograph showing colocalization of MTG and MTO staining in X1 cells. Scale bar 10 μm.

(B) Cell viability obtained by propidium iodide exclusion of X1(fs) cells treated with the indicated concentration of FCCP or DMSO for 48 hours *in vitro*. Concentrations above 500 nM FCCP were significantly toxic.

(C) Cell viability of indicated X1 and X2 MTG subpopulations after 48 hours of *in vitro* culture in either 100nM or 200nM FCCP. Error bar indicated standard deviation from three independent replicates.

Supplemental experimental procedures

FACS sorting of cells using Hoechst 33342 and SiR-DNA

Cell suspension was prepared as described before (Lei et al., 2019). In brief, planarians were chopped in calcium and magnesium-free buffer (Wang et al., 2018) with 1% bovine serum albumin (CMFB) (Sigma BSA A2153) and transferred to a 50 mL centrifuge tube using a wide-bore pipette tip. The diced fragments are mechanically sheared by pipetting until all the fragments are dissociated. The resulting cell suspension is strained through a 40 μm cell strainer (BD Falcon, 352340) and centrifuged at 290 g for 10 mins. Then, the supernatant was removed and isotonic planaria media (IPM) (Lei et al., 2019) with 10% FBS is added. To this solution, 40 μg/mL Hoechst 33342 (Invitrogen, H3570) was added and the staining is carried out at room temperature (RT) for 50 mins with intermittent mixing. To the same suspension, MitoTracker Green FM (Invitrogen, M7514) was added to a final concentration of 100 nM and stained for further 20 mins. Post staining, the suspension was again centrifuged (290 g, 10 mins) and the supernatant was discarded. The resulting cells were resuspended in IPM 10% FBS with 1 μg/mL propidium iodide (Sigma, P4864) and immediately analyzed through flow cytometry. A similar protocol was used for SiR-DNA (1 μM, Cytoskeleton, Inc. CY-SC007) staining, except for propidium iodide, DAPI (1 mg/mL) (Sigma D9542) was used to discriminate live/dead cells. X1 and X2 gates were set as reported previously (Zeng et al., 2018). A wider gate for X2 was preferred in order to accommodate late progenitors. For size-based sorting of MTG populations, forward scatter voltage was adjusted such that X1 and X2 populations are well separated in the FSC axis. First, Hoechst stained cells were used to define the forward and side scatter for X1(FS) and X2(FS) gates. Within the X1(FS) gate 4N SiR-DNA population was selected (X1 equivalent) and within the X2(FS) gate 2N cells were

selected (X2 equivalent) in order to reduce cross-contamination. For Verapamil (Cytoskeleton, Inc. CY-SC007) treatment, the planarian cell suspension was treated with Hoechst and MTG in the presence of indicated concentrations of Verapamil for 1 hour. The cells were sorted using 100 μm tip and 20 psi sheath pressure in BD FACS ARIA III or Aria Fusion sorter.

Quantitation of mitochondrial membrane potential

Planarian single-cell suspensions prepared as described previously was treated with Hoechst for 40 mins and simultaneously stained with MTG and MTO (100 nM each) for another 20 mins and analyzed through flow cytometry (BD FACS Fortessa). As a control, cells were also treated with 20 μM of FCCP for 10 mins prior to the addition of MTO. Median MTO intensity of FCCP treated cells was subtracted from the non-FCCP treated population to get the true membrane potential values.

Antibody staining of FACS sorted cells

Anti-PIWI-1 antibody was raised in rabbit using the antigen NEPEGPTETDQSLS as described earlier (Guo et al., 2006). FACS sorted cells were centrifuged (300 g, 10 mins) and resuspended in IPM 5% FBS and 50 μL (~10,000 cells) was added in 384 well plates (Thermo Scientific Nunc, 142761). The plates were centrifuged and the cells were fixed in 50 μL, 4% PFA in PBSTx (0.1% Triton X100) for 10 minutes. Post incubation, 50 μL was removed, and again 50 μL of fresh PFA fixative was added and incubated for further 10 mins. Then, the entire solution was carefully removed and washed with PBSTx and permeabilized with ice-cold 90% methanol for 10 minutes. After washing twice with PBSTx, blocking was carried out for 30 minutes at RT with 10% horse serum in PBSTx. Anti-PIWI-1 antibody was diluted to 1:300 in blocking buffer was added and incubated overnight at 4 degrees Celsius. After washing with PBSTx, secondary antibody was added (1:1000) (Anti-rabbit IgG-Alexa Fluor 680, Invitrogen, A10043) for one hour at RT. The cells were further washed and stained with Hoechst.

Cell cycle analysis:

Freshly sorted cells were centrifuged and resuspended in fixative (4% PFA in PBSTx) for 20 minutes at RT. The cells were washed twice with PBSTx and stained with Hoechst 33342 for 2 hours at RT with intermittent mixing and analyzed through flow cytometry.

Neoblast culture *in vitro*

Culturing of neoblast cells was performed as described previously (Lei et al., 2019). Briefly, ~120,000 FACS sorted 4N-MTG^{Low} cells were centrifuged and resuspended in KnockOut™ DMEM (Thermo Scientific 10829018) with 5% fetal bovine serum and plated in 24 well plate pretreated with Poly-D-Lysine (50 µg/mL, BD Biosciences, 354210). The media was supplemented with the indicated concentration of FCCP or vehicle alone (DMSO) and cultured for 48 hours at 22 degrees Celsius and 5% CO_2. Post 48 hours, PIWI-1 antibody staining was performed as described earlier.

Cell viability assay:

Propidium iodide exclusion assay was performed in order to assess the viability of cultured cells. First, X1(fs) cells were sorted and culture as described above in the presence of either DMSO (vehicle control) or various FCCP concentrations (100nM- 1000nM). After 48 hours, cells were washed and media containing propidium iodide (2 µg/mL) was added. The cells were immediately analyzed through flow cytometry. The concentrations 100 and 200 nM which were found not to be toxic were used for further experiments. The viability of various MTG populations upon treatment with 100 and 200 nM was assessed separately as described above.

FCCP treatment in regenerating planarians:

For FCCP treatment *in vivo*, planarians were amputated and ~4 hours post-amputation, the trunk fragments were treated with FCCP or DMSO in 1x Montjuïc salt (vehicle control). A fresh solution of FCCP or DMSO was replaced every day.

Fluorescent in situ hybridization

In situ hybridization for assessing the *piwi-1* colonies in transplanted worms and regenerating worms treated with FCCP were performed as described earlier with minor modifications (King and Newmark, 2013). RNA riboprobe labelled with Digoxigenin against *piwi-1* was prepared by DIG RNA Labeling Mix (Sigma, 11277073910). 8 days post-transplantation, worms were killed in 5% n-acetyl cysteine in PBS and fixed in 4% formaldehyde. The worms were serially dehydrated in methanol and stored at -20 degrees overnight. The worms were then rehydrated and bleached by H_2O_2 in formamide under white light. The bleached worms were permeabilized using Proteinase K (2 µg/mL), followed by fixation using 4% formaldehyde in PBSTx (0.5% Triton X-100). The worms were then transferred to prehybridization solution for 2 hours at 56 deg. Following this, hybridization was performed for 18-20 hours at 56 deg. Post hybridization, the worms were washed with 2X and 0.2X SSC buffer, followed by TNTx (0.1 M tris, 0.15 M NaCl, 0.3% Triton X-100, pH 7.5). The worms were then blocked in 5% horse serum, 0.5% Roche western blocking reagent in TNTx. Anti-digoxigenin POD antibody (1:1000) was added and incubated overnight at 4 degrees. The worms were then washed and developed using CY-3 tyramide signal amplification. The worms were counterstained with Hoechst and mounted using Scale A2.

Microscopy

Olympus FV3000 and FV 1000 confocal microscopes were used to image *piwi-1* FISH and immunostaining. The images were analyzed using imageJ (https://imagej.nih.gov/ij).

Micrographs of live worms recovering from irradiation experiments and FCCP treated regenerating worms were acquired using Olympus SZ-16 stereo microscope.

RNA extraction

Total RNA was extracted from sorted cells and FCCP treated worms using Trizol™ method (Invitrogen, 15596026). Cells were centrifuged and 0.5 mL of Trizol™ was added for ~100,000 cells. For regenerating 3dpa animals, 10 animals were dissolved in 1 mL of Trizol™. RNA extraction was carried out as per the manufacturer's protocol. Total RNA extracted was quantified using Qubit RNA HS assay kit (Invitrogen Q32852) method and the RNA quality and integrity were verified using Bioanalyzer.

Transcriptomic profiling of mitochondrial populations

Transcriptome profiling for 6 population (X1-MTG^{Low}, X1-MTG^{High}, X2-MTG^{Low} HFSC and LFSC cells, X2-MTG^{High} HFSC and LFSC cells) were performed. Transcriptome library was prepared using NEBNext® Ultra™ II Directional RNA Library Prep with Sample Purification Beads (Catalog no-E7765L) kit and sequenced in Illumina HiSeq 2500 machine. All the samples were sequenced (as single-end) in biological replicates. Approximately 25 to 40 million reads were sequenced for every sample. These reads were adapter trimmed using Trimmomatic (Bolger et al., 2014) and mapped to rRNA and other contamination databases. Reads that did not align with these databases were taken for further analysis. We used reference-based transcriptome assembly algorithms Hisat2 v2.1.0 (Kim et al., 2015); Cufflinks v2.2.1 (Trapnell et al., 2010) and Cuffdiff v2.2.1 (Trapnell et al., 2013) to identify differentially expressed transcripts. We used Hisat2 (-q -p 8 --min-intronlen 50 --max-intronlen 250000 --dta-cufflinks --new-summary --summary-file) to align the reads back to dd_Smes_G4 (Grohme et al., 2018) assembly of *Schmidtea mediterranea* genome. Around 52-70% of reads were mapped to dd_Smes_G4 genome. We used samtools to obtain sorted bam files. The mapped reads were assembled using Cufflinks (-p -o -b -u -N --total-hits-norm -G)

with the most recent and well-annotated SMEST transcriptome as reference (http://planmine.mpi-cbg.de/planmine/assemblyReport.do?assemblyId=SMEST.1_report.html). We used cuffmerge to merge the gene list across different conditions. We identified differentially expressed genes using Cuffdiff module (-p -o ./ -b -u -N --total-hits-norm -L) and considered genes with adjusted p-value <0.05 as significance cut-off. Genes with significant p-value and at least two-fold up/downregulation were considered for GO and pathway analysis. Along with these mitochondrial based sub-populations we have used RNASeq data for the total X1, X2 and Xins population from SRA. We downloaded X1 and Xins transcriptome data from PRJNA296017 (four replicates each) (Tu et al., 2015) and X2 from PRJNA167022 (two replicates) (Labbé et al., 2012). We analysed these data as described above and used them for dimensional reduction analysis (PCA, clustering). Like X1 cells, to obtain only MTG low and high populations for X2, we considered HFSC and LFSC cells of the same MTG levels (either low or high) as replicates.

One of the replicates for the X2 MTG High HFSC replicate showed poor correlation to the genome, so we have removed that one replicate from the analysis.

To infer enriched biological pathways or processes that are regulated in these subpopulations, we blasted (using blastp, blastx) transcripts that are significantly up/downregulated ($p<0.05$, $\geq$ 2fold) against Swissprot, Uniref90, and complete uniport database to identify homolog sequence ids. These ids were used as input for pathway & gene-ontology analysis. Corresponding blast annotations are mentioned in all the supplementary tables. We did pathway analysis & gene-ontology analysis for these up/down regulated transcripts using GSEA (Mootha et al., 2003; Subramanian et al., 2005). We used a customized perl script for all the analysis used in this study. We used R ggplot2 (Wickham, 2009), pHeatmap, and CummeRbund (Goff et al., 2012) library for plotting. Different planarian cell-type markers were obtained from the available single-cell transcriptome data (Fincher et al.,

2018; Plass et al., 2018; Van Wolfswinkel et al., 2014). Expression of these markers (FPKM values) from all the different cell populations is plotted as heatmaps using pHeatmap (https://cran.r-project.org/web/packages/pheatmap/index.html). Pseudotime analysis was performed with representative transcripts from the different populations using an available single-cell dataset (https://shiny.mdc-berlin.de/psca/).

Quantitative Real-Time PCR

Animals treated with either FCCP or DMSO (vehicle-control) at 3dpa were used for quantitating piwi-1 transcript levels. RNA was isolated using Trizol™ method as described above. 1 μg of the total was used to synthesize cDNA using PrimeScript 1st strand cDNA Synthesis Kit (Takara, Cat # 6110A) and Oligo dT primers. qPCR was performed using TB Green Premix Ex Taq II (Tli RNase H Plus) (Takara, Cat #RR820B) in Applied Biosystems. Data were normalized to Actin transcript levels and analysed by ΔΔCT method.

The following primers were used in the study.

Piwi-1 forward: ACCGTATTGAAACGTGAGCC

Piwi-1 reverse: TACACATCCCCCAGCTCTTC

Actin forward: GCTCCACTCAATCCAAAAGC

Actin reverse: TCAAATCTCTACCGGCCAAG

Bulk cell transplantation

Bulk cell transplantation in irradiated animals was carried out as described earlier with minor modifications (Davies et al., 2017; Wang et al., 2018). FACS sorted cells were centrifuged and resuspended in IPM 5% FBS media and kept in ice throughout the experiments. For colony expansion and long term survival experiments, 2 days post irradiated animals were used. The irradiated worm was placed ventral side up above a black filter paper placed in a cold plate. The injection was carried out using an Eppendorf femtojet 4x with a pressure of 0.8-

1.0 psi. Glass capillaries (length 3.5”, ID: 0.53 mm, OD: 1.14 mm, Drummond Scientific, Inc. USA) were pulled using Sutter Instrument model P-1000. Cell suspensions were loaded onto the pulled capillaries using a mouth pipette (Sigma, A5177-5EA). For colony expansion assay, ~1000 cells/µL were injected and for long-term survival assay, ~1500 cells/µL was injected into the post gonopore midline of sexual hosts. For transplantation after in vitro culture ~2500 cells were injected per transplant. The transplant hosts and the uninjected control worms were maintained in gentamicin 50 µg/mL in 6 well plates with planaria water changes every two days. After 60 days, the surviving worms were fed with beef liver and amputated to regenerate twice. The fission activity of the rescued worms was monitored.

References

Bolger, A.M., Lohse, M., and Usadel, B. (2014). Trimmomatic: A flexible trimmer for Illumina sequence data. Bioinformatics *30*, 2114-212-.

Davies, E.L., Lei, K., Seidel, C.W., Kroesen, A.E., Mckinney, S.A., Guo, L., Robb, S.M.C., Ross, E.J., Gotting, K., and Sa, A. (2017). Embryonic origin of adult stem cells required for tissue homeostasis and regeneration. *6:e21052*, 1–35.

Fincher, C.T., Wurtzel, O., de Hoog, T., Kravarik, K.M., and Reddien, P.W. (2018). Cell type transcriptome atlas for the planarian Schmidtea mediterranea. Science (80-.). *360*.

Goff, L.A., Trapnell, C., and Kelley, D. (2012). CummeRbund: visualization and exploration of Cufflinks high-throughput sequencing data. R Packag. Version 2.0.

Grohme, M.A., Schloissnig, S., Rozanski, A., Pippel, M., Young, G.R., and Winkler, S. (2018). The genome of Schmidtea mediterranea and the evolution of core cellular mechanisms. Nature *554*, 56–61.

Guo, T., Peters, A.H.F.M., and Newmark, P.A. (2006). A bruno-like Gene Is Required for Stem Cell Maintenance in Planarians. Dev. Cell *11*, 159–169.

Kim, D., Langmead, B., and Salzberg, S.L. (2015). HISAT: A fast spliced aligner with low memory requirements. Nat. Methods *12*, 357–360.

King, R.S., and Newmark, P.A. (2013). In situ hybridization protocol for enhanced detection of gene expression in the planarian Schmidtea mediterranea. BMC Dev. Biol. *13*, 1–16.

Labbé, R.M., Irimia, M., Currie, K.W., Lin, A., Zhu, S.J., Brown, D.D.R., Ross, E.J., Voisin, V., Bader, G.D., Blencowe, B.J., et al. (2012). A Comparative transcriptomic analysis reveals conserved features of stem cell pluripotency in planarians and mammals. Stem Cells *30*, 1734–1745.

Lei, K., McKinney, S.A., Ross, E.J., Lee, H.-C., and Alvarado, A.S. (2019). Cultured pluripotent planarian stem cells retain potency and express proteins from exogenously introduced mRNAs. BioRxiv *573725*.

Mootha, V.K., Lindgren, C.M., Eriksson, K.F., Subramanian, A., Sihag, S., Lehar, J., Puigserver, P., Carlsson, E., Ridderstråle, M., Laurila, E., et al. (2003). PGC-1α-responsive genes involved in oxidative phosphorylation are coordinately downregulated in human diabetes. Nat. Genet. *34*, 267–273.

Plass, M., Solana, J., Alexander Wolf, F., Ayoub, S., Misios, A., Glažar, P., Obermayer, B., Theis, F.J., Kocks, C., and Rajewsky, N. (2018). Cell type atlas and lineage tree of a whole complex animal by single-cell transcriptomics. Science (80-.). *360*.

Subramanian, A., Tamayo, P., Mootha, V.K., Mukherjee, S., Ebert, B.L., Gillette, M.A., Paulovich, A., Pomeroy, S.L., Golub, T.R., Lander, E.S., et al. (2005). Gene set enrichment analysis: A knowledge-based approach for interpreting genome-wide expression profiles. Proc. Natl. Acad. Sci. U. S. A. *102*, 15545–15550.

Trapnell, C., Williams, B.A., Pertea, G., Mortazavi, A., Kwan, G., Van Baren, M.J., Salzberg, S.L., Wold, B.J., and Pachter, L. (2010). Transcript assembly and quantification by RNA-Seq reveals unannotated transcripts and isoform switching during cell differentiation.

Nat. Biotechnol. *28*, 511–515.

Trapnell, C., Hendrickson, D.G., Sauvageau, M., Goff, L., Rinn, J.L., and Pachter, L. (2013). Differential analysis of gene regulation at transcript resolution with RNA-seq. Nat. Biotechnol. *31*, 46–53.

Tu, K.C., Cheng, L.C., Vu, H.T.K., Lange, J.J., McKinney, S.A., Seidel, C.W., and Sánchez Alvarado, A. (2015). Egr-5 is a post-mitotic regulator of planarian epidermal differentiation. Elife e10501.

Wang, I.E., Wagner, D.E., and Reddien, P.W. (2018). Clonal analysis of planarian stem cells by subtotal irradiation and single-cell transplantation. In Planarian Regeneration . Humana Press, New York, NY., pp. 479–495.

Wickham, H. (2009). ggplot2 Elegant Graphics for Data Analysis. Springer.

Van Wolfswinkel, J.C., Wagner, D.E., and Reddien, P.W. (2014). Single-cell analysis reveals functionally distinct classes within the planarian stem cell compartment. Cell Stem Cell *15*, 326–339.

Zeng, A., Li, H., Guo, L., Gao, X., McKinney, S., Wang, Y., Yu, Z., Park, J., Semerad, C., Ross, E., et al. (2018). Prospectively Isolated Tetraspanin+Neoblasts Are Adult Pluripotent Stem Cells Underlying Planaria Regeneration. Cell 1593–1608.
