# Supplementary material for: Mitochondrial state determines functionally divergent stem cell population in planaria
Source: Stem Cell Reports. 2021 Apr 15;16(5):1302–16. doi: 10.1016/j.stemcr.2021.03.022 (PMC8185449; doi:10.1016/j.stemcr.2021.03.022)
Supplement: Document S2. Article plus Supplemental information [file mmc5.pdf]

Stem Cell Reports
Article
OPEN ACCESS

Mitochondrial state determines functionally divergent stem cell population in planaria

Mohamed Mohamed Haroon,[1,2] Vairavan Lakshmanan,[1,2] Souradeep R. Sarkar,[3] Kai Lei,[4,5] Praveen Kumar Vemula,[1,*] and Dasaradhi Palakodeti[1,*]
[1]Integrative Chemical Biology, Institute for Stem Cell Science and Regenerative Medicine, Bengaluru, India
[2]SASTRA University, Thirumalaisamudram, Thanjavur, India
[3]National Centre for Biological Sciences, Bengaluru, India
[4]Zhejiang Provincial Laboratory of Life Sciences and Biomedicine, Key Laboratory of Growth Regulation and Translational Research of Zhejiang Province, School of Life Sciences, Westlake University, Hangzhou, Zhejiang Province, China
[5]Institute of Biology, Westlake Institute for Advanced Study, Hangzhou, Zhejiang Province, China
*Correspondence: praveenv@instem.res.in (P.K.V.), dasaradhip@instem.res.in (D.P.)
https://doi.org/10.1016/j.stemcr.2021.03.022

SUMMARY

Mitochondrial state changes were shown to be critical for stem cell function. However, variation in the mitochondrial content in stem cells and the implication, if any, on differentiation is poorly understood. Here, using cellular and molecular studies, we show that the planarian pluripotent stem cells (PSCs) have low mitochondrial mass compared with their progenitors. Transplantation experiments provided functional validation that neoblasts with low mitochondrial mass are the true PSCs. Further, the mitochondrial mass correlated with OxPhos and inhibiting the transition to OxPhos dependent metabolism in cultured cells resulted in higher PSCs. In summary, we show that low mitochondrial mass is a hallmark of PSCs in planaria and provide a mechanism to isolate live, functionally active, PSCs from different cell cycle stages (G0/G1 and S, G2/M). Our study demonstrates that the change in mitochondrial metabolism, a feature of PSCs is conserved in planaria and highlights its role in organismal regeneration.

INTRODUCTION

Neoblasts are adult pluripotent stem cells (PSCs) that play a central role in planarian regeneration. They are capable of differentiating into all other cell types, thereby facilitating the regeneration of lost tissues. On injury, planarians induce wound healing response leading to stem cell proliferation and differentiation, followed by remodeling of old and new tissue to form a completely regenerated animal (Reddien, 2018). In planarians, neoblasts are the sole proliferating cells and eliminating them by irradiation results in lethality (Newmark and Sánchez Alvarado, 2000; Reddien and Alvarado, 2004). Our understanding of the mechanisms that regulate pluripotency is limited by the capacity to isolate the PSCs from planaria.

The current technique to isolate neoblasts involves fluorescence-activated cell sorting (FACS) based on nuclear density by staining the cells with Hoechst (Hayashi et al., 2006). Hoechst staining identified three major populations: X1, X2, and Xins. X1 cells are the proliferating neoblasts with >2N nuclear content (S and G2/M phases of the cell cycle) and expression of stem cell markers such as *piwi-1, vasa-1, bruli* (Reddien et al., 2005; Wagner et al., 2012). X2 includes post-mitotic progenitor cells as well as stem cells in the G0/G1 phase of the cell cycle (Eisenhoffer et al., 2008; Molinaro and Pearson, 2016; Van Wolfswinkel et al., 2014). Both X1, X2 are eliminated on X-ray irradiation, whereas the Xins population is insensitive to irradiation and constitutes terminally differentiated cells. Since the neoblasts are the only dividing cells in planaria, the X1 population has been extensively used for functional characterization of the neoblast. So far, our understanding of the stem cells in the X2 gate was limited, as it has not been possible to isolate stem cells from this gate. Therefore, the current knowledge about the neoblasts was obtained by studying the X1 population (>2N cells).

Detailed molecular analysis showed that the X1 population consists of pluripotent (clonogenic neoblasts) and primed stem cells (specialized neoblasts) (Fincher et al., 2018; Plass et al., 2018; Van Wolfswinkel et al., 2014; Wagner et al., 2011). Clonogenic neoblasts were shown to rescue planarians upon lethal irradiation. Recently, it was shown that a subset of neoblasts, which express tetraspanin-1 (TSPAN-1) are pluripotent in nature. Single-cell transplantation of TSPAN-1+ cells in lethally irradiated planarians rescued the host with greater efficiency validating its pluripotency (Zeng et al., 2018). The specialized neoblasts are defined by the expression of transcripts pertaining to specific lineages in addition to pan neoblast markers such as *piwi-1*. However, a lack of strong evidence to show that all the PSCs are TSPAN + suggests that there could be a possibility of a subset of PSCs that lack the expression of TSPAN.

Mitochondria, a hub for cellular energetics, have been shown to be critical for stem cell maintenance and differentiation (Folmes et al., 2012; Xu et al., 2013). Evidence shows that mitochondria in PSCs exist in a discontinuous fission state with very low oxidative phosphorylation (OxPhos).

This is an open access article under the CC BY-NC-ND license (http://creativecommons.org/licenses/by-nc-nd/4.0/).

Several studies have used differences in mitochondrial activity as a means to demarcate stem cells from their respective progenitors. For example, hematopoietic stem cells (HSCs) with low MitoTrackerTM Green (MTG) staining exhibited enhanced stemness and showed greater reconstitution potential compared with their MTG High counterpart (Romero-Moya et al., 2013). Similarly, tetramethylrhodamine methyl ester (TMRM) a mitochondrial membrane potential sensing dye was used to separate minimally differentiated stem cell-memory T cells from highly differentiated effector memory T cells (Sukumar et al., 2016).

This study aimed to understand the differences in mitochondrial content between the planarian cell populations, its functional consequence on stemness, and employ this as a means to distinguish stem cell states. Based on the mitochondrial dye MTG, we were able to further distinguish cells within the X1 and the X2 cell populations as X1-MTG^{High}, X1-MTG^{Low}, X2-MTG^{High}, and X2-MTG^{Low} cells. Transcriptome analysis and molecular characterization of these cells revealed that the X1 cells with low mitochondrial mass were a more homogeneous pool of PSCs compared with cells with higher mitochondrial mass. The X1-MTG^{High} cells expressed genes essential for lineage commitment exhibiting the signatures of committed neoblasts. A similar analysis of X2 revealed that the cells with low mitochondrial mass were either stem cells in the G0/G1 phase or early progenitors, whereas the cells with high mitochondrial mass were late progenitors. Further, to validate the pluripotency, cells isolated from the asexual strain (donor) were transplanted into lethally irradiated sexual strain (host). Our results indicate that the X1 and a subset of X2 cells with low MTG were able to rescue and thus transform the sexual planaria into asexual planaria at higher efficiencies compared with the MTG^{High} cells. Our study also revealed that the mitochondrial activity correlates with mass and inhibiting the mitochondrial activity by treating the neoblast with FCCP *in vitro* resulted in increased stemness as validated by the transplantation experiments. Together, our results show that the PSCs in the planarians can be categorized by low mitochondrial mass compared with their immediate progenitors. Further, the changes in the mitochondrial mass between pluripotent neoblast and the committed neoblast could be used to isolate PSCs from planarians for functional characterization.

RESULTS

Mitochondrial staining reveals a distinct mitochondrial mass in planarian X1, X2, Xins cells

Single-cell transcriptome studies in planarians revealed neoblasts as a heterogeneous pool of clonogenic and specialized stem cells. Here, we investigated how the mitochondrial mass changes between different planarian cell populations. This was tested by staining the neoblast population with MTG, a fluorescent dye that is used as a measure for the mitochondrial mass (Doherty and Perl, 2017; Romero-Moya et al., 2013). The cell suspension of planaria was stained with Hoechst 33,342 (nuclear dye) and MTG. The cells stained with Hoechst showed three distinct populations, X1, X2, and Xins, based on their nuclear content (Figure 1A). X1 cells, which are the proliferating stem cells, have a lower MTG signal than the Xins population (differentiated cells) (Figures 1B and 1C). The X2 population, majorly neoblast progenitor, had a broad distribution of the MTG signal (Figures 1B and 1C). MTG fluorescence in most X1 cells was more perinuclear and exhibited polarized distribution in the cytoplasm (Figures 1D and S1). Conversely, MTG fluorescence in Xins cells, in general, was evenly distributed in the cytoplasm (Figures 1D and S1). To test whether the low MTG intensity in X1 and X2 cells was a consequence of dye efflux, the cells were treated with Verapamil, an efflux pump inhibitor, and stained with MTG. There was no significant increase in the median fluorescence intensity in the X1 population on treatment with Verapamil (Figure 1E), suggesting that the lower MTG fluorescence in X1 cells is a true indicator of decreased mitochondrial mass. Together, these results suggest that the difference in the mitochondrial mass between X1 and Xins cells could be an indicator of the stem versus differentiated state.

It has been shown that mammalian embryonic stem cells show distinct mitochondrial morphology and their activity compared with their progenitors (Xu et al., 2013). However, the change in the mitochondrial content and its regulation of pluripotency was poorly understood. We examined whether the mitochondrial mass could distinguish the clonogenic and specialized neoblast pools. To this end, molecular and functional studies were performed in X1 cells with high and low mitochondrial mass as measured by MTG.

Low MTG enriches for PIWI-1^{High} cells within the X1 population

In addition to the change in the mitochondrial mass between X1 and Xins, we also identified cells with high and low MTG signals within the X1 population (Figure 2A). Here, we wanted to investigate the pluripotent state of the MTG^{Low} and MTG^{High} cells in the X1 population. *Piwi-1* is a nuage-related gene that has been extensively used to mark stem cells in planaria. To examine the status of *piwi-1* in MTG^{Low} and MTG^{High} cells, we first generated and characterized an antibody to PIWI-1 (Guo et al., 2006). Previous studies have shown that clonogenic neoblast and a subset of the neoblast population have high levels of PIWI-1 protein

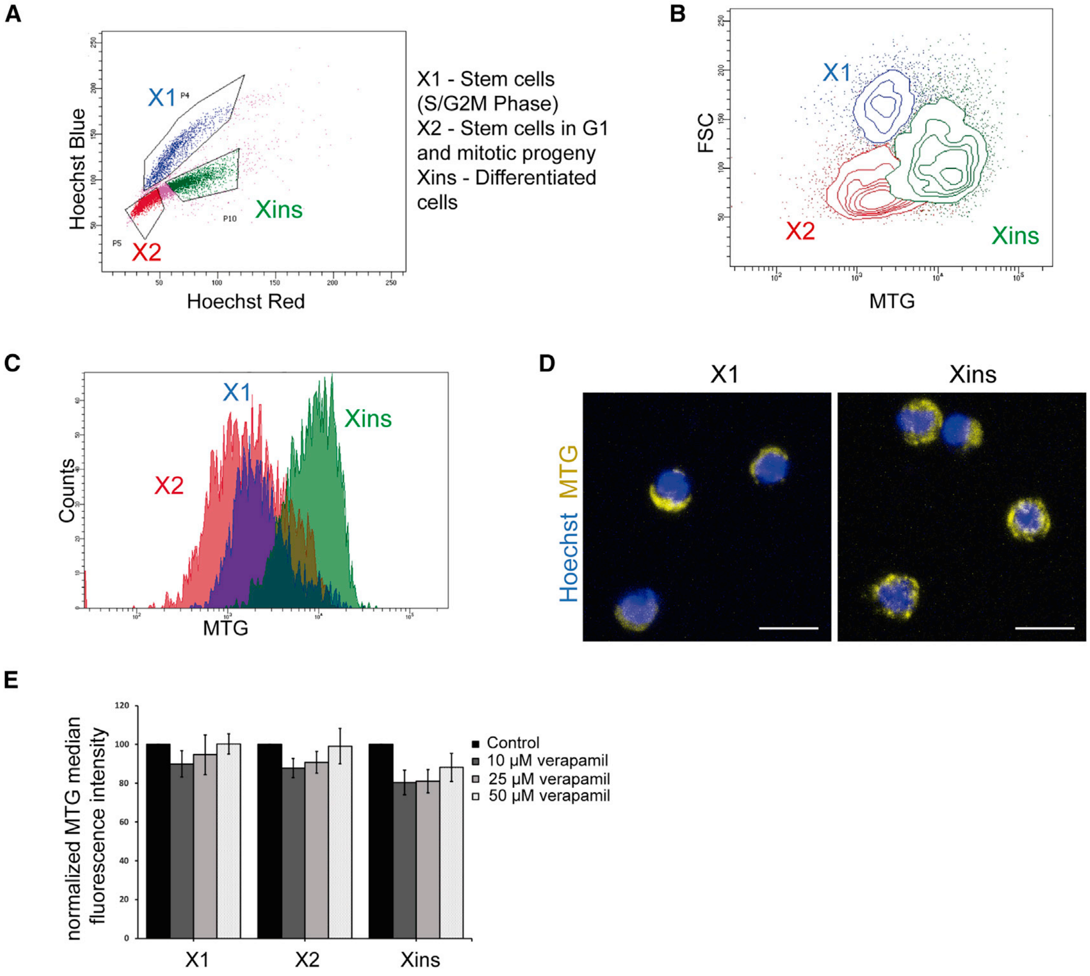

Figure 1. MitoTracker Green FM staining in planarian cells

(A) Flow cytometry analysis of planarian cell suspension stained with Hoechst 33,342 revealing X1, X2, and Xins population.

(B and C) Representative contour plot (B) and histogram (C) showing MTG intensity of X1, X2, and Xins cells.

(D) Representative confocal images showing MTG staining in sorted, live X1, and Xins cells. Scale bar, 10 μm.

(E) Flow cytometry analysis of MTG median fluorescent intensity of X1, X2, and Xins cells in the presence of indicated amounts of Verapamil. Mean ± SEM, over three independent replicates.

and its corresponding RNA, whereas the progenitors have low levels of PIWI-1 protein (Zeng et al., 2018). The immuno-staining with PIWI-1 antibody in planarian cells identified High, Low, and negative PIWI-1 cells (Figures S2A and S2B). Further, we have also shown that PIWI-1^{High} cells are the proliferating cells, which are in the S, G2/M phase, whereas most of the PIWI-1$^{Low/Negative}$ cells are in the G1 phase as was reported in earlier studies (Zeng et al., 2018) (Figure S2C). This was also validated by immunostaining of X1, X2, and Xins cells for PIWI-1 protein, where X1 cells showed high PIWI-1 compared with X2 and Xins (Figures S2D and S2E). Together these results show that the PIWI-1 antibody recognizes the neoblast population.

Next, we examined the expression of PIWI-1 in MTG^{High} and MTG^{Low} cells within the X1 population (Figure 2A). We observed that X1-MTG^{Low} cells had a higher representation of PIWI-1^{High} cells compared with X1-MTG^{High} cells (Figures 2B and 2C). Conversely, cells from X1-MTG^{High} cells were enriched in PIWI-1$^{Low/Negative}$ cells. We speculate that enrichment of PIWI-1$^{Low/Negative}$ cells in X1-MTG^{High} could be the committed NB clusters and the X1-MTG^{Low} cells could either be the noncommitted neoblast or the clonogenic population.

Transcriptome sequencing of high and low MTG reveal neoblast heterogeneity within X1 cells

In order to understand the functional states of High and Low MTG cells, RNA sequencing (RNA-seq) was performed on X1-MTG^{Low} and X1-MTG^{High} populations (Figure 2D). Transcriptome sequencing followed by Cuffdiff (Trapnell et al.,

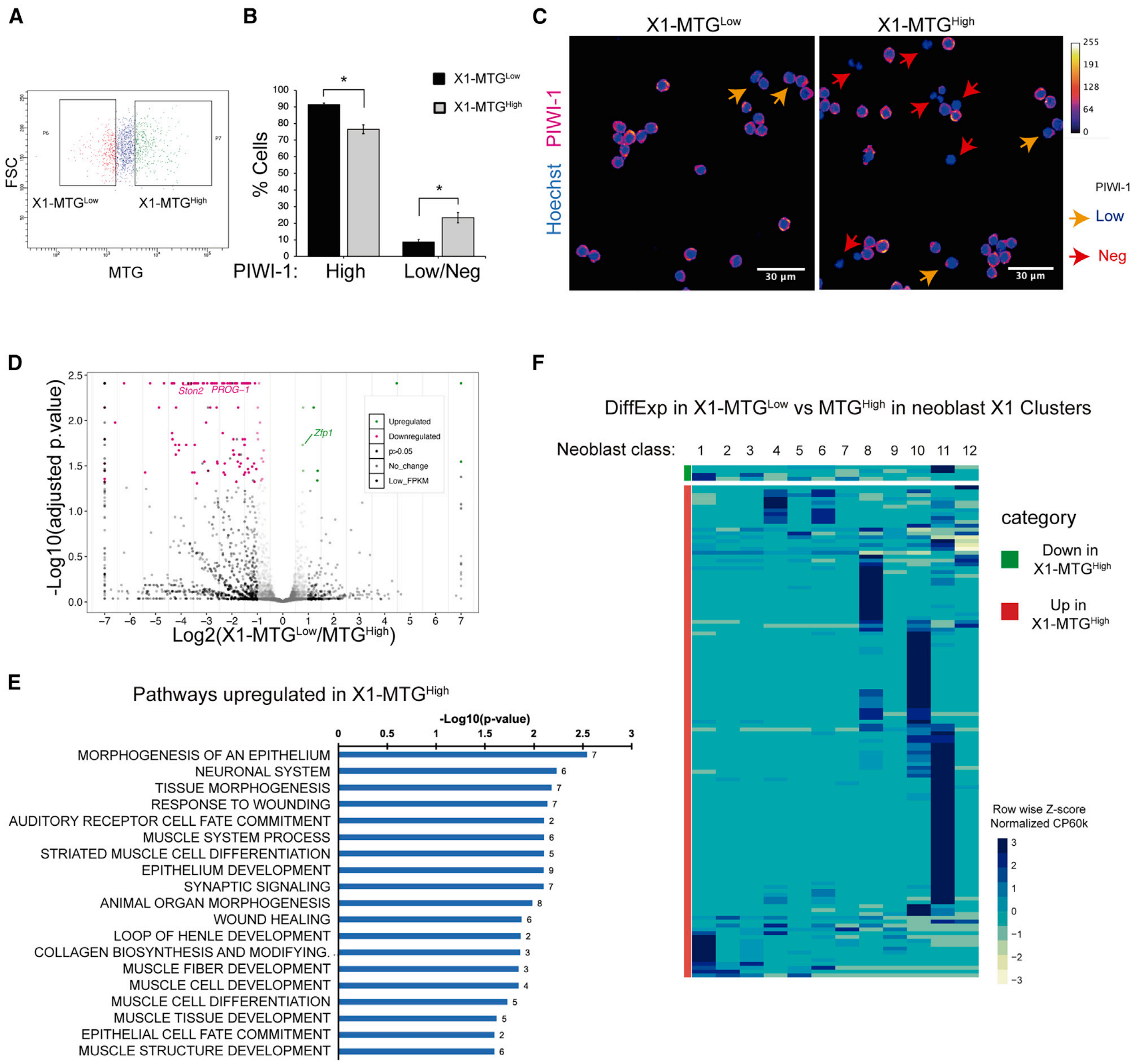

Figure 2. MTG staining reveals heterogeneity in X1 cells

(A) Dot plot showing the FACS gating for X1-MTG^{Low} and MTG^{High} cells.

(B) PIWI-1 immunostaining in FACS sorted X1-MTG^{Low} and MTG^{High} cells quantified for PIWI-1^{High} through flow cytometry. Mean ± SEM, over three independent replicates. *Represents $p < 0.05$. See also Figure S1.

(C) Representative confocal images of PIWI-1 staining in indicated sorted population. PIWI-1 staining is shown using Fire LUT (ImageJ). Arrowheads highlight PIWI-1 low (yellow) and negative (red) cells. Scale bar, 30 μm.

(D) Volcano plot displaying differential gene expression between X1-MTG^{Low} and MTG^{High} cells.

(E) Bar graph representing selected GO terms that are downregulated in X1-MTG^{Low} cells compared with MTG^{High}. Refer to Table S1 for the full list of GO terms.

(F) Heatmap indicating the expression of the differentially expressed genes in X1-MTG^{Low} vs MTG^{High} cells in the published single-cell database (Zeng et al., Cell 2018).

2013) analysis for differential expression identified 172 differentially regulated genes from X1-MTG^{Low} and MTG^{High} cells (Figure 2D). Gene ontology (GO) analysis of upregulated ($p < 0.05$) genes (163) in X1-MTG^{High} cells showed that most of these genes are essential for differentiation (muscle, epithelium, neural system), wound response, and tissue

morphogenesis (Figure 2E and Table S1). For instance, our analysis identified two key markers of epidermal and neural progenitors (*prog-1* and *ston-2)* upregulated by >2-fold ($p < 0.05$) in X1-MTG^{High} cells. Next, the differentially expressed genes were compared with the published single-cell transcriptome data from X1 (Zeng et al., 2018). Most of the genes enriched in X1-MTG^{High} have high expression in NB classes other than NB2, which is the clonogenic neoblast population, suggesting that the X1-MTG^{High} cells are mostly committed neoblasts (Figure 2F). Interestingly, we did not observe any genes significantly upregulated in the X1-MTG^{Low} population that belong to the NB2 class (clonogenic neoblast) such as *tspan-1*, *tgs-1,* and *pks-1*. Together, these results indicate that within X1, the MTG^{High} cells represent the committed neoblast and the MTG^{Low} cells could potentially contain pluripotent neoblast cells.

Encouraged by these results, we then explored the cell states of the X2 population based on MTG signal. Cells with low and high MTG signals within the X2 population were subjected to PIWI-1 immunostaining and transcriptome analysis.

Low and high MTG X2 cells show heterogeneous pool of PIWI-1$^+$ and PIWI-1$^-$ populations, respectively

Previous studies showed that the X2 population consists of neoblasts and their progenitors (Eisenhoffer et al., 2008; Van Wolfswinkel et al., 2014). Our results based on MTG staining showed that the X2 population can be broadly categorized as MTG^{Low} and MTG^{High} cells. To investigate the stem state of the MTG^{Low} and MTG^{High} cells in the X2 population, we stained them with PIWI-1 antibody, which marks the neoblast populations. The X2-MTG^{Low} cells have a mixed population of PIWI-1^{High} and $^{-Low}$. Flow cytometry analysis based on the size showed PIWI-1^{High} cells are large in size compared with the PIWI-1 low cells (Figure S2A) (Zeng et al., 2018). Hence, to separate PIWI-1^{High} cells in the G1 phase, we further sorted the X2-MTG^{Low} and X2-MTG^{High} cells based on their size (FSC), which resulted in four populations (X2-MTG^{Low} High and Low FSC cells; X2-MTG^{High} High and Low FSC cells) (Figure 3A). The antibody staining indicated that within the X2-MTG^{Low}, ~35% of HFSC (High FSC) cells showed higher expression of PIWI-1, while LFSC (Low FSC) cells had lower PIWI-1 expression (~85% cells) (Figures 3B and 3C). Regardless of the size, the majority of the X2-MTG^{High} cells either showed low or negative expression for PIWI-1 (Figures 3B and 3C). Together, these data suggest that X2-MTG^{Low}-(HFSC) cells, which were PIWI-1^{High}, are likely to be neoblast population in the G1 phase. Whereas the X2-MTG^{Low}-(LFSC) cells, which are PIWI-1^{Low} could be the early progenitors. Conversely, X2-MTG^{High} (High and Low FSC) cells that are PIWI-1 negative could potentially be late progenitors.

Transcriptome sequencing shows enrichment of neoblast and their early progenitors in the X2-MTG^{Low} population

To gain further insights into the nature of the cells in the MTG^{Low} and MTG^{High} populations of X2, we performed RNA-seq on the four populations. RNA-seq analysis of X2-MTG^{Low} and MTG^{High} cells (High and Low FSC combined) revealed that the MTG^{Low} population is enriched with transcripts of stemness, nuage-related neoblast markers (*smedwi-1, smedwi-3, bruli*), cell cycle genes (*rrm2, mcm*), and early markers for differentiation compared with MTG^{High} (Figures 4A, 4B, and S3A and Table S2). Further, we also compared the RNA profile of the MTG subpopulations to the total X1, X2, and Xins populations isolated by conventional methods. Based on principal component analysis, correlation plot, and linkage mapping (Figures 4C, D, and S3B), the transcript signatures of X2-MTG^{Low} showed a strong correlation (R^2 = 0.8991) with the X1 population compared with X2-MTG^{High}, which was closer to Xins. Further, we also observed that most of the transcripts related to early epidermal progenitors (*prog-1* and *agat-1*) were enriched in X2-MTG^{Low}, while the transcripts of late epidermal progenitors (*zpuf-6*) and the terminally differentiated epidermal cells were enriched in X2-MTG^{High} (Figures S3C). Moreover, pseudotime expression analysis using the available planarian single-cell RNA-seq database showed that the expression of transcripts enriched in X2-MTG^{Low} peaked in the neoblast class and decreased as the cells differentiated. In contrast, transcripts enriched in the X2-MTG^{High} showed negligible expression in neoblast populations and increased as the cells differentiate (Figures S4A–S4C). These analyses clearly show that the cells in X2-MTG^{Low} are either the neoblast cells in the G1 phase or their immediate/early progenitors. Conversely, the cells in X2-MTG^{High} were mostly in the late phase of differentiation. Thus, our results suggest that the changes in mitochondrial content reflect the cell transition states within X2.

Next, we analyzed the transcriptome data from both High and Low FSC events from X2 MTG^{Low}. GO analysis showed that the differentially expressed genes that are upregulated ($p < 0.05$) in X2-MTG^{Low}-(HFSC) cells are involved in cell cycle and DNA synthesis indicating these might be the stem cells in G0/G1 phase poised to potentially enter the cell cycle (Figure 4E). On the contrary, GO analysis for the transcripts enriched in X2-MTG^{Low}-(LFSC) cells encode proteins that were involved in differentiation suggesting that these cells are the immediate/early progenitors (Figure 4F and Table S3). To rule out the possibility of S-phase cells contaminating the X2-MTG^{Low}-(HFSC) gate, cell cycle analysis was performed in sorted cells. The results clearly indicate that both LFSC and HFSC cells from the X2-MTG^{Low} gate are in G0/G1 state

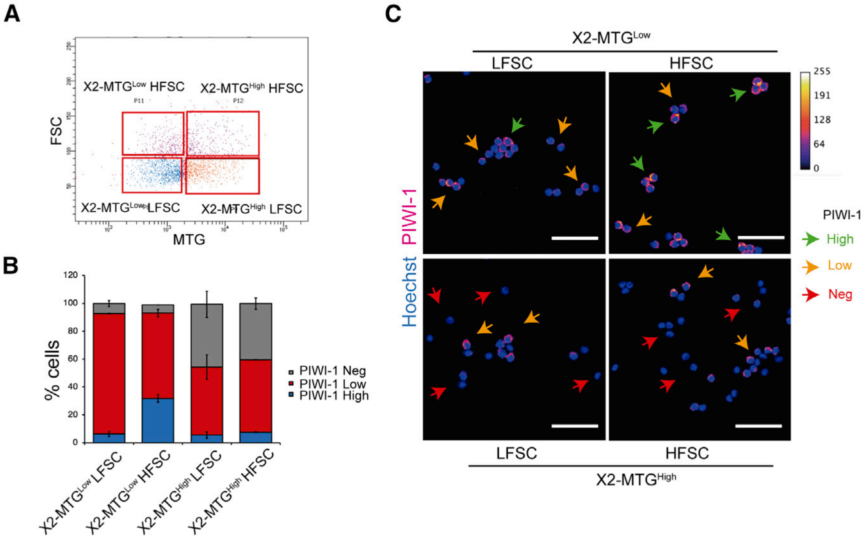

Figure 3. PIWI-1 immunostaining in X2-MTG subpopulations

(A) Dot plot representing the gating strategy for various MTG populations. HFSC - High Forward Scatter, LFSC – Low Forward Scatter.

(B) Quantitative flow cytometry analysis of PIWI-1 staining in indicated FACS sorted X2 MTG population. Mean ± SEM, over three independent replicates.

(C) Representative confocal images of PIWI-1 staining in indicated X2 MTG populations. PIWI-1 staining is represented in Fire LUT (ImageJ) and the pixel value and the corresponding color code are given in the graph (top right). Arrowheads indicate PIWI-1 high (green), low (yellow), and negative (red) cells. Scale bar, 30 μm.

See also Figure S1.

and in contrast, the X1-MTG^{Low} cells are in S and G2 phase (Figure S5A). Further, the principal component analysis and correlation plot revealed that the transcripts from both the High and Low FSC cells of X2-MTG^{Low} show high correlation with the X1 population (R^2 = 0.9 HFSC cells and R^2 = 0.89 LFSC cells) suggesting that the X2-MTG^{Low}-(HFSC) gate contains cells that are most likely the neoblast population in G1 phase (Figures S5B and S5C). Together, these results show that MTG along with Hoechst will be a good marker to identify and isolate neoblast cells in the G0/G1 phase.

Pluripotency is associated with stem cells having low mitochondrial mass

The PIWI-1 expression and the transcriptome analysis revealed that the X1 and X2-MTG^{Low}-(HFSC) cells could potentially be clonogenic neoblasts compared with their MTG^{High} counterparts. This was validated by a transplantation experiment that involved the injection of the MTG populations into lethally irradiated animals. The population of cells that rescue the irradiated animals would be considered PSCs (Lei et al., 2019; Wagner et al., 2011). Recent studies have shown that injection of neoblasts from asexual into irradiated sexual planaria transformed sexual strain to asexuals (Lei et al., 2019; Wagner et al., 2011). For our transplantation experiments, we used SiR-DNA (Cytoskeleton inc.), a nuclear dye, recently used as an alternative to isolating 4N cells from planarians instead of Hoechst dye, which was shown to be toxic (Lei et al., 2019; Wagner et al., 2011; Wang et al., 2018b). First, the X1(FS) and X2(FS) gates were set using the Hoechst dye to isolate the 4N cells and 2N cells from asexual planarians. The same gating parameters were used to separate the SiR-DNA 4N cells and SiR-DNA 2N cells to reduce the cross-contamination between X1, X2, and Xins cells. The SiR-DNA cells were stained with MTG to demarcate the MTG^{Low} and MTG^{High} cells within the X1 and X2 population. To understand the PSC activity of the MTG populations, we performed *piwi-1* colony expansion assay and animal survival studies wherein the six different populations of the sorted cells from the asexual strain were transplanted into an irradiated sexual strain. Animals that were not transplanted were used as the negative control. The animals after 8 days posttransplantation (dpt) were fixed and assayed for the presence of *piwi-1*$^+$ colonies (Figure 5A). We found that 4N-MTG^{Low} cells (X1-MTG^{Low} equivalent) had the maximum number of *piwi-1*$^+$ colonies compared with the 4N-MTG^{High} (Figure 5B). Similarly, among the 2N cells (X2 equivalent), only the 2N-MTG^{Low} (HFSC) cells showed a greater number of *piwi-1*$^+$ colony expansion (Figure 5B). None of the other three populations (X2-MTG^{Low}-LFSC, X2-MTG^{High} High, and low FSC cells) showed any *piwi-1*$^+$ colonies. We also performed survival studies in the transplanted and nontransplanted animals post lethal dose of irradiation. Typically, the nontransplanted sexual animals, post-irradiation, show head regression, ventral curling, and eventually lysed after 30 dpt. However, the sexual animals rescued with asexual neoblast eventually show fission, which is characteristic of asexual animals (Figures 5C and 5D). This result indicates that the transplanted cells have divided and replaced all the cells in the sexual (Lei et al., 2019; Wagner et al., 2011). Within the 4N cells (X1 equivalent), the MTG^{Low} population showed twice the rescue efficiency (63.33%) compared with 4N-MTG^{High} (30%) (Figures 5E and 5F). These results conclusively show that the neoblasts with low MTG are more pluripotent than the

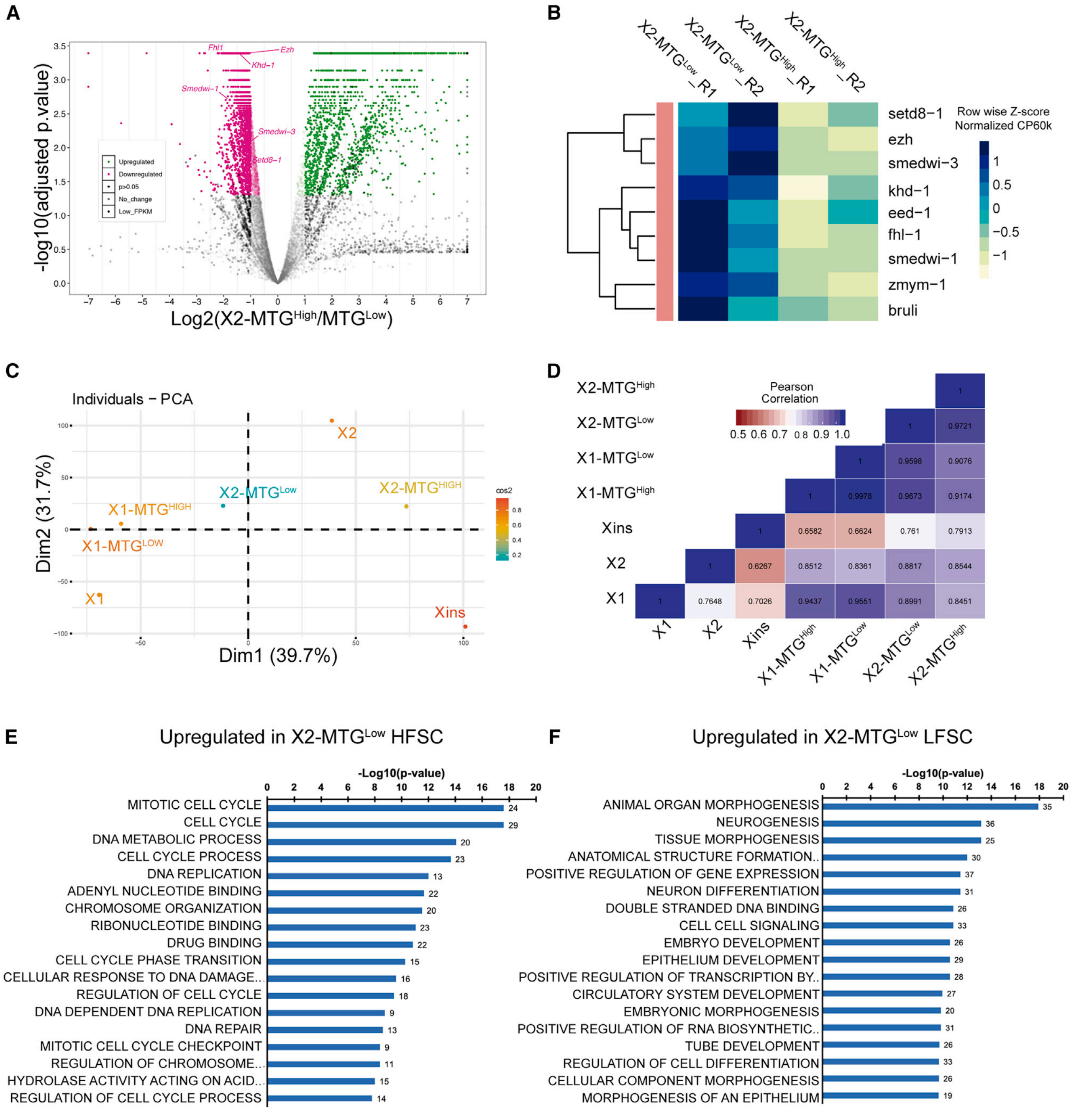

Figure 4. Transcriptome analysis of X2-MTG^{Low} and MTG^{High} cells

(A) Volcano plot showing the differentially expressed transcripts. Major stem cell genes >2-fold upregulated in MTG Low are marked in the plot.

(B) Heatmap showing expression of key stem cell-related genes in X2-MTG^{Low} and MTG^{High}. R1 and R2 represent biological replicates.

(C) Principal component analysis (PCA) showing indicated MTG population with respect to X1, X2, and Xins

(D) Pearson's correlation heatmap of the X2 MTG population compared with X1, X2, and Xins. See also Figures S2 and S3 and Table S2.

(E) GO terms of statistically significant, >2-fold upregulated transcripts from X2-MTG^{Low}-HFSC cells compared with X2-MTG^{Low}-LFSC cells.

(F) GO terms of upregulated transcripts from the X2-MTG^{Low}-LFSC cells compared with corresponding HFSC cells. See also Table S3.

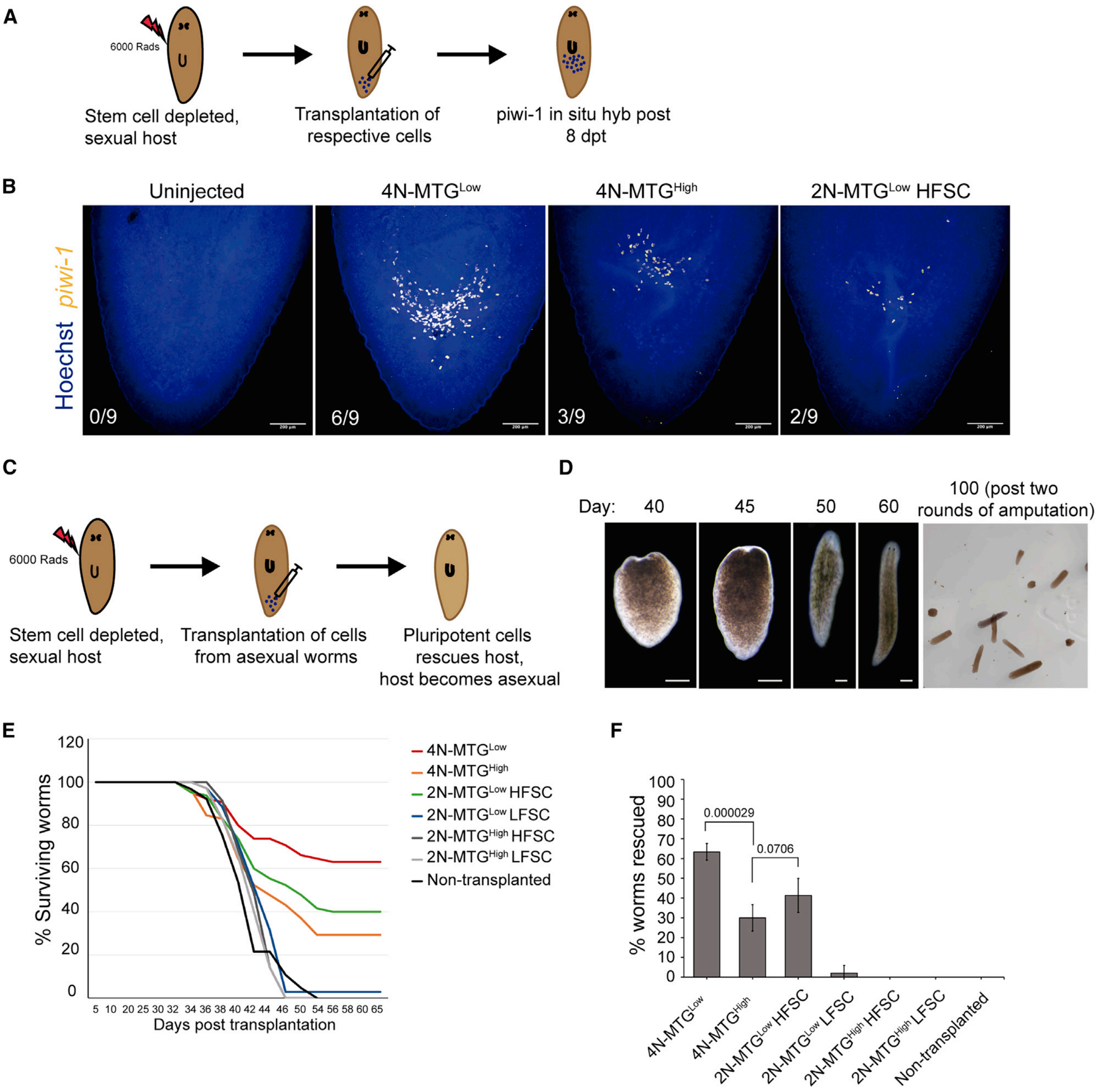

Figure 5. Transplantation experiment to validate the pluripotency of MTG populations

(A) Schematic of colony expansion assay performed.

(B) Representative *piwi-1* staining in lethally irradiated sexual worms 8 dpt of ~1000 cells from asexual, SiR-DNA stained MTG populations (see methods). Numbers indicate worms showing *piwi-1* colonies. Scale bar, 200 μm. n = 9 worms.

(C) Schematic showing the long-term survival and transformation of sexuals to asexuals by clonogenic neoblast transplantation.

(D) Representative images of worms surviving after receiving clonogenic neoblasts. Initially, the worms show head regression, and approximately 40 to 45 dpt, worms start to develop blastema and completely regenerate the head. After two rounds of amputation, the worms started propagating via fission. Scale bar, 500 μm.

(E) Plots showing the percentage of surviving worms transplanted with ~1500 cells of the respective MTG population. n = 65 worms for 4N-MTG^{Low} and $^{-High}$, 2N-MTG^{Low}(HFSC), uninjected control. And n = 35 for the rest of the population performed in five independent replicates.

(legend continued on next page)

neoblasts with high MTG. Interestingly, the 2N-MTG^{Low}-(HFSC) cells, showed 41.33% rescue efficiency compared with 2N-MTG^{Low} (LFSC) cells (2%, 1 of 35 animals were rescued). As expected, both the 2N-MTG^{High} (small and large cells) failed to rescue the animals (Figures 5E and 5F). The new gating strategy based on mitochondrial content described in this study experimentally validates the presence of clonogenic neoblasts within an X2 equivalent gate. Moreover, despite the presence of only ~35% PIWI-1^{High} cells, the X2-MTG^{Low}-(HFSC) cells exhibited higher rescue efficiency compared with X1 MTG^{High} cells indicating a greater clonogenic potential (Figure S6). Together, our transplantation experiments show that the decreased mitochondrial mass is a hallmark of PSCs in planarians. In contrast, the increased mitochondrial mass is an indicator of stem cell differentiation.

Increased mitochondrial potential is essential for neoblast differentiation

To understand the mitochondrial activity of MTG^{Low} and $^{-High}$ cells, planarian cells were simultaneously stained with MTG and MitoTracker Orange (MTO), a dye that indicates mitochondrial membrane potential (Figure S7A). It was observed that MTG^{Low} cells were also low in MTO and conversely MTG^{High} cells had high MTO intensity (Figures 6A and A′). A similar trend was also observed upon normalization of the MTO intensity to the cells treated with FCCP, an uncoupler of oxidative phosphorylation (Figures 6A and A′). This clearly indicates that the mitochondrial content correlates with mitochondrial activity. Next, we investigated the implication of changes in mitochondrial activity on stem cell differentiation. We treated the regenerating worms with FCCP. We chose low doses of FCCP (30 nM) to treat the regenerating animals, as the concentrations above 50 nM were toxic to the animals and lyse after 12 to 16 h of treatment. The worms treated with FCCP showed a 24 to 48-h delay in regeneration as revealed by the appearance of the eyespots (Figure 6B). RNA FISH and qPCR analysis for *piwi-1* showed no significant change in transcript levels between control and FCCP-treated worms (Figures 6C, 6D, and 6E). This indicates that the delay in regeneration could be attributed to FCCP hampering the differentiation process rather the affecting the viability of the stem cells. However, it is difficult to make definitive conclusions because the low doses of FCCP might be suboptimal and the reduction of mitochondrial activity *in vivo* is global and not restricted to stem cells. To specifically perturb the mitochondrial activity in stem cells, we cultured the neoblasts and treated them with FCCP (Figure 6F). Toward this, we used SiR-DNA (Cytoskeleton inc.) and sorted 4N cells which were also MTG^{Low} (X1-MTG^{Low} equivalent) and cultured as described by (Lei et al., 2019). The current cell culture system for neoblast is not completely optimized and it is observed that with time the cells differentiate and beyond 48 h lose their clonogenic potential (Lei et al., 2019). We observed that post 48 h of culture, there was a higher number of PIWI-1^{High} cells in FCCP-treated cells compared with the DMSO control (Figure 6G). Such an increase in PIWI-1^{High} cells was not a consequence of accelerated cell death as the viability of both High and Low MTG populations does not change upon treatment with 200 nM FCCP *in vitro* (Figures S7B and S7C). Transplantation assays were performed to functionally validate the stemness of the FCCP-treated neoblast. The results indicate that cells treated with 200 nM FCCP showed higher colony expansion than the control cells (Figures 6H and 6I). We then performed the rescue experiments, which revealed that 200 nM FCCP-treated cells showed ~16.67% rescue efficiency (Figure 6J), while the DMSO control cells failed to rescue any animals. Together, these results show that the cells that were treated with FCCP *in vitro* resisted differentiation, and were maintained in the pluripotent state compared with the untreated control cells. Collectively, these results demonstrate that the differences in the mitochondrial content in the stem versus differentiated populations correlate with the mitochondrial activity and also suggest that an increased mitochondrial activity could be a requisite for neoblast differentiation.

DISCUSSION

Planarians are highly regenerative animals that harbor PSCs, which differentiate to lineage primed progenitors thereby controlling the extent of regeneration and tissue turnover during homeostasis (Reddien, 2018). The presence of PSCs in the adult animal makes planaria an excellent model system to study stem cell dynamics. Earlier studies using electron microscopy had shown irregular mitochondrial morphology in the neoblast and most of the mitochondria were located toward the periphery of the nuclear membrane (Hay and Coward, 1975; Morita et al., 1969; Pedersen, 1959). Also, previous studies have shown an increase in the ROS (reactive oxygen species)

(F) Bar graph representing the average percentage of worms rescued. Error bar represents standard deviation, over five independent experiments. n = 65 worms for 4N-MTG^{Low} and $^{-High}$, 2N-MTG^{Low} (HFSC), and uninjected control. n = 35 for the rest of the population. Mean ± SEM, numbers on the graph represent the respective p value.
See also Figure S6.

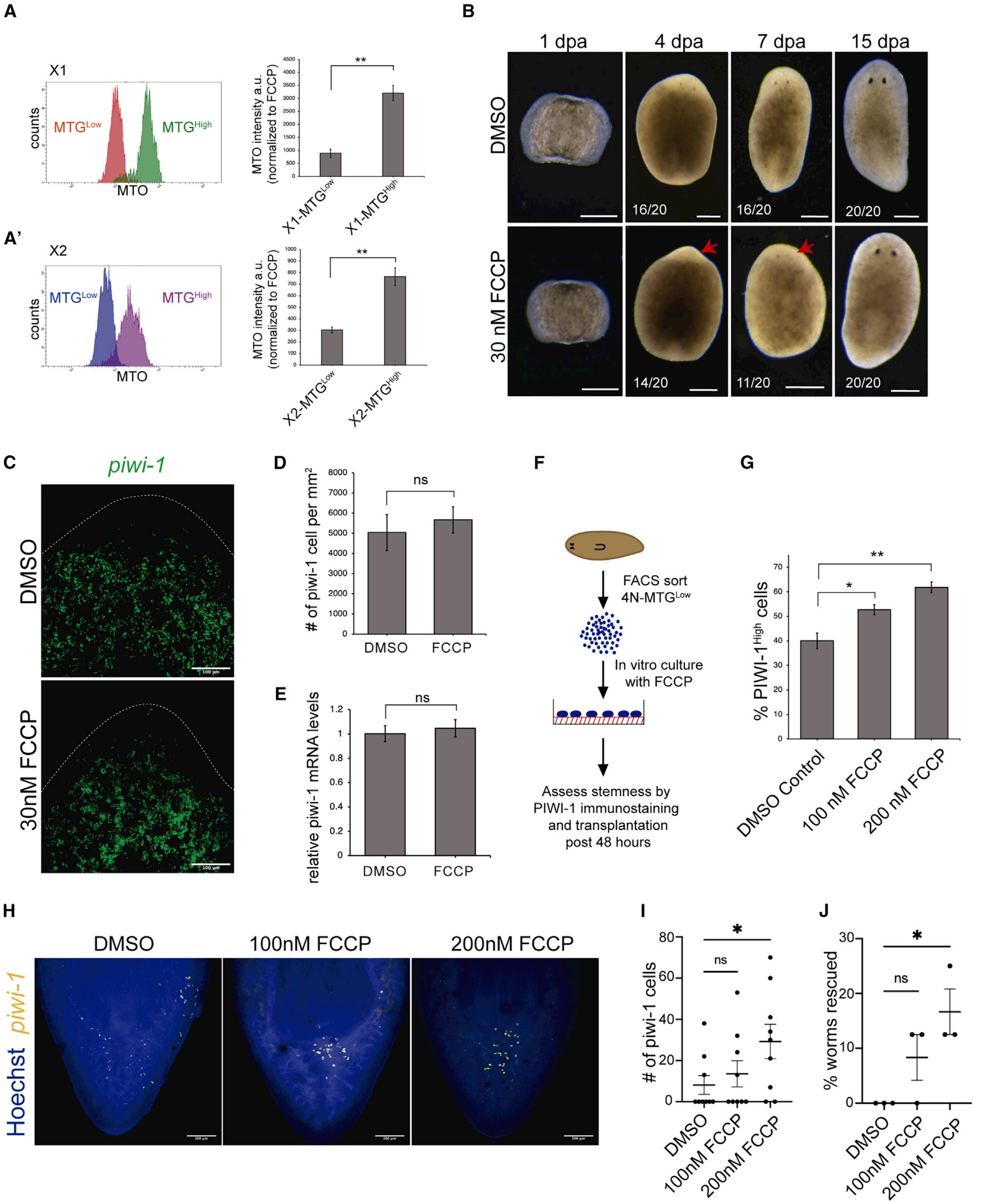

(legend on next page)

activity during the planarian regeneration and it was critical for the differentiation of stem cells (Pirotte et al., 2015). This study was corroborated by the work in *Xenopus* and zebrafish, which showed increased ROS activity during their tail regeneration (Gauron et al., 2013; Love et al., 2013). Inhibiting the ROS activity using the NADH oxidase inhibitors abrogated the stem cell differentiation and thus blocked regeneration implicating mitochondria as the major source of ROS generation. The primary aim of this study was to delineate the changes in mitochondrial content in the pluripotent and the committed stem cell states in planarians, which can subsequently be used as a marker to isolate PSCs.

Neoblast in planarians is characterized by the presence of a large nucleus and scanty cytoplasm. Using Hoechst, a nuclear dye, planarian cells are sorted into three classes, X1, X2, and Xins. Here, we investigated the extent of distribution of mitochondrial mass among these three populations using MTG, a mitochondrial dye. MTG accumulates in the mitochondrial matrix and covalently binds to the proteins by reacting with the free thiol groups of the cysteine residues. Its localization to the mitochondria happens regardless of the mitochondrial membrane potential and it has been used to measure the mitochondrial mass. Using MTG, we observed a low mitochondrial mass in the X1 cells compared with the Xins cells. Interestingly, MTG staining revealed a polarized distribution of mitochondria in X1 cells. It is worth noting that such subcellular localization of mitochondria has been previously implicated in cell migration and localized metabolite concentration (Al-shaabi et al., 2020), which needs to be further studied in the context of the neoblast function. MTG staining further demarcated X1 and X2 populations into MTG^{Low} and MTG^{High} subpopulations. We observed that PSCs in planaria are defined by low mitochondrial content, whereas an increase in mitochondrial content is associated with differentiation. For instance, our results indicate that the stem cells in the X1 population, which had high mitochondrial content, exhibited properties of lineage commitment such as reduced $PIWI\text{-}1^{High}$ cells and expression of transcripts associated with lineage specification. This suggests that a quantifiable change in mitochondrial mass exists within clonogenic and specialized neoblasts.

We also looked at the mitochondrial content in the X2 population, which is a mix of neoblasts and their mitotic progenies. Owing to the heterogeneity of the X2 population and low prevalence, stem cells in this gate were poorly studied. It was proposed that the clonogenic neoblasts exist in the X2 gate, nonetheless it was never experimentally validated (Molinaro and Pearson, 2018). We categorized X2 cells based on MTG intensity and characterized the subpopulations. RNA-seq analysis showed that the transcript profile of MTG^{Low} resembled stem cells and early progenitors and was reminiscent of X1 cells, while MTG^{High} was more akin to Xins. This indicated that within X2, mitochondrial content increases as the cell progresses toward differentiation. To enrich the neoblasts within the G0/G1 phase, MTG^{Low} cells in X2 were divided into two populations based on their size. Such classification of the X2 cells based on the size and MTG resulted in an enrichment of $PIWI\text{-}1^{High}$ cells in the High FSC gate. Transcriptome sequencing revealed that X2-MTG^{Low}-(LFSC) are potentially early progenitors and the X2-MTG^{Low}-(HFSC) express transcripts critical for the cell cycle progression. Together, using the size parameter and mitochondrial mass measurements, we were able to isolate neoblast and their immediate progenitors for functional characterization.

Furthermore, we also used MTO, an indicator of mitochondrial potential, to study the correlation between mitochondrial content and potential. Dual staining using MTG

Figure 6. Role of mitochondria in stem cell differentiation

(A and A′) Planarian cells stained with MTG and MTO were analyzed through flow cytometry. X1 (A) and X2 (A′) were gated for MTG^{Low} and $^{-High}$ and analyzed for MTO intensity. The bar graph indicates the median MTO intensity normalized to FCCP. Mean ± SEM, over three independent replicates. a.u., arbitrary units.

(B) Representative images of undergoing regeneration in the presence of 30 nM FCCP or DMSO (vehicle control). Scale bar, 200 μm.

(C) Representative confocal images of *piwi*-1 RNA FISH at 3 dpa in trunk fragments regenerating in 30 nM FCCP or DMSO (control). Scale bar, 100 μm.

(D) Quantitation of *piwi-1*$^{+}$ cells from the 3dpa anterior blastema region from (C). Mean ± SD, n = 7 animals.

(E) Quantitation of *piwi*-1 mRNA levels through qPCR from 3 dpa regenerating animals. Mean ± SD, n = 30 worms over three independent replicates.

(F) Schematic representing the *in vitro* assay for perturbing mitochondrial activity using FCCP.

(G) $PIWI\text{-}1^{High}$ quantitation through flow cytometry in 4N-MTG^{Low} cells treated with the indicated amount of FCCP for 48 h in culture. Mean ± SEM, over three independent replicates.

(H and I) Representative *piwi*-1 RNA FISH (H) and quantification of *piwi-1*$^{+}$ cells (I) per transplant in lethally irradiated sexual worms 8 dpt of ~2500 cells from 4N-MTG^{Low} cells cultured *in vitro* for 48 h with or without FCCP. Scale bar, 200 μm. Mean ± SEM, n = 9 worms.

(J) Percentage of worms rescued following injection of ~2500 4N-MTG^{Low} cells cultured *in vitro* for 48 h with or without FCCP. Mean ± SEM, n = 24 worms performed over three independent replicates.

*, **, n.s. represents $p < 0.05$, $p < 0.01$, and nonsignificant, respectively.

and MTO showed that the cells with low MTG were also low for MTO and vice versa, suggesting that the mitochondrial content and potential show a high degree of correlation. It is also important to note that high mitochondrial activity leads to an increased ROS production. Recent studies have shown that MTG could be influenced by ROS (Doherty and Perl, 2017), indicating that the MTG^{High} cells could show increased ROS. Notably, increased ROS is also associated with stem cell differentiation in vertebrates (Tothova et al., 2007) and invertebrates (Owusu-Ansah and Banerjee, 2009). However, a comprehensive study is warranted to understand the effect of high mitochondrial activity and subsequent increase in the ROS levels on neoblast maintenance and differentiation.

Schmidtea mediterranea exists as two strains: sexual strain, which are hermaphrodites, and asexual strain, which have underdeveloped germline tissue and undergoes reproduction by fission. Previous studies have shown that the transplantation of asexual neoblast to irradiated sexual strain transformed the sexual planaria to asexual evident from them undergoing fission (Wagner et al., 2011). Such transplantation assays in planarians provide a platform to functionally validate the pluripotency of neoblast subpopulations. Our results clearly indicate that stem cells with low mitochondrial content are more pluripotent than the cells with high mitochondrial content. This implies that low mitochondrial content is one of the characteristics of PSCs. Interestingly, our transplantation experiments also revealed the higher clonogenic capacity of the G0/G1 neoblasts. With the current state-of-the-art technologies, TSPAN-1+ cells fairs as the most potent clonogenic population. We propose that the classification of cells based on mitochondrial content would allow us to sort clonogenic neoblasts with higher purity and subsequent characterization might allow us to identify novel and rare clonogenic neoblasts, should they exist. Furthermore, the sorting methodology presented here using both nuclear and mitochondrial markers could be, in principle extended to other flatworms such as *Macrostomum lignano, Schistosoma mansoni,* and *Hoefstenia miamia* where, like planaria, only proliferating stem cells have been extensively studied (Gehrke et al., 2019; Grudniewska et al., 2016; Wang et al., 2018a).

Differentiation in mammalian pluripotent cells such as ESCs is shown to involve changes in the gene expression profile, remodeling of organelles, and their metabolic states (Dixon et al., 2015; Folmes et al., 2012; Gabut et al., 2020; Sampath et al., 2008). Further, it has been shown that during differentiation, ESCs exhibit increased mitochondrial fusion and a concurrent switch in their metabolic state from glycolytic to oxidative phosphorylation. Such metabolic change has been shown to be critical for the cell state transition (Chung et al., 2007; Tormos et al., 2011). For instance, treatment of ESCs and HSCs with electron transport chain uncouplers like FCCP resulted in defective differentiation and maintained their stemness as validated by their ability to form teratoma and functional blood reconstitution respectively (Mandal et al., 2011; Vannini et al., 2016; Varum et al., 2009).

Our study demonstrates a substantial increase in the mitochondrial mass of planarian cells during differentiation and a perturbation of mitochondrial activity led to increased sustenance of stem cells *in vitro*. Such neoblasts that are blocked for OxPhos *in vitro*, exhibited higher clonogenic capacity compared with the control cells. This indicates that for proper differentiation of stem cells, a concomitant increase in mitochondrial activity is a necessity. Further, these results suggest that the mitochondrial state has a central role in stem cell maintenance and differentiation in planarians and this biological process is evolutionarily conserved. Our findings also highlight the potential role of mitochondrial bioenergetics in regulating organismal regeneration. In addition, the increased mitochondrial content during differentiation might be a result of the higher demand for metabolites in cells priming for the differentiation. For instance, early-stage embryonic stem cell differentiation is marked by epigenetic changes and increased translation state, which requires increased levels of amino acids and cofactors such as alpha-ketoglutarate and acetyl CoA (Boland et al., 2014; Folmes et al., 2012). Also, the intermediates of the TCA cycle serve as the precursors of amino acid biosynthesis and also provide cofactors essential for epigenetic modifiers such as acetylases and demethylases. However, an in-depth study is required for a complete understanding of the exact processes that lead to increased mitochondrial mass during neoblast differentiation. In summary, the results presented here establish without ambiguity that the PSCs have lesser mitochondrial mass compared with their progenitors and this difference in the mitochondrial mass serves as an efficient marker to isolate pluripotent neoblast from their progenitors.

EXPERIMENTAL PROCEDURES

Planarian husbandry

Both sexual and asexual *Schmidtea mediterranea* strains were grown in 1x Montjuïc salts at 20°C. The worms were fed with beef liver and were starved for a minimum of 7 days before any experiments. Sexual strains receiving 6,000 rads of γ-rays were used as transplantation hosts. The transplantation hosts were maintained in gentamicin (50 μg/mL) starting 7 days prior to irradiation.

Fluorescence-activated cell sorting

Cell suspension for FACS sorting was prepared as described before (Lei et al., 2019). The worms were diced in calcium and

magnesium-free buffer with 1% BSA (CMFB) and mechanically sheared using a micropipette. The resulting single-cell suspension was stained with Hoechst 33,342 (40 μg/mL) and MTG (100 nM). X1, X2, and Xins populations were demarcated using Hoechst blue and red fluorescence. For transplantation experiments, cells stained with SiR-DNA and MTG were used.

Fluorescence *in situ* hybridization and immunostaining

In situ hybridization for assessing the *piwi-1* colonies in transplanted worms were performed as described earlier (King and Newmark, 2013). For immunostaining, an Anti-PIWI-1 antibody was raised in rabbit using the antigen NEPEGPTETDQSLS as described earlier (Guo et al., 2006). FACS sorted cells were plated in optical bottom 384 well plates (~10,000 cells per well) and stained with PIWI-1 antibody.

Cell transplantation

Bulk cell transplantation in irradiated animals was carried out as described earlier with minor modifications (Davies et al., 2017; Wang et al., 2018b). For colony expansion and long-term survival experiments, 2-day post-irradiated animals were used. The injection was carried out using an Eppendorf femtojet 4x with a pressure of 0.8 to 1.0 psi.

Statistical analysis

All statistical analyses except for transcriptome were performed using unpaired, two-tailed Student's t test and $p < 0.05$ was considered significant. Differentially expressed genes from the transcriptome analysis (two independent biological replicates) were identified using the Cuffdiff module and genes with adjusted p value <0.05 were considered as significant. For GO analysis, >2-fold upregulated, statistically significant genes were considered.

Accession numbers

The accession number for the sequencing data reported in this paper is NCBI- Sequence Read Archive (SRA): SRP272800.

SUPPLEMENTAL INFORMATION

Supplemental information can be found online at https://doi.org/10.1016/j.stemcr.2021.03.022.

AUTHOR CONTRIBUTIONS

MMH, PKV, and DP conceived and designed the study. MMH, SRS performed the experiments. VL performed the RNA-seq analysis. KL, PKV, and DP supervised the entire study. MMH and DP wrote the manuscript with inputs from all the authors. PKV and DP acquired funding.

CONFLICTS OF INTEREST

The authors declare no competing or financial interests.

ACKNOWLEDGMENTS

The authors thank Prof. Alejandro Sanchez Alvarado for hosting and training MMH on transplantation experiments in his laboratory at the Stower's Institute for Medical Research. We thank Prof. Apurva Sarin for the valuable inputs and critical comments on the manuscript. We acknowledge all the Palakodeti lab members especially Srikar Krishna for the critical inputs. The authors thank Central Imaging and Flow Cytometry Facility (CIFF-BLiSc campus) and NGS facility. MMH is supported by DBT JRF, VL is supported by CSIR-SRF, SRS thanks NCBS-PhD fellowship for graduate studies. The work was funded by DST Swarnajayanti Fellowship (DST/SJF/LSA-02/2015-16) awarded to DP and PKV acknowledges Department of Biotechnology, inStem for core funding.Conflicts of interest
The authors declare no competing or financial interests.

Received: July 29, 2020
Revised: March 18, 2021
Accepted: March 18, 2021
Published: April 15, 2021

REFERENCES

Alshaabi, H., Heininger, M., and Cunniff, B. (2020). Dynamic regulation of subcellular mitochondrial position for localized metabolite levels. J. Biochem. *167*, 109–117.

Boland, M.J., Nazor, K.L., and Loring, J.F. (2014). Epigenetic regulation of pluripotency and differentiation. Circ. Res. *115*, 311–324.

Chung, S., Dzeja, P.P., Faustino, R.S., Perez-Terzic, C., Behfar, A., and Terzic, A. (2007). Mitochondrial oxidative metabolism is required for the cardiac differentiation of stem cells. Nat. Clin. Pract. Cardiovasc. Med. *4*, S60–S67.

Davies, E.L., Lei, K., Seidel, C.W., Kroesen, A.E., Mckinney, S.A., Guo, L., Robb, S.M.C., Ross, E.J., Gotting, K., and Sa, A. (2017). Embryonic origin of adult stem cells required for tissue homeostasis and regeneration. Elife *6*, e21052.

Dixon, J.R., Jung, I., Selvaraj, S., Shen, Y., Antosiewicz-Bourget, J.E., Lee, A.Y., Ye, Z., Kim, A., Rajagopal, N., Xie, W., et al. (2015). Chromatin architecture reorganization during stem cell differentiation. Nature *518*, 331–336.

Doherty, E., and Perl, A. (2017). Measurement of mitochondrial mass by flow cytometry during oxidative stress. React. Oxyg. Species *4*, 275–283.

Eisenhoffer, G.T., Kang, H., and Alvarado, A.S. (2008). Molecular analysis of stem cells and their descendants during cell turnover and regeneration in the planarian *Schmidtea mediterranea*. Cell Stem Cell *3*, 327–339.

Fincher, C.T., Wurtzel, O., de Hoog, T., Kravarik, K.M., and Reddien, P.W. (2018). Cell type transcriptome atlas for the planarian *Schmidtea mediterranea*. Science *360*, eaaq1736.

Folmes, C.D.L., Dzeja, P.P., Nelson, T.J., and Terzic, A. (2012). Metabolic plasticity in stem cell homeostasis and differentiation. Cell Stem Cell *11*, 596–606.

Gabut, M., Bourdelais, F., and Durand, S. (2020). Ribosome and translational control in stem cells. Cells *9*, 497.

Gauron, C., Rampon, C., Bouzaffour, M., Ipendey, E., Teillon, J., Volovitch, M., and Vriz, S. (2013). Sustained production of ROS triggers compensatory proliferation and is required for regeneration to proceed. Sci. Rep. *3*, 1–9.

Gehrke, A.R., Neverett, E., Luo, Y.J., Brandt, A., Ricci, L., Hulett, R.E., Gompers, A., Graham Ruby, J., Rokhsar, D.S., Reddien, P.W., et al. (2019). Acoel genome reveals the regulatory landscape of whole-body regeneration. Science *363*, eaau6173.

Grudniewska, M., Mouton, S., Simanov, D., Beltman, F., Grelling, M., De Mulder, K., Arindrarto, W., Weissert, P.M., van der Elst, S., and Berezikov, E. (2016). Transcriptional signatures of somatic neoblasts and germline cells in *Macrostomum lignano*. Elife *5*, e20607.

Guo, T., Peters, A.H.F.M., and Newmark, P.A. (2006). A bruno-like gene is required for stem cell maintenance in planarians. Dev. Cell *11*, 159–169.

Hay, E.D., and Coward, S.J. (1975). Fine structure studies on the planarian, Dugesia. I. Nature of the "neoblast" and other cell types in noninjured worms. J. Ultrasructure Res. *50*, 1–21.

Hayashi, T., Asami, M., Higuchi, S., Shibata, N., and Agata, K. (2006). Isolation of planarian X-ray-sensitive stem cells by fluorescence-activated cell sorting. Dev. Growth Differ. *48*, 371–380.

King, R.S., and Newmark, P.A. (2013). In situ hybridization protocol for enhanced detection of gene expression in the planarian *Schmidtea mediterranea*. BMC Dev. Biol. *13*, 1–16.

Lei, K., McKinney, S.A., Ross, E.J., Lee, H.-C., and Alvarado, A.S. (2019). Cultured pluripotent planarian stem cells retain potency and express proteins from exogenously introduced mRNAs. BioRxiv, 573725.

Love, N.R., Chen, Y., Ishibashi, S., Kritsiligkou, P., Lea, R., Koh, Y., Gallop, J.L., Dorey, K., and Amaya, E. (2013). Amputation-induced reactive oxygen species are required for successful Xenopus tadpole tail regeneration. Nat. Cell Biol. *15*, 222–228.

Mandal, S., Lindgren, A.G., Srivastava, A.S., Clark, A.T., and Banerjee, U. (2011). Mitochondrial function controls proliferation and early differentiation potential of embryonic stem cells. Stem Cells *29*, 486–495.

Molinaro, A.M., and Pearson, B.J. (2016). In silico lineage tracing through single cell transcriptomics identifies a neural stem cell population in planarians. Genome Biol. *17*, 1–17.

Molinaro, A.M., and Pearson, B.J. (2018). Myths vs. FACS: what do we know about planarian stem cell lineages? Int. J. Dev. Biol. *62*, 527–535.

Morita, M., Best, J.B., and Noel, J. (1969). Electron microscopic studies of planarian regeneration. I. Fine structure of neoblasts in *Dugesia dorotocephala*. J. Ultrasructure Res. *27*, 7–23.

Newmark, P.A., and Sánchez Alvarado, A. (2000). Bromodeoxyuridine specifically labels the regenerative stem cells of planarians. Dev. Biol. *220*, 142–153.

Owusu-Ansah, E., and Banerjee, U. (2009). Reactive oxygen species prime *Drosophila* haematopoietic progenitors for differentiation. Nature *461*, 537–541.

Pedersen, K.J. (1959). Cytological studies on the planarian neoblast. Z. Für Zellforsch. Mikroskopische Anat. *50*, 799–817.

Pirotte, N., Stevens, A.S., Fraguas, S., Plusquin, M., Van Roten, A., Van Belleghem, F., Paesen, R., Ameloot, M., Cebrià, F., Artois, T., et al. (2015). Reactive oxygen species in planarian regeneration: an upstream necessity for correct patterning and brain formation. Oxid. Med. Cell. Longev. *2015*, 392476.

Plass, M., Solana, J., Alexander Wolf, F., Ayoub, S., Misios, A., Glažar, P., Obermayer, B., Theis, F.J., Kocks, C., and Rajewsky, N. (2018). Cell type atlas and lineage tree of a whole complex animal by single-cell transcriptomics. Science *360*, eaaq1723.

Reddien, P.W. (2018). The cellular and molecular basis for planarian regeneration. Cell *175*, 327–345.

Reddien, P.W., and Alvarado, A.S. (2004). Fundamentals of planarian regeneration. Annu. Rev. Cell Dev. Biol. *20*, 725–757.

Reddien, P.W., Oviedo, N.J., Jennings, J.R., Jenkin, J.C., and Sánchez Alvarado, A. (2005). SMEDWI-2 is a PIWI-like protein that regulates planarian stem cells. Science *310*, 1327–1330.

Romero-Moya, D., Bueno, C., Montes, R., Navarro-Montero, O., Iborra, F.J., López, L.C., Martin, M., and Menendez, P. (2013). Cord blood-derived CD34+ hematopoietic cells with low mitochondrial mass are enriched in hematopoietic repopulating stem cell function. Haematologica *98*, 1022–1029.

Sampath, P., Pritchard, D.K., Pabon, L., Reinecke, H., Schwartz, S.M., Morris, D.R., and Murry, C.E. (2008). A hierarchical network controls protein translation during murine embryonic stem cell self-renewal and differentiation. Cell Stem Cell *2*, 448–460.

Sukumar, M., Liu, J., Mehta, G.U., Patel, S.J., Roychoudhuri, R., Crompton, J.G., Klebanoff, C.A., Ji, Y., Li, P., Yu, Z., et al. (2016). Mitochondrial membrane potential identifies cells with enhanced stemness for cellular therapy. Cell Metab. *23*, 63–76.

Tormos, K.V., Anso, E., Hamanaka, R.B., Eisenbart, J., Joseph, J., Kalyanaraman, B., and Chandel, N.S. (2011). Mitochondrial complex III ROS regulate adipocyte differentiation. Cell Metab. *14*, 537–544.

Tothova, Z., Kollipara, R., Huntly, B.J., Lee, B.H., Castrillon, D.H., Cullen, D.E., McDowell, E.P., Lazo-Kallanian, S., Williams, I.R., Sears, C., et al. (2007). FoxOs are critical mediators of hematopoietic stem cell resistance to physiologic oxidative stress. Cell *128*, 325–339.

Trapnell, C., Hendrickson, D.G., Sauvageau, M., Goff, L., Rinn, J.L., and Pachter, L. (2013). Differential analysis of gene regulation at transcript resolution with RNA-seq. Nat. Biotechnol. *31*, 46–53.

Vannini, N., Girotra, M., Naveiras, O., Nikitin, G., Campos, V., Giger, S., Roch, A., Auwerx, J., and Lutolf, M.P. (2016). Specification of haematopoietic stem cell fate via modulation of mitochondrial activity. Nat. Commun. *7*, 1–9.

Varum, S., Momčilović, O., Castro, C., Ben-Yehudah, A., Ramalho-Santos, J., and Navara, C.S. (2009). Enhancement of human embryonic stem cell pluripotency through inhibition of the mitochondrial respiratory chain. Stem Cell Res. *3*, 142–156.

Wagner, D.E., Wang, I.E., and Reddien, P.W. (2011). Clonogenic neoblasts are pluripotent adult stem cells that underlie planarian regeneration. Science *332*, 811–816.

Wagner, D.E., Ho, J.J., and Reddien, P.W. (2012). Genetic regulators of a pluripotent adult stem cell system in planarians identified by RNAi and clonal analysis. Cell Stem Cell *10*, 299–311.

Wang, B., Lee, J., Li, P., Saberi, A., Yang, H., Liu, C., Zhao, M., and Newmark, P.A. (2018a). Stem cell heterogeneity drives the parasitic life cycle of *Schistosoma mansoni*. Elife *7*, e35449.

Wang, I.E., Wagner, D.E., and Reddien, P.W. (2018b). Clonal analysis of planarian stem cells by subtotal irradiation and single-cell transplantation. In Planarian Regeneration (Humana Press), pp. 479–495.

Van Wolfswinkel, J.C., Wagner, D.E., and Reddien, P.W. (2014). Single-cell analysis reveals functionally distinct classes within the planarian stem cell compartment. Cell Stem Cell *15*, 326–339.

Xu, X., Duan, S., Yi, F., Ocampo, A., Liu, G.H., and Izpisua Belmonte, J.C. (2013). Mitochondrial regulation in pluripotent stem cells. Cell Metab. *18*, 325–332.

Zeng, A., Li, H., Guo, L., Gao, X., McKinney, S., Wang, Y., Yu, Z., Park, J., Semerad, C., Ross, E., et al. (2018). Prospectively isolated Tetraspanin+Neoblasts are adult pluripotent stem cells underlying planaria regeneration. Cell, 1593–1608.

Stem Cell Reports, Volume *16*

Supplemental Information

Mitochondrial state determines functionally divergent stem cell population in planaria

Mohamed Mohamed Haroon, Vairavan Lakshmanan, Souradeep R. Sarkar, Kai Lei, Praveen Kumar Vemula, and Dasaradhi Palakodeti

Figure S1:

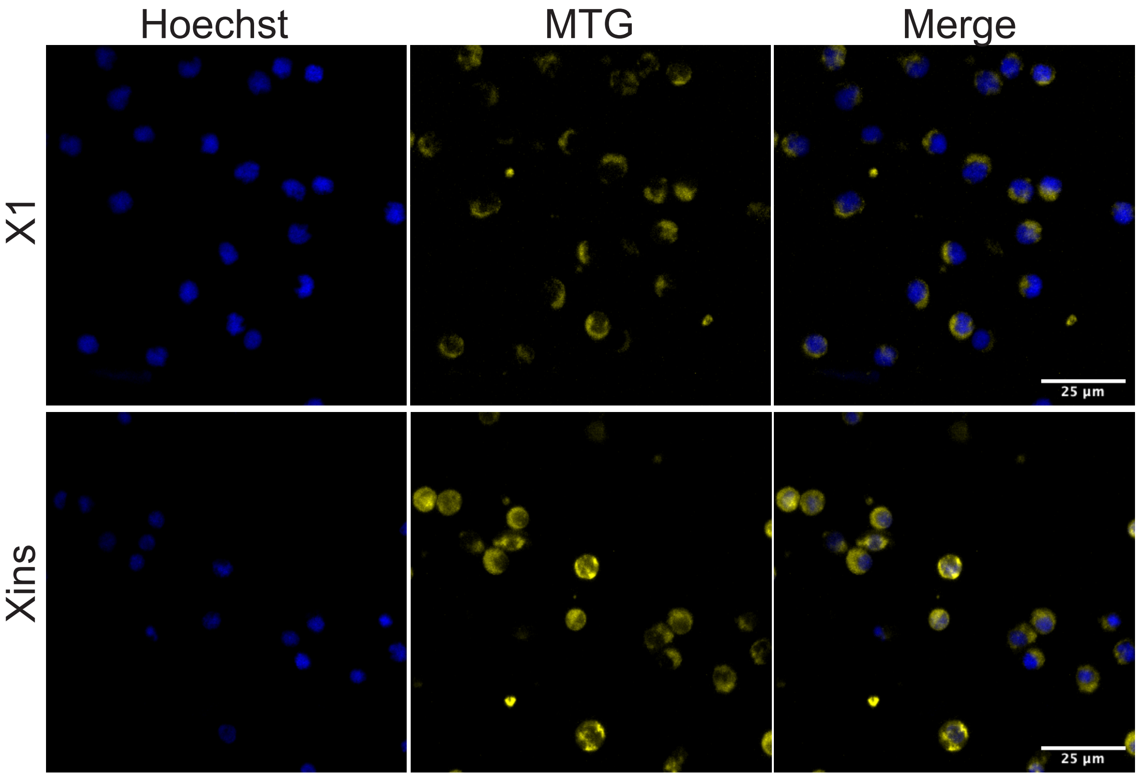

Figure S2:

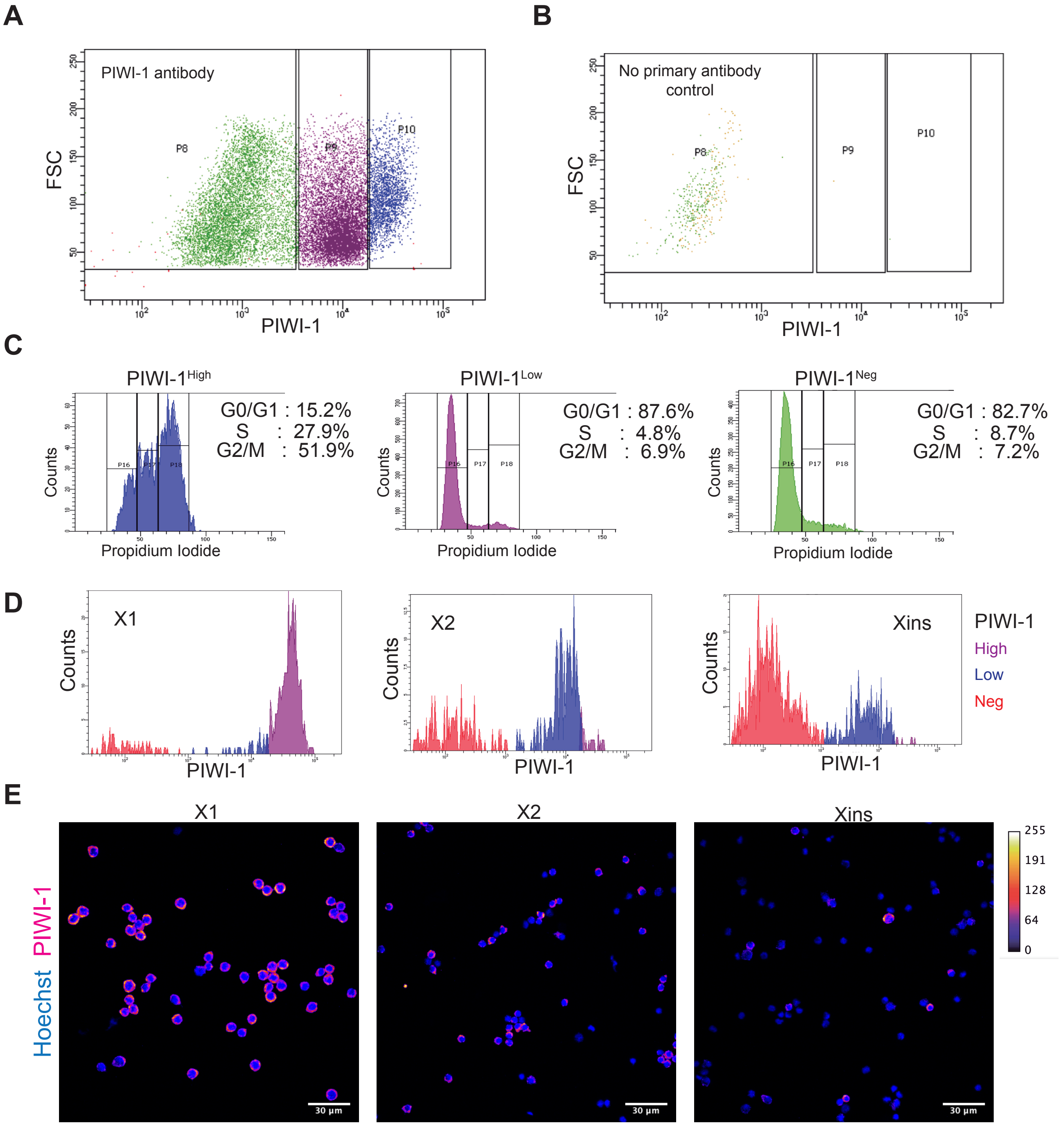
A
PIWI-1 antibody
FSC
PIWI-1
P8
P9
P10
B
No primary antibody control
FSC
PIWI-1
P8
P9
P10
C
PIWI-1High
G0/G1 : 15.2%
S : 27.9%
G2/M : 51.9%
Counts
Propidium Iodide
PIWI-1Low
G0/G1 : 87.6%
S : 4.8%
G2/M : 6.9%
Counts
Propidium Iodide
PIWI-1Neg
G0/G1 : 82.7%
S : 8.7%
G2/M : 7.2%
Counts
Propidium Iodide
P16
P17
P18
D
X1
Counts
PIWI-1
X2
Counts
PIWI-1
Xins
Counts
PIWI-1
PIWI-1
High
Low
Neg
E
X1
X2
Xins
Hoechst PIWI-1
30 µm
255
191
128
64
0

Figure S3:

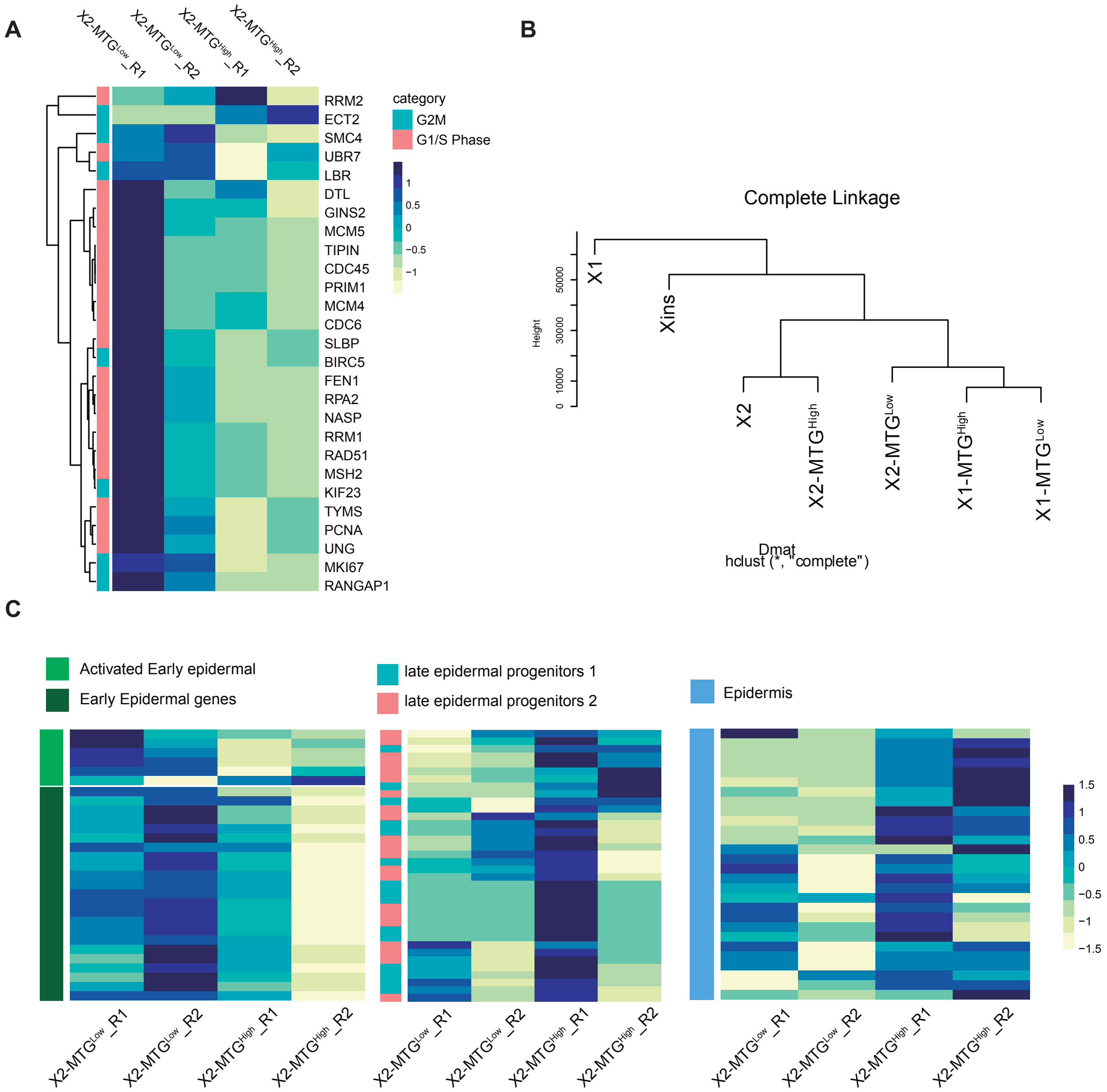
A
X2-MTGLow_R1
X2-MTGLow_R2
X2-MTGHigh_R1
X2-MTGHigh_R2
RRM2
ECT2
SMC4
UBR7
LBR
DTL
GINS2
MCM5
TIPIN
CDC45
PRIM1
MCM4
CDC6
SLBP
BIRC5
FEN1
RPA2
NASP
RRM1
RAD51
MSH2
KIF23
TYMS
PCNA
UNG
MKI67
RANGAP1
category
G2M
G1/S Phase
1
0.5
0
−0.5
−1
B
Complete Linkage
Height
0
10000
30000
50000
X1
Xins
X2
X2-MTGHigh
X2-MTGLow
X1-MTGHigh
X1-MTGLow
Dmat
hclust (*, "complete")
C
Activated Early epidermal
Early Epidermal genes
late epidermal progenitors 1
late epidermal progenitors 2
Epidermis
1.5
1
0.5
0
−0.5
−1
−1.5
X2-MTGLow_R1
X2-MTGLow_R2
X2-MTGHigh_R1
X2-MTGHigh_R2

Figure S4:

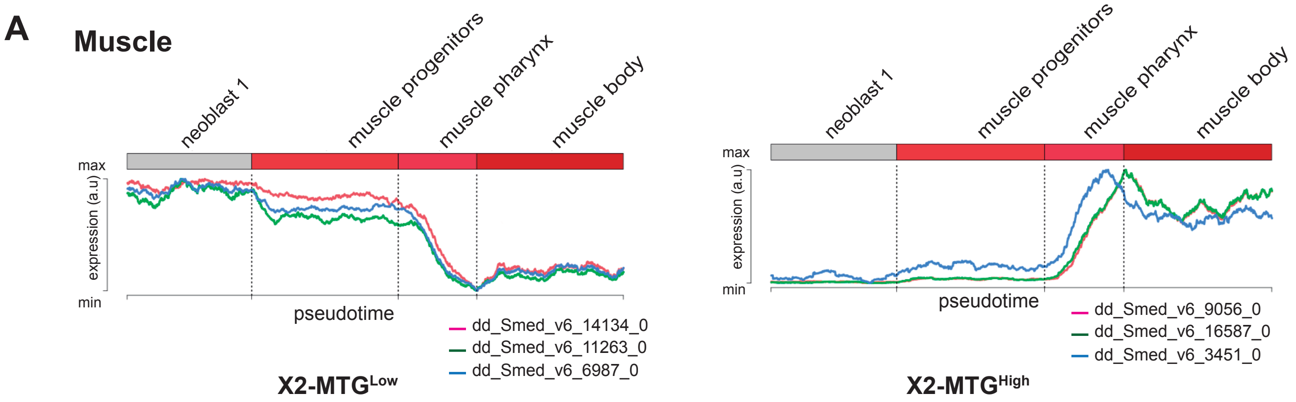
A
Muscle
neoblast 1
muscle progenitors
muscle pharynx
muscle body
max
min
expression (a.u)
pseudotime
dd_Smed_v6_14134_0
dd_Smed_v6_11263_0
dd_Smed_v6_6987_0
X2-MTGLow
dd_Smed_v6_9056_0
dd_Smed_v6_16587_0
dd_Smed_v6_3451_0
X2-MTGHigh

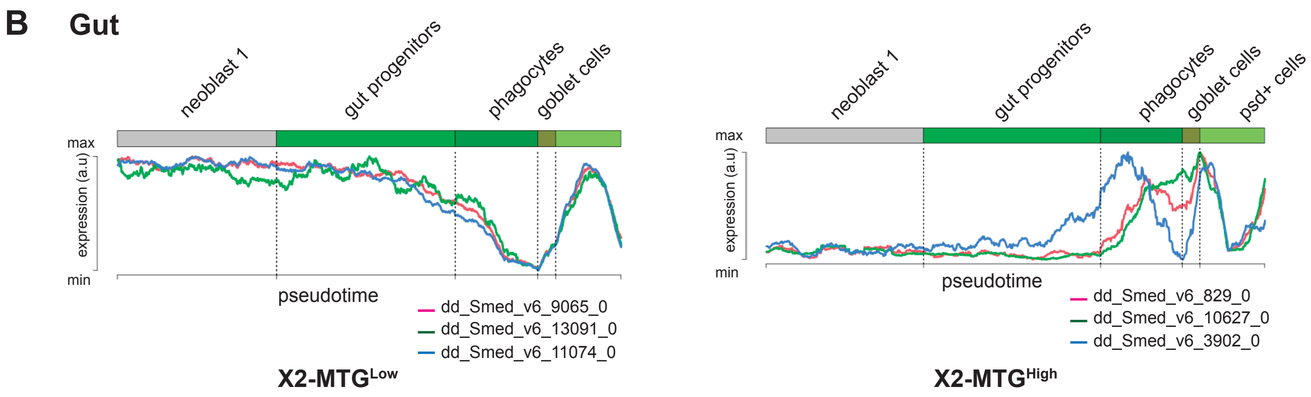
B
Gut
neoblast 1
gut progenitors
phagocytes
goblet cells
psd+ cells
max
min
expression (a.u)
pseudotime
dd_Smed_v6_9065_0
dd_Smed_v6_13091_0
dd_Smed_v6_11074_0
X2-MTGLow
dd_Smed_v6_829_0
dd_Smed_v6_10627_0
dd_Smed_v6_3902_0
X2-MTGHigh

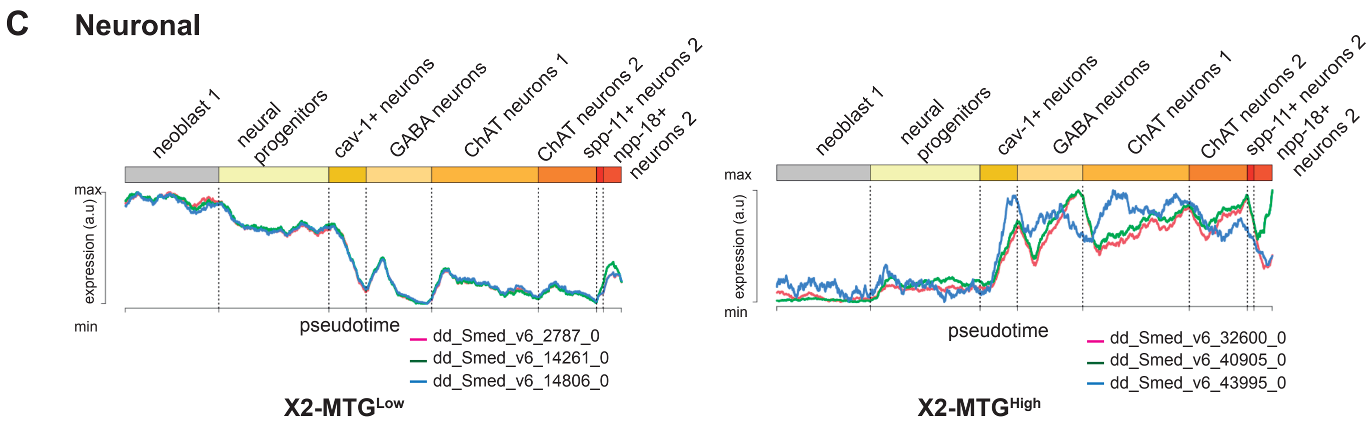
C
Neuronal
neoblast 1
neural progenitors
cav-1+ neurons
GABA neurons
ChAT neurons 1
ChAT neurons 2
spp-11+ neurons 2
npp-18+ neurons 2
max
min
expression (a.u)
pseudotime
dd_Smed_v6_2787_0
dd_Smed_v6_14261_0
dd_Smed_v6_14806_0
X2-MTGLow
dd_Smed_v6_32600_0
dd_Smed_v6_40905_0
dd_Smed_v6_43995_0
X2-MTGHigh

Figure S5:

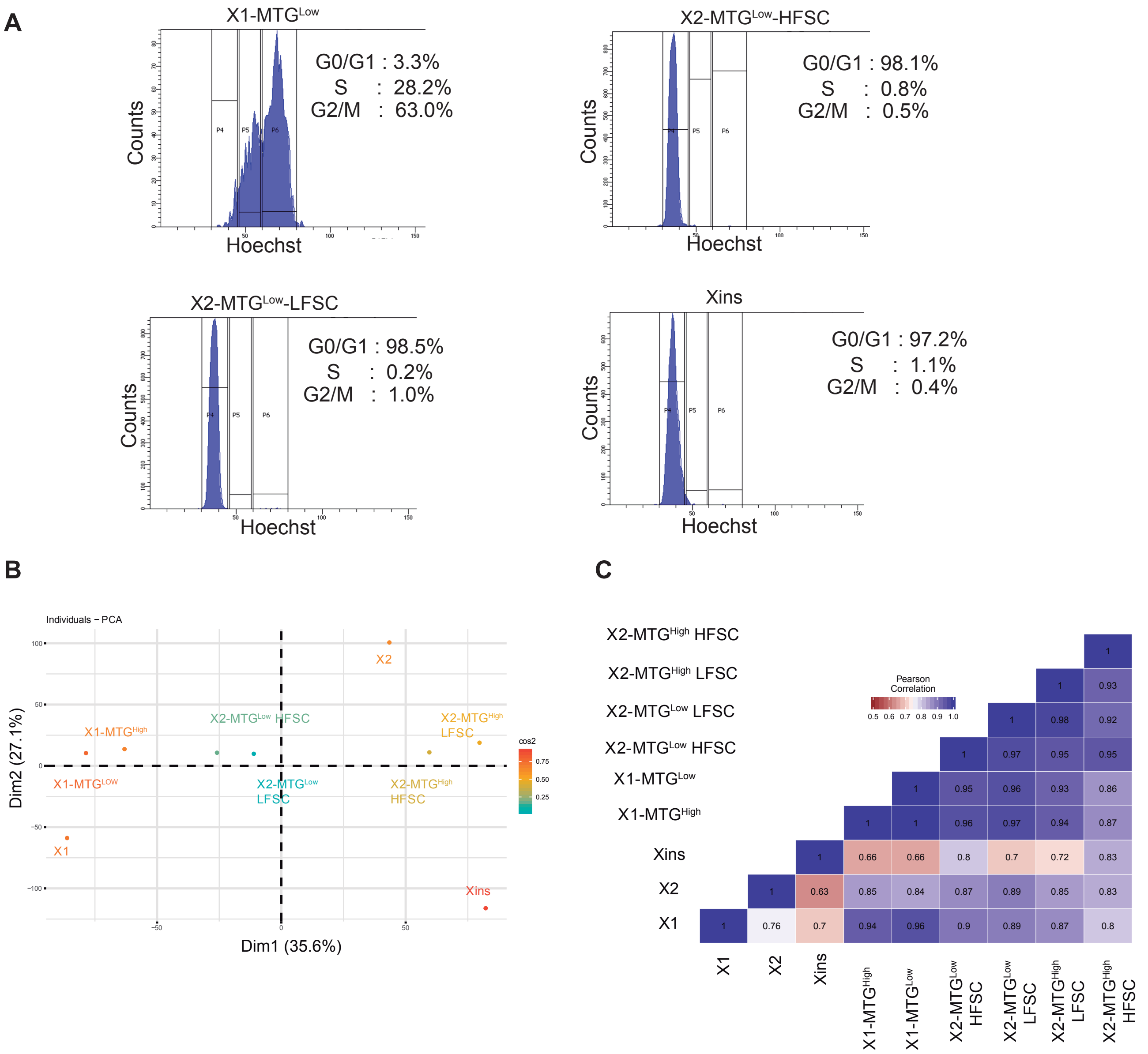
A
X1-MTGLow
G0/G1 : 3.3%
S : 28.2%
G2/M : 63.0%
X2-MTGLow-HFSC
G0/G1 : 98.1%
S : 0.8%
G2/M : 0.5%
X2-MTGLow-LFSC
G0/G1 : 98.5%
S : 0.2%
G2/M : 1.0%
Xins
G0/G1 : 97.2%
S : 1.1%
G2/M : 0.4%
Counts
Hoechst
B
Individuals – PCA
Dim1 (35.6%)
Dim2 (27.1%)
X1
X2
Xins
X1-MTGHigh
X1-MTGLOW
X2-MTGLow HFSC
X2-MTGLow LFSC
X2-MTGHigh LFSC
X2-MTGHigh HFSC
cos2
C
Pearson Correlation
X2-MTGHigh HFSC
X2-MTGHigh LFSC
X2-MTGLow LFSC
X2-MTGLow HFSC
X1-MTGLow
X1-MTGHigh
Xins
X2
X1

Figure S6:

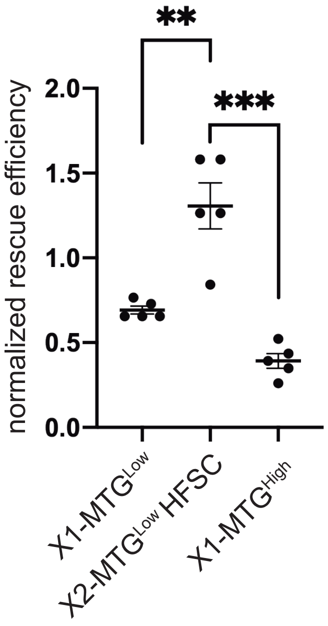

Figure S7:

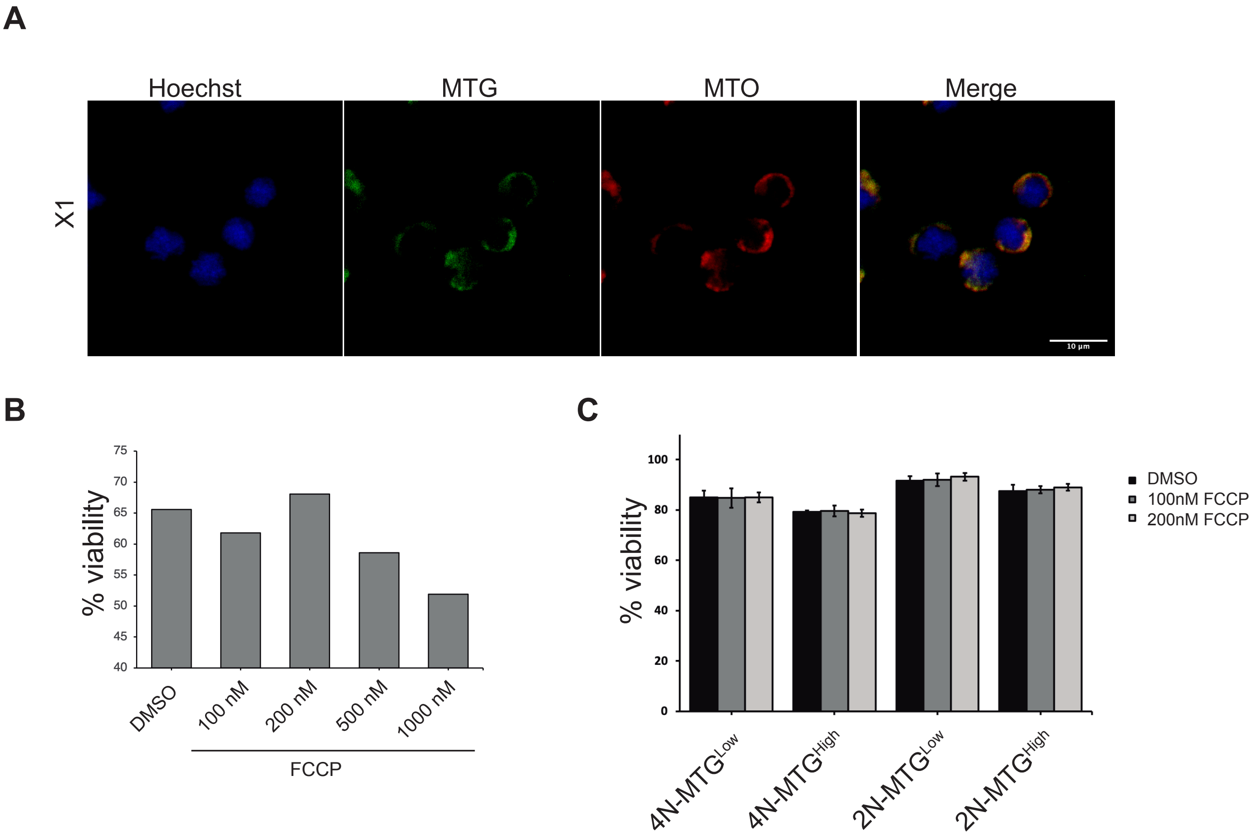

Supplemental figure legends:

Supplementary Figure S1 (related to Figure 1). Representative low magnification images of MTG fluorescence in sorted live X1 and Xins cells. Scale bar 25 μm.

Supplementary Figure S2. PIWI-1 Antibody staining validation (related to Figure 2,3)

(A) Dot plot showing PIWI-1 staining in planarian cells. PIWI-1 High (blue), low (pink), and negative (green).

(B) Dot plot of negative control cells- only secondary antibody was added without any primary antibody.

(C) Cell cycle stages of corresponding PIWI-1 population analyzed by flow cytometry. Percentage of cells in G0/G1, S, and G2/M for the indicated PIWI-1 population is given.

(D and E) PIWI-1 staining in sorted X1, X2, and Xins cells analyzed by flow cytometry **(D)** or confocal microscopy **(E)** scale bar: 30 μm. PIWI-1 signal is shown as Fire LUT (ImageJ)

Supplementary Figure S3. Transcriptome analysis of X2-MTG^{Low} and MTG^{High} population (related to Figure 4)

(A) Heat map showing the expression of key cell cycle genes in X2-MTG^{Low} and MTG^{High} cells.

(B) Linkage mapping of X2-MTG populations with X1-MTG and X1, X2, and Xins populations.

(C) Heat map showing expression of key genes implicated in early epidermal progenitors, late progenitors and epidermis in X2-MTG^{Low} and MTG^{High} cells. R1 and R2 represent biological replicates.

Supplementary Figure S4. Pseudotime analysis of transcripts X2-MTG^{Low} and MTG^{High} population (related to Figure 4)

(A-C) Representative transcripts enriched in either X2-MTG^{Low} (left) or X2-MTG^{High} (right) were used to analyze the pseudotime expression from neoblasts to terminally differentiated cells using an available single-cell database. Three representative transcripts related to the muscle **(A)**, gut **(B),** and neuronal **(C)** lineages are shown

Supplementary Figure S4. Cell cycle and transcriptome analysis of X2-MTG^{Low} High and Low FSC populations (related to Figure 4)

(A) Representative flow cytometry histograms indicating cell cycle stages of X1, Xins, and X2-MTG subpopulations which were sorted, fixed, and stained with Hoechst.

(B) Principal component analysis comparing all the MTG subpopulations with X1, X2, and Xins

(C) Pearson's correlation heatmap of the MTG populations compared to X1, X2, and Xins

Supplementary Figure S6. Normalized rescue of MTG subpopulations (related to Figure 5)

(A) Normalized rescue efficiency calculated by dividing the rescue efficiency by the percentage of PIWI-1^{High} cells obtained from Figures 2B and 3B.

Supplementary Figure S7 (related to figure 6)

(A) Representative confocal micrograph showing colocalization of MTG and MTO staining in X1 cells. Scale bar 10 μm.

(B) Cell viability obtained by propidium iodide exclusion of X1(fs) cells treated with the indicated concentration of FCCP or DMSO for 48 hours *in vitro*. Concentrations above 500 nM FCCP were significantly toxic.

(C) Cell viability of indicated X1 and X2 MTG subpopulations after 48 hours of *in vitro* culture in either 100nM or 200nM FCCP. Error bar indicated standard deviation from three independent replicates.

Supplemental experimental procedures

FACS sorting of cells using Hoechst 33342 and SiR-DNA

Cell suspension was prepared as described before (Lei et al., 2019). In brief, planarians were chopped in calcium and magnesium-free buffer (Wang et al., 2018) with 1% bovine serum albumin (CMFB) (Sigma BSA A2153) and transferred to a 50 mL centrifuge tube using a wide-bore pipette tip. The diced fragments are mechanically sheared by pipetting until all the fragments are dissociated. The resulting cell suspension is strained through a 40 μm cell strainer (BD Falcon, 352340) and centrifuged at 290 g for 10 mins. Then, the supernatant was removed and isotonic planaria media (IPM) (Lei et al., 2019) with 10% FBS is added. To this solution, 40 μg/mL Hoechst 33342 (Invitrogen, H3570) was added and the staining is carried out at room temperature (RT) for 50 mins with intermittent mixing. To the same suspension, MitoTracker Green FM (Invitrogen, M7514) was added to a final concentration of 100 nM and stained for further 20 mins. Post staining, the suspension was again centrifuged (290 g, 10 mins) and the supernatant was discarded. The resulting cells were resuspended in IPM 10% FBS with 1 μg/mL propidium iodide (Sigma, P4864) and immediately analyzed through flow cytometry. A similar protocol was used for SiR-DNA (1 μM, Cytoskeleton, Inc. CY-SC007) staining, except for propidium iodide, DAPI (1 mg/mL) (Sigma D9542) was used to discriminate live/dead cells. X1 and X2 gates were set as reported previously (Zeng et al., 2018). A wider gate for X2 was preferred in order to accommodate late progenitors. For size-based sorting of MTG populations, forward scatter voltage was adjusted such that X1 and X2 populations are well separated in the FSC axis. First, Hoechst stained cells were used to define the forward and side scatter for X1(FS) and X2(FS) gates. Within the X1(FS) gate 4N SiR-DNA population was selected (X1 equivalent) and within the X2(FS) gate 2N cells were

selected (X2 equivalent) in order to reduce cross-contamination. For Verapamil (Cytoskeleton, Inc. CY-SC007) treatment, the planarian cell suspension was treated with Hoechst and MTG in the presence of indicated concentrations of Verapamil for 1 hour. The cells were sorted using 100 μm tip and 20 psi sheath pressure in BD FACS ARIA III or Aria Fusion sorter.

Quantitation of mitochondrial membrane potential

Planarian single-cell suspensions prepared as described previously was treated with Hoechst for 40 mins and simultaneously stained with MTG and MTO (100 nM each) for another 20 mins and analyzed through flow cytometry (BD FACS Fortessa). As a control, cells were also treated with 20 μM of FCCP for 10 mins prior to the addition of MTO. Median MTO intensity of FCCP treated cells was subtracted from the non-FCCP treated population to get the true membrane potential values.

Antibody staining of FACS sorted cells

Anti-PIWI-1 antibody was raised in rabbit using the antigen NEPEGPTETDQSLS as described earlier (Guo et al., 2006). FACS sorted cells were centrifuged (300 g, 10 mins) and resuspended in IPM 5% FBS and 50 μL (~10,000 cells) was added in 384 well plates (Thermo Scientific Nunc, 142761). The plates were centrifuged and the cells were fixed in 50 μL, 4% PFA in PBSTx (0.1% Triton X100) for 10 minutes. Post incubation, 50 μL was removed, and again 50 μL of fresh PFA fixative was added and incubated for further 10 mins. Then, the entire solution was carefully removed and washed with PBSTx and permeabilized with ice-cold 90% methanol for 10 minutes. After washing twice with PBSTx, blocking was carried out for 30 minutes at RT with 10% horse serum in PBSTx. Anti-PIWI-1 antibody was diluted to 1:300 in blocking buffer was added and incubated overnight at 4 degrees Celsius. After washing with PBSTx, secondary antibody was added (1:1000) (Anti-rabbit IgG-Alexa Fluor 680, Invitrogen, A10043) for one hour at RT. The cells were further washed and stained with Hoechst.

Cell cycle analysis:

Freshly sorted cells were centrifuged and resuspended in fixative (4% PFA in PBSTx) for 20 minutes at RT. The cells were washed twice with PBSTx and stained with Hoechst 33342 for 2 hours at RT with intermittent mixing and analyzed through flow cytometry.

Neoblast culture *in vitro*

Culturing of neoblast cells was performed as described previously (Lei et al., 2019). Briefly, ~120,000 FACS sorted 4N-MTG^{Low} cells were centrifuged and resuspended in KnockOut^{TM} DMEM (Thermo Scientific 10829018) with 5% fetal bovine serum and plated in 24 well plate pretreated with Poly-D-Lysine (50 µg/mL, BD Biosciences, 354210). The media was supplemented with the indicated concentration of FCCP or vehicle alone (DMSO) and cultured for 48 hours at 22 degrees Celsius and 5% CO_2. Post 48 hours, PIWI-1 antibody staining was performed as described earlier.

Cell viability assay:

Propidium iodide exclusion assay was performed in order to assess the viability of cultured cells. First, X1(fs) cells were sorted and culture as described above in the presence of either DMSO (vehicle control) or various FCCP concentrations (100nM- 1000nM). After 48 hours, cells were washed and media containing propidium iodide (2 µg/mL) was added. The cells were immediately analyzed through flow cytometry. The concentrations 100 and 200 nM which were found not to be toxic were used for further experiments. The viability of various MTG populations upon treatment with 100 and 200 nM was assessed separately as described above.

FCCP treatment in regenerating planarians:

For FCCP treatment *in vivo*, planarians were amputated and ~4 hours post-amputation, the trunk fragments were treated with FCCP or DMSO in 1x Montjuïc salt (vehicle control). A fresh solution of FCCP or DMSO was replaced every day.

Fluorescent in situ hybridization

In situ hybridization for assessing the *piwi-1* colonies in transplanted worms and regenerating worms treated with FCCP were performed as described earlier with minor modifications (King and Newmark, 2013). RNA riboprobe labelled with Digoxigenin against *piwi-1* was prepared by DIG RNA Labeling Mix (Sigma, 11277073910). 8 days post-transplantation, worms were killed in 5% n-acetyl cysteine in PBS and fixed in 4% formaldehyde. The worms were serially dehydrated in methanol and stored at -20 degrees overnight. The worms were then rehydrated and bleached by H_2O_2 in formamide under white light. The bleached worms were permeabilized using Proteinase K (2 µg/mL), followed by fixation using 4% formaldehyde in PBSTx (0.5% Triton X-100). The worms were then transferred to prehybridization solution for 2 hours at 56 deg. Following this, hybridization was performed for 18-20 hours at 56 deg. Post hybridization, the worms were washed with 2X and 0.2X SSC buffer, followed by TNTx (0.1 M tris, 0.15 M NaCl, 0.3% Triton X-100, pH 7.5). The worms were then blocked in 5% horse serum, 0.5% Roche western blocking reagent in TNTx. Anti-digoxigenin POD antibody (1:1000) was added and incubated overnight at 4 degrees. The worms were then washed and developed using CY-3 tyramide signal amplification. The worms were counterstained with Hoechst and mounted using Scale A2.

Microscopy

Olympus FV3000 and FV 1000 confocal microscopes were used to image *piwi-1* FISH and immunostaining. The images were analyzed using imageJ (https://imagej.nih.gov/ij).

Micrographs of live worms recovering from irradiation experiments and FCCP treated regenerating worms were acquired using Olympus SZ-16 stereo microscope.

RNA extraction

Total RNA was extracted from sorted cells and FCCP treated worms using Trizol™ method (Invitrogen, 15596026). Cells were centrifuged and 0.5 mL of Trizol™ was added for ~100,000 cells. For regenerating 3dpa animals, 10 animals were dissolved in 1 mL of Trizol™. RNA extraction was carried out as per the manufacturer's protocol. Total RNA extracted was quantified using Qubit RNA HS assay kit (Invitrogen Q32852) method and the RNA quality and integrity were verified using Bioanalyzer.

Transcriptomic profiling of mitochondrial populations

Transcriptome profiling for 6 population (X1-MTG^{Low}, X1-MTG^{High}, X2-MTG^{Low} HFSC and LFSC cells, X2-MTG^{High} HFSC and LFSC cells) were performed. Transcriptome library was prepared using NEBNext® Ultra™ II Directional RNA Library Prep with Sample Purification Beads (Catalog no-E7765L) kit and sequenced in Illumina HiSeq 2500 machine. All the samples were sequenced (as single-end) in biological replicates. Approximately 25 to 40 million reads were sequenced for every sample. These reads were adapter trimmed using Trimmomatic (Bolger et al., 2014) and mapped to rRNA and other contamination databases. Reads that did not align with these databases were taken for further analysis. We used reference-based transcriptome assembly algorithms Hisat2 v2.1.0 (Kim et al., 2015); Cufflinks v2.2.1 (Trapnell et al., 2010) and Cuffdiff v2.2.1 (Trapnell et al., 2013) to identify differentially expressed transcripts. We used Hisat2 (-q -p 8 --min-intronlen 50 --max-intronlen 250000 --dta-cufflinks --new-summary --summary-file) to align the reads back to dd_Smes_G4 (Grohme et al., 2018) assembly of *Schmidtea mediterranea* genome. Around 52-70% of reads were mapped to dd_Smes_G4 genome. We used samtools to obtain sorted bam files. The mapped reads were assembled using Cufflinks (-p -o -b -u -N --total-hits-norm -G)

with the most recent and well-annotated SMEST transcriptome as reference (http://planmine.mpi-cbg.de/planmine/assemblyReport.do?assemblyId=SMEST.1_report.html). We used cuffmerge to merge the gene list across different conditions. We identified differentially expressed genes using Cuffdiff module (-p -o ./ -b -u -N --total-hits-norm -L) and considered genes with adjusted p-value <0.05 as significance cut-off. Genes with significant p-value and at least two-fold up/downregulation were considered for GO and pathway analysis. Along with these mitochondrial based sub-populations we have used RNASeq data for the total X1, X2 and Xins population from SRA. We downloaded X1 and Xins transcriptome data from PRJNA296017 (four replicates each) (Tu et al., 2015) and X2 from PRJNA167022 (two replicates) (Labbé et al., 2012). We analysed these data as described above and used them for dimensional reduction analysis (PCA, clustering). Like X1 cells, to obtain only MTG low and high populations for X2, we considered HFSC and LFSC cells of the same MTG levels (either low or high) as replicates.

One of the replicates for the X2 MTG High HFSC replicate showed poor correlation to the genome, so we have removed that one replicate from the analysis.

To infer enriched biological pathways or processes that are regulated in these subpopulations, we blasted (using blastp, blastx) transcripts that are significantly up/downregulated ($p<0.05$, $\geq$ 2fold) against Swissprot, Uniref90, and complete uniport database to identify homolog sequence ids. These ids were used as input for pathway & gene-ontology analysis. Corresponding blast annotations are mentioned in all the supplementary tables. We did pathway analysis & gene-ontology analysis for these up/down regulated transcripts using GSEA (Mootha et al., 2003; Subramanian et al., 2005). We used a customized perl script for all the analysis used in this study. We used R ggplot2 (Wickham, 2009), pHeatmap, and CummeRbund (Goff et al., 2012) library for plotting. Different planarian cell-type markers were obtained from the available single-cell transcriptome data (Fincher et al.,

2018; Plass et al., 2018; Van Wolfswinkel et al., 2014). Expression of these markers (FPKM values) from all the different cell populations is plotted as heatmaps using pHeatmap (https://cran.r-project.org/web/packages/pheatmap/index.html). Pseudotime analysis was performed with representative transcripts from the different populations using an available single-cell dataset (https://shiny.mdc-berlin.de/psca/).

Quantitative Real-Time PCR

Animals treated with either FCCP or DMSO (vehicle-control) at 3dpa were used for quantitating piwi-1 transcript levels. RNA was isolated using Trizol™ method as described above. 1 μg of the total was used to synthesize cDNA using PrimeScript 1st strand cDNA Synthesis Kit (Takara, Cat # 6110A) and Oligo dT primers. qPCR was performed using TB Green Premix Ex Taq II (Tli RNase H Plus) (Takara, Cat #RR820B) in Applied Biosystems. Data were normalized to Actin transcript levels and analysed by ΔΔCT method.

The following primers were used in the study.

Piwi-1 forward: ACCGTATTGAAACGTGAGCC

Piwi-1 reverse: TACACATCCCCCAGCTCTTC

Actin forward: GCTCCACTCAATCCAAAAGC

Actin reverse: TCAAATCTCTACCGGCCAAG

Bulk cell transplantation

Bulk cell transplantation in irradiated animals was carried out as described earlier with minor modifications (Davies et al., 2017; Wang et al., 2018). FACS sorted cells were centrifuged and resuspended in IPM 5% FBS media and kept in ice throughout the experiments. For colony expansion and long term survival experiments, 2 days post irradiated animals were used. The irradiated worm was placed ventral side up above a black filter paper placed in a cold plate. The injection was carried out using an Eppendorf femtojet 4x with a pressure of 0.8-

1.0 psi. Glass capillaries (length 3.5", ID: 0.53 mm, OD: 1.14 mm, Drummond Scientific, Inc. USA) were pulled using Sutter Instrument model P-1000. Cell suspensions were loaded onto the pulled capillaries using a mouth pipette (Sigma, A5177-5EA). For colony expansion assay, ~1000 cells/µL were injected and for long-term survival assay, ~1500 cells/µL was injected into the post gonopore midline of sexual hosts. For transplantation after in vitro culture ~2500 cells were injected per transplant. The transplant hosts and the uninjected control worms were maintained in gentamicin 50 µg/mL in 6 well plates with planaria water changes every two days. After 60 days, the surviving worms were fed with beef liver and amputated to regenerate twice. The fission activity of the rescued worms was monitored.

References

Bolger, A.M., Lohse, M., and Usadel, B. (2014). Trimmomatic: A flexible trimmer for Illumina sequence data. Bioinformatics *30*, 2114-212-.

Davies, E.L., Lei, K., Seidel, C.W., Kroesen, A.E., Mckinney, S.A., Guo, L., Robb, S.M.C., Ross, E.J., Gotting, K., and Sa, A. (2017). Embryonic origin of adult stem cells required for tissue homeostasis and regeneration. *6:e21052*, 1–35.

Fincher, C.T., Wurtzel, O., de Hoog, T., Kravarik, K.M., and Reddien, P.W. (2018). Cell type transcriptome atlas for the planarian Schmidtea mediterranea. Science (80-.). *360*.

Goff, L.A., Trapnell, C., and Kelley, D. (2012). CummeRbund: visualization and exploration of Cufflinks high-throughput sequencing data. R Packag. Version 2.0.

Grohme, M.A., Schloissnig, S., Rozanski, A., Pippel, M., Young, G.R., and Winkler, S. (2018). The genome of Schmidtea mediterranea and the evolution of core cellular mechanisms. Nature *554*, 56–61.

Guo, T., Peters, A.H.F.M., and Newmark, P.A. (2006). A bruno-like Gene Is Required for Stem Cell Maintenance in Planarians. Dev. Cell *11*, 159–169.

Kim, D., Langmead, B., and Salzberg, S.L. (2015). HISAT: A fast spliced aligner with low memory requirements. Nat. Methods *12*, 357–360.

King, R.S., and Newmark, P.A. (2013). In situ hybridization protocol for enhanced detection of gene expression in the planarian Schmidtea mediterranea. BMC Dev. Biol. *13*, 1–16.

Labbé, R.M., Irimia, M., Currie, K.W., Lin, A., Zhu, S.J., Brown, D.D.R., Ross, E.J., Voisin, V., Bader, G.D., Blencowe, B.J., et al. (2012). A Comparative transcriptomic analysis reveals conserved features of stem cell pluripotency in planarians and mammals. Stem Cells *30*, 1734–1745.

Lei, K., McKinney, S.A., Ross, E.J., Lee, H.-C., and Alvarado, A.S. (2019). Cultured pluripotent planarian stem cells retain potency and express proteins from exogenously introduced mRNAs. BioRxiv *573725*.

Mootha, V.K., Lindgren, C.M., Eriksson, K.F., Subramanian, A., Sihag, S., Lehar, J., Puigserver, P., Carlsson, E., Ridderstråle, M., Laurila, E., et al. (2003). PGC-1α-responsive genes involved in oxidative phosphorylation are coordinately downregulated in human diabetes. Nat. Genet. *34*, 267–273.

Plass, M., Solana, J., Alexander Wolf, F., Ayoub, S., Misios, A., Glažar, P., Obermayer, B., Theis, F.J., Kocks, C., and Rajewsky, N. (2018). Cell type atlas and lineage tree of a whole complex animal by single-cell transcriptomics. Science (80-.). *360*.

Subramanian, A., Tamayo, P., Mootha, V.K., Mukherjee, S., Ebert, B.L., Gillette, M.A., Paulovich, A., Pomeroy, S.L., Golub, T.R., Lander, E.S., et al. (2005). Gene set enrichment analysis: A knowledge-based approach for interpreting genome-wide expression profiles. Proc. Natl. Acad. Sci. U. S. A. *102*, 15545–15550.

Trapnell, C., Williams, B.A., Pertea, G., Mortazavi, A., Kwan, G., Van Baren, M.J., Salzberg, S.L., Wold, B.J., and Pachter, L. (2010). Transcript assembly and quantification by RNA-Seq reveals unannotated transcripts and isoform switching during cell differentiation.

Nat. Biotechnol. *28*, 511–515.

Trapnell, C., Hendrickson, D.G., Sauvageau, M., Goff, L., Rinn, J.L., and Pachter, L. (2013). Differential analysis of gene regulation at transcript resolution with RNA-seq. Nat. Biotechnol. *31*, 46–53.

Tu, K.C., Cheng, L.C., Vu, H.T.K., Lange, J.J., McKinney, S.A., Seidel, C.W., and Sánchez Alvarado, A. (2015). Egr-5 is a post-mitotic regulator of planarian epidermal differentiation. Elife e10501.

Wang, I.E., Wagner, D.E., and Reddien, P.W. (2018). Clonal analysis of planarian stem cells by subtotal irradiation and single-cell transplantation. In Planarian Regeneration . Humana Press, New York, NY., pp. 479–495.

Wickham, H. (2009). ggplot2 Elegant Graphics for Data Analysis. Springer.

Van Wolfswinkel, J.C., Wagner, D.E., and Reddien, P.W. (2014). Single-cell analysis reveals functionally distinct classes within the planarian stem cell compartment. Cell Stem Cell *15*, 326–339.

Zeng, A., Li, H., Guo, L., Gao, X., McKinney, S., Wang, Y., Yu, Z., Park, J., Semerad, C., Ross, E., et al. (2018). Prospectively Isolated Tetraspanin+Neoblasts Are Adult Pluripotent Stem Cells Underlying Planaria Regeneration. Cell 1593–1608.
